# Nutritional Status and Dietary Intake of School-Age Children and Early Adolescents: Systematic Review in a Developing Country and Lessons for the Global Perspective

**DOI:** 10.3389/fnut.2021.739447

**Published:** 2022-02-02

**Authors:** Durray Shahwar A. Khan, Jai K. Das, Shagufta Zareen, Zohra S. Lassi, Afsah Salman, Muhammad Raashid, Aftab A. Dero, Aijaz Khanzada, Zulfiqar A. Bhutta

**Affiliations:** ^1^Division of Women and Child Health, Aga Khan University Hospital, Karachi, Pakistan; ^2^Institute of Global Health and Development, Aga Khan University, Karachi, Pakistan; ^3^Policy and Strategic Planning Unit, Ministry of Health, Government of Punjab, Lahore, Pakistan; ^4^Faculty of Health and Medical Sciences, Robinson Research Institute, The University of Adelaide, Adelaide, SA, Australia; ^5^Centre for Global Child Health, The Hospital for Sick Children (SickKids), Toronto, ON, Canada; ^6^Government Services Hospital, Karachi, Pakistan; ^7^Ministry of Health, Government of Sindh, Karachi, Pakistan

**Keywords:** malnutrition, dietary intake, school-going children, adolescents, double burden of malnutrition

## Abstract

**Background:**

The prevalence of double burden of malnutrition (DBM) is high in low- and middle-income countries (LMICs). Data on malnutrition trends is present for children <5 years of age, however the data for school-going children and adolescents aged 5–15 years is scarce.

**Objective:**

This systematic review presents the pooled prevalence of nutritional status and dietary intake among school-going children and adolescents (5–15 years of age) in an LMIC of Pakistan and the perspective for broader global nutrition in this age group.

**Methods:**

An electronic search of databases was run on Pubmed and Medline (via Ovid) along with gray literature and archives of local scientific journals till 2nd January 2021. Studies meeting the eligibility criteria were included and relevant data were extracted, and a pooled proportional analysis was performed.

**Results:**

A total of 51 studies including 62,148 children of 5–15 years met the inclusion criteria, of which 30 studies reported on anthropometric indices alone, eight on dietary intake patterns while 13 reported both. All of the included studies had a cross-sectional study design. There were 20 studies from the province of Punjab, 15 from Sindh, eight from Khyber Pakhtoonkhwa, two from Balochistan, and three from multiple cities across Pakistan. The pooled proportional analysis showed that the proportion of underweight children and adolescents was 25.1% (95% CI 17.3–33.7%); stunting 23% (95% CI 11.8–36.7%); wasting 24% (95% CI 15.2–34%); thinness 12.5% (95% CI 9.4–16.1%); overweight 11.4% (95% CI 7.2–16.3%); and obesity 6.9% (95% CI 3–12%). A relatively high intake of carbohydrates, soft drinks, and sweets/chocolates; and a low intake of protein-rich foods, fruits, and vegetables, compared to the recommended daily allowance (RDA), was reported.

**Conclusion:**

The limited data suggests the presence of DBM amongst children aged 5–15 years and also identified that dietary intake patterns are not meeting the recommended allowance. This review highlights the gaps and the need for larger, well-designed studies for this age group with the representation of different contexts and the need for similar studies in various LMICs, so that appropriate actions be deliberated and appropriate programs should be designed focusing on this vital population.

## Introduction

Populations in which there is co-existence of under- and over-nutrition are known to be facing the double burden of malnutrition (DBM) (1). According to Global Nutrition Reports 2018, one in three people suffer from malnutrition, one in 20 children complain of hunger, and one in every five deaths around the world is attributed to poor diet (2). DBM is more prevalent in low- and middle-income countries (LMIC), with a higher prevalence in poorer LMICs (3). It is especially prevalent in sub-Saharan Africa, South-East Asia, and the Pacific (3). The progress in the reduction of the burden of malnutrition worldwide has been slow and it is therefore advised to collect population-specific data to better understand the nutrition dynamics across the world and to allow the nutritional needs of communities to be addressed adequately (2, 4).

An issue being ignored is malnutrition trends in children over the age of 5 years. The World Health Organization (WHO) reports 1.8 billion children to be in the age bracket of 5–15 years worldwide, with 90% of this population residing in LMICs (5). There is no consistent terminology used to describe children age 5–15 years which proves the narrow focus on younger children and neglect of this age group, however, children age 5–10 years are often referred to as school-going children (6), while adolescent has been defined by the WHO as children aged 10–19 years, with early adolescent defined to be in an age bracket of 10–14 years and late adolescent between 15 and 19 years (7). Whether DBM exists in this age bracket and to what extent is a query that is yet to be adequately explored.

In 2011, the United Nations Children's Fund (UNICEF) published a report stating that adolescence provides a second window of opportunity to improve the nutritional status of children and prevent future health consequences of malnourishment (8). However, nutritional challenges occur throughout the life cycle of an individual, therefore, nutritional needs through each phase must be assessed and addressed adequately (7), especially school-going children and adolescents age 5–15 years. Mental and physical development continues through this age bracket and it gives individuals a chance to improve their nutritional deficiencies, thereby preventing impairment of growth, development, and cognitive achievement (8). It is known that major developmental and physical changes occur within the early adolescence phase. This includes growth spurt, development of sex organs, secondary sexual characteristics, and, according to recent neuroscientific research, significant increase and reorganization in the neuronal network (8). A relatively newer concept referred to as developmental origins of adult health and disease (DOHaD) postulates that poor nutrition during the early phases of life is associated with chronic illnesses in adulthood (9). The current scarcity of data on school-going children and adolescents and now, with the increase in child survival rates, the number of children entering their second decade is increasing and their health and nutritional needs compel attention.

The WHO proposes strategic guidance and planning on actions for child health in the South-East Asian Region (SEAR), however, it is limited to adolescents alone (10). It has been reported that 20% of the population in the South-East Asian Region comprises of adolescents, which make up to a total of 360 million adolescents in the region (11). The process used by WHO in developing strategic guidance for improving adolescent health was by first conducting relevant reviews under national, regional, or global categories, followed by surveys in those regions to identify lessons learned and proposals for future actions. They also took input from experts in the field and then finally developed the guidance (10). This process should be adopted by other LMICs to identify the gaps and make the necessary interventions for improvement.

It is imperative that children above 5 years of age be assessed for undernutrition, overnutrition, and nutritional deficiencies, and therefore this systematic review aims to present a narrative on the trends of nutritional status and dietary intake patterns among school-going children and adolescents 5–15 years of age across Pakistan with a broader commentary related to global nutrition status, and challenges in this age group across other LMICs. This systematic review can be used as an example to synthesize the available literature and identify gaps in nutritional status and dietary intake patterns amongst school-going children and early adolescents aged 5–15 years in other LMICs.

## Materials and Methods

### Types of Studies and Participants

We included observational studies (prospective and retrospective cohort, and cross-sectional studies) reporting data on nutritional status and dietary intake and their association to gender, locale (urban vs. rural), school type (government vs. private), family income, and lifestyle (sedentary vs. active) amongst school-going children and early adolescents aged 5–15 years in Pakistan. We also included studies reporting nutrition trends in children affected by natural disasters or employed as laborers. Studies that assessed dietary intake and prevalence of malnutrition amongst children were included, as long as data on our age group of interest was also present. Studies exclusively assessing children with known co-morbidities or on Pakistani children living abroad were excluded. We included studies that were published during and after the year 2000 to ensure we get information on current trends, with the last date of the search conducted on the 2nd of January 2021.

### Types of Outcomes

We included studies that met our eligibility criteria and reported outcomes on anthropometric indices or dietary intake, such as underweight [weight-for-age Z (WAZ) score < −2 SD], stunting [height-for-age Z (HAZ) score < −2 SD], wasting [weight-for-height Z (WHZ) score< −2 SD], thinness (BMI-for-age < −2 SD), overweight (BMI-for-age > +1 SD), obesity (BMI-for-age > +2 SD), macro/micronutrient deficiencies, food, and nutrient intake. We also extracted the associations of these outcomes, such as gender, socio-economic status, private vs. government schools, family income, and sedentary lifestyles.

### Search Methods

We conducted an electronic literature search until 2nd January 2021 using Pubmed, Medline (via Ovid), and Google Scholar. Gray literature search was conducted on databases from the WHO, UNICEF, Food and Agriculture Organization (FAO), World Food Programme (WFP), Global Alliance for Improved Nutrition (GAIN), Scaling Up Nutrition (SUN), Action Against Hunger, International Food Policy Research Institute (IFPRI), and Google web. We also searched the archives of local journals [Journal of Pakistan Medical Association (JPMA) and Journal of Ayub Medical College (JAMC)] separately and went through the reference lists of included studies. We included articles that provided data on nutritional status and dietary intake patterns and their associations amongst school-aged children and early adolescents aged 5–15 years in Pakistan. Nutritional status was defined as “a physiological state of an individual, which results from the relationship between nutrient intake and requirements, and from the body's ability to digest, absorb and use these nutrients” (12).

The completed search strategy used for Pubmed and Medline (via Ovid) is presented as Supplementary Tables 1a,b). The following MeSH terms and their variants were used for our search strategy: “Nutritional Status” OR “Nutrition Assessment” OR “Diet” OR “Micronutrients” AND (“Schools” OR “Child” OR “Child/education” OR “Adolescent”) AND (“Pakistan” OR “South Asia”). Studies conducted by the same author on the same population were scrutinized for overlapping data and the studies with the inclusion of more relevant variables were chosen. There were no language restrictions placed while screening articles.

### Data Collection and Analysis

Two reviewers (DSK and JKD) screened titles and abstracts for eligibility using EndNote X8 (13). We retrieved full texts of the remaining articles and examined them based on our eligibility criteria. Studies that fulfilled the inclusion and exclusion criteria were selected for this review. Any conflicts regarding article selection were resolved through mutual consensus. We extracted data on Microsoft Excel from the included studies on variables including study background (province, city), population, age group, sample size, setting (rural vs. urban, school vs. community, government vs. private schools), socioeconomic status, anthropometric indices (underweight, stunting, wasting, thinness, overweight and obesity), dietary intake patterns and associations (14).

Data were analyzed and pooled prevalence was performed on the Joanna Briggs Institute (JBI) SUMARI software (15). The meta-analysis pooled overall prevalence using Dersimonian and Laird random-effect meta-analysis after transforming data using Freeman-Tukey transformation arcsine square root transformation. The review pooled overall means and proportion for the age group of 5–15 years and reported their 95% confidence intervals (CI) and the percentage of variation across studies that is due to heterogeneity rather than chance using *I*^2^ statistics. Studies with participants of age 0–19 years, from which data specifically for 5–15 years age group could not be extracted, were placed in the age category of either 5–19 years or 0–19 years; and pooled separately. We also pooled performed subgroup analysis based on gender, geographic setting i.e., urban/rural, provinces, natural disaster, special population i.e., children who were laborers, school attended (private or government), and socio-economic class, for children age 5–15 years.

We used the National Institute of Health (NIH)—National Heart, Lung, and Blood Institute (NHLBI) quality assessment tool for cross-sectional studies to assess the quality and potential risk of bias for all the included studies (16). This tool helps evaluate the internal validity of a study, hence ensuring that the results are truly due to the exposure being evaluated.

This systematic review follows the guidelines recommended by the Preferred Reporting Items for Systematic Reviews and Meta-Analyses (PRISMA) (17). The PRISMA checklist is presented in Supplementary Table 2.

## Results

**Figure 1:**
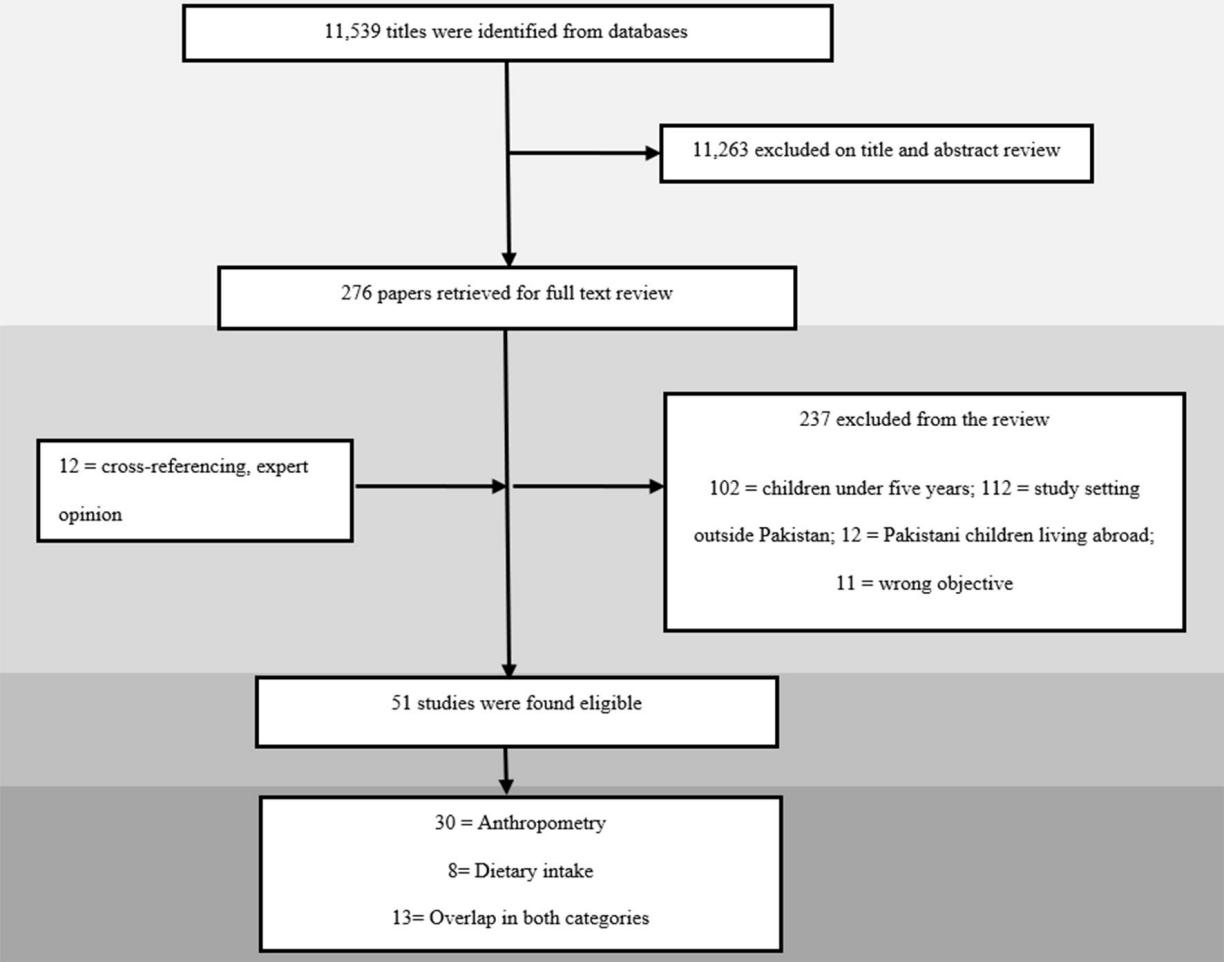
Search flow diagram.

Our electronic search for all databases yielded a total of 11,539 articles that underwent title and abstract screening. A total of 276 articles were selected for full-text review, of which 39 met the eligibility criteria. Through cross-referencing of included articles and local journals, another 12 articles were added, leading to a total of 51 studies being selected for inclusion as depicted in Figure 1. Results were reported according to two categories, namely “anthropometry” and “dietary intake”.

### Description of Included Studies

A total of 51 studies were included, all of which had a cross-sectional study design (18–68). The studies were conducted between the years 2002 and 2020 in different cities across Pakistan. Twenty-eight studies reported data specifically on children between 5 and 15 years of age (19, 21–23, 25–29, 31, 32, 35, 38, 44, 45, 47–50, 52, 54, 55, 59, 60, 62–64, 67). The remaining 23 studies had children in our age group of interest but beyond it too, with seven reporting data on children between 0 and 19 years of age (24, 34, 51, 56, 58, 61, 65) and 16 reporting on children 5 and 19 years of age (18, 20, 30, 33, 36, 37, 39–43, 46, 53, 57, 66, 68). There were only five studies that reported data specifically on children in the 5 to 10 age group (29, 34, 64–66) and only three studies in the 10 to 15 years age group (21, 24, 47). Province wise; 20 studies were conducted in Punjab (19, 22, 24, 28, 29, 31, 39, 40, 44, 46, 52–54, 58, 65, 67), 15 in Sindh (18, 20, 21, 23, 30, 32, 36–38, 43, 45, 49, 55, 63), eight in Khyber Pakhtoonkhwa (KP) (26, 34, 35, 47, 48, 50, 61, 64), two in Balochistan (25, 51), four from the federal capital (27, 42, 66, 68), and three from multiple cities across Pakistan (41, 56, 57). The remaining three studies failed to report their location (33, 59, 62).

A total of 35 studies were conducted in urban areas (18–46, 52, 63, 65–68), while five were conducted in rural areas (47–51), six in both (53–58), and the remaining five did not report their setting (59–62, 64). Forty studies were carried out in a school setting (15, 16, 19–22, 24–40, 42, 44–52, 55, 58, 59, 61, 63–65), nine in community setting (21, 27, 45, 47, 57, 58, 60, 61, 66), and two studies did not specify (22, 64). Of the forty conducted in schools, 22 studies were conducted across both government and private schools (18, 20, 23, 24, 26, 28, 29, 31, 35, 37, 38, 40, 43, 44, 48, 52–54, 56, 62, 67, 68), seven in private schools exclusively (19, 25, 30, 36, 39, 41, 65), and five were carried out in government schools (32, 33, 49, 50, 55). Six studies did not specify their study setting (34, 42, 46, 51, 59, 63). There were two studies which reported nutritional status amongst children affected by natural disasters (47, 50) and two on child laborers (45, 68). For the age group of 5–15 years particularly, 17 studies reported anthropometric indices with respect to gender (19, 21–23, 26, 28, 29, 32, 35, 38, 44, 48, 49, 54, 60, 62, 63), 18 with respect to geographic setting; urban or rural (19, 21–23, 26–29, 32, 35, 38, 44, 48, 49, 52, 63, 67), three with respect to socioeconomic status (29, 35, 52) and eight with respect to school attended; private or government (19, 26, 28, 32, 44, 49, 62, 65).

The included studies in this review targeted 62,148 individuals. Two studies had a sample size of >10,000 (56, 57), 14 studies had a sample size of 1,000–9,999 individuals (21, 24, 28, 29, 31, 37, 42, 47–49, 51, 54, 55, 58), six had a sample size of 500–999 (18, 30, 33, 38, 53, 60), 27 studies between 100 and 499 (19, 20, 22, 23, 25–27, 32, 34–36, 39–41, 43–46, 50, 52, 59, 62, 64–68), and two studies with a sample size of <100 individuals (61, 63).

Of the selected 51 studies, 30 reported data on anthropometric indices only (18, 23, 26–29, 33–35, 37–39, 43–45, 47–52, 54, 56, 60–66), eight reported data on dietary intake alone (20, 25, 31, 41, 53, 55, 57, 68), while 13 reported both, anthropometric indices and dietary intake patterns across our population of interest (19, 21, 22, 24, 30, 32, 36, 40, 42, 46, 58, 59, 67). The characteristic of each included study is presented briefly in Table 1 below with a detailed version included as Supplementary Table 3.

**Table 1:** Brief overview of characteristics of included studies.

**#**	**References**	**Study design**	**City**	**Province**	**Target population**	**Setting (urban/rural and SES)**	**Sample size**	**Anthropometry classification/Dietary assessment tool**	**Results**
1	Ahmed et al. (18)	Cross-sectional	Hyderabad	Sindh	9–17 Class 6–10	Private and government schools in an urban setting. Children from all socioeconomic backgrounds	501	WHO	Overweight: 8%; Obese: 12%
2	Afzal et al. (53)	Cross-sectional	Okara	Punjab	13–18 Girls only	Private and government schools in urban and rural setting	850	Questionnaire	Dietary intake patterns
3	Akbar et al. (59)	Cross-sectional	N/A	N/A	6–11	Schools	150	- Waldrew Classification - FFQ	Underweight: 45.5%; Overweight: 11%; Obese: 2.46%; Dietary intake patterns
4	Anwar et al. (19)	Cross-sectional	Lahore	Punjab	10–14 Class 6–7	Private schools in an urban setting	293	- WHO - Questionnaire	Overweight: 21.8%; Obese: 11.9%
5	Anwer and Awan (54)	Cross-sectional	Faisalabad	Punjab	6–12	Private and government schools in urban and rural setting. Children from low- and middle-socioeconomic backgrounds	2,042	Jelliffe's Classification	Wasting: 32.9%; Stunting: 36.1%; Underweight: 45.3%
6	Aziz et al. (55)	Cross-sectional	Dadu, Kambar, Jacobabad, Karachi, Sukkur Kashmore Khairpur, Larkana, Shahdadkot	Sindh	7–11	Government schools in urban and rural setting	1,109	FFQ	Dietary intake patterns
7	Aziz et al. (30)	Cross-sectional	Karachi	Sindh	6–17	Private schools in urban setting	398	- CDC -24 h dietary recall	Overweight: 19.35%; Obese: 6%; Dietary intake patterns
8	Aziz et al. (30)	Cross-sectional	Karachi Lahore Quetta	Multiple	6–18	Private schools in urban setting	652	24 h dietary recall	Dietary intake patterns
9	Aziz et al. (56)	Cross-sectional	Cities across Pakistan	Multiple	3–16	Private and government schools in urban and rural setting. Children from all socioeconomic backgrounds	12,837	CDC	Stunting: 14%; Obese: 5.1%
10	Aziz and Hosain (57)	Cross-sectional	Cities across Pakistan	Multiple	6–16	Communities in an urban and rural setting	11,237	24 h dietary recall	Dietary intake patterns
11	Babar et al. (52)	Cross-sectional	Lahore	Punjab	6–11	Private and government schools in urban setting. Children from all socioeconomic backgrounds	161	NHANES	Underweight: 29.8%; Overweight: 13.7%; Obese: 8.1%
12	Basit et al. (63)	Cross-sectional	Karachi	Sindh	8–10	Schools in an urban setting. Children from middle socioeconomic background	92	Not stated	Stunting: 2%; Underweight: 12%; Obese: 5%
13	Batool et al. (65)	Cross-sectional	Faisalabad	Punjab	4–12	Private schools in urban setting. Children from middle socioeconomic background	432	WHO	Stunting: 45.8%; Underweight: 25.4%
14	Fatima et al. (66)	Cross-sectional	Islamabad	Federal Capital Territory	11–19	Community in an urban setting	150	Not stated	Underweight: 36.3%; Overweight: 7.1%; Obese: 12.2%
15	Hall and Kirby (47)	Cross-sectional	NWFP	KP	5–14	Community in a rural setting. Children from low socioeconomic background	2,032	WHO	Thinness: 12%; Stunting: 43%; Underweight: 34%
16	ul Haq et al. (48)	Cross-sectional	Hazara Division	KP	5–14	Private and government schools in a rural setting	3,200	Not stated	Obese: 4.78%
17	Hayyat (67)	Cross-sectional	Lahore	Punjab	5–12	Private and government schools in urban setting	240	WHO	Underweight: 1.3%; Overweight: 24.6%; Obese: 50%
18	Iqbal et al. (60)	Cross-sectional	Karachi Thatta Hyderabad	Sindh	5–14	Community setting	634	WHO	Wasted: 30%; Stunting: 15.5%
19	Iqbal et al. (68)	Cross-sectional	Islamabad Rawalpindi	Punjab/Federal Capital	11–16	Private and government schools in an urban setting	332	Questionnaire	Dietary intake patterns
20	Irshad et al. (61)	Cross-sectional	Kohistan	KP	0–14	Community setting	80	WHO	Thinness: 3.75%; Stunting: 3.75%; Underweight: 26.25%
21	Ishaque et al. (20)	Cross-sectional	Karachi	Sindh	13–16	Private and government schools in an urban setting	431	- WHO - Questionnaire	Overweight + Obese = 28%; Dietary intake patterns
22	Jafar et al. (21)	Cross-sectional	Karachi	Sindh	5–14	Community in an urban setting	1,675	FFQ	Stunting: 14.6%; Underweight: 27.9%; Dietary intake patterns
23	Kauser and Naz (22)	Cross-sectional	Sargodha	Punjab	12–15	Urban setting	200	FFQ	Underweight: 53.5%; Dietary intake patterns
24	Khan et al. (23)	Cross-sectional	Karachi	Sindh	7–12	Private and government schools in an urban setting. Children from all socioeconomic backgrounds	240	NCHS	Stunting: 26.3%; Underweight: 34.9%; Obese: 0.8%
25	Khan et al. (24)	Cross-sectional	Multan	Punjab	3–18	Private and government schools in an urban setting. Children from all socioeconomic backgrounds	1,872	- WHO - Questionnaire	Overweight: 10%; Obese: 5%; Dietary intake pattern
26	Khan et al. (25)	Cross-sectional	Quetta	Balochistan	11–16 (Mean age 14; grade 6 to 9)	Private schools in an urban setting	423	FFQ	Dietary intake patterns
27	Khuwaja et al. (49)	Cross-sectional	Thatta Badin Mirpur Khas Tharparkar	Sindh	6–12	Government schools in a rural setting	1,915	NCHS	Stunting: 16.5%
28	Marwat et al. (26)	Cross-sectional	Abbottabad	KP	7–13	Private and government schools in an urban setting	200	NA	Underweight: 0.27%; Overweight: 0.05%; Obese: 0.06%
29	Mian et al. (27)	Cross-sectional	Islamabad	Federal Capital	5–10	Community in urban setting	200	Not stated	Wasting: 13%; Stunting: 35%; Underweight: 29.5%
30	Mohsin et al. (50)	Cross-sectional	Nowshera	KP	6–14	Government schools in a rural setting	163	WHO	Underweight: 39.8%
31	Mushtaq et al. (28)	Cross-sectional	Lahore	Punjab	5–12	Private and government schools in an urban setting. Children from all socioeconomic backgrounds	1,860	WHO	Overweight: 17%; Obese: 7.5%
32	Mushtaq et al. (29)	Cross-sectional	Lahore	Punjab	5–12	Private and government schools in an urban setting. Children from all socioeconomic backgrounds	1,860	WHO	Thinness: 10%; Stunting: 8%
33	Mushtaq et al. (31)	Cross-sectional	Lahore	Punjab	5–12	Private and government schools in an urban setting. Children from all socioeconomic backgrounds	1,860	Questionnaire	Dietary intake patterns
34	Mustafa et al. (51)	Cross-sectional	Lasbela	Balochistan	4–15	Schools in a rural setting	6,363	WHO	Overweight: 5.8%; Obese: 3.3%
35	Ponum et al. (58)	Cross-sectional	Multan	Punjab	4–18	Communities and urban and rural setting	1,420	- WHO - Questionnaire	Stunting: 24.93%
36	Qureshi et al. (32)	Cross-sectional	Hyderabad	Sindh	5–18	Government schools in an urban setting	422	- WHO - Questionnaire	Thinness: 13.7%; Underweight: 18.3%; Dietary intake patterns
37	Rahman et al. (33)	Cross-sectional	N/A	N/A	12–16	Government schools in an urban setting	661	WHO	Overweight: 7.7%; Obese: 1%
38	Ramzan et al. (35)	Cross-sectional	Dera Ismail Khan	KP	6–11	Private and government schools in an urban setting	322	CDC	Underweight: 5.59%
39	Rehman (34)	Cross-sectional	Peshawar	KP	4–12	Schools in an urban setting	400	Not stated	Underweight: 30%; Overweight: 7%; Obese: 0%
40	Riaz et al. (62)	Cross-sectional	N/A	N/A	5–10	Schools in urban and rural setting	344	CDC	Stunting: 10.7%; Underweight: 38.7%; Overweight: 0.3%
41	Rizwan et al. (36)	Cross-sectional	Karachi	Sindh	11–17	Private schools in an urban setting	339	- Not stated -FFQ	Overweight + Obesity = 17.7%; Dietary intake patterns
42	Sadiq et al. (37)	Cross-sectional	Karachi	Sindh	7–18	Private and government schools in an urban setting. Children from all socioeconomic backgrounds	1244	WHO	Underweight: 5.2%; Overweight: 9.2%; Obese: 6.4%
43	Shahid et al. (38)	Cross-sectional	Karachi	Sindh	8–14	Private and government schools in an urban setting	500	Not stated	Underweight: 21.4%; Overweight: 8.2%; Obese: 6.6%
44	Shahid et al. (39)	Cross-sectional	Lahore	Punjab	10-16	Private schools in an urban setting	197	Not stated	Obese: 18.2%
45	Shaukat et al. (40)	Cross-sectional	Lahore	Punjab	6–16	Private and government schools in an urban setting	370	- CDC - Questionnaire	Underweight: 18.4; Overweight: 35.1%; Dietary intake patterns
46	Siddique et al. (64)	Cross-sectional	Abbottabad	KP	5–10	Not specified	408	Not stated	Stunting: 0.7%; Underweight: 2.98%
47	Sultana et al. (42)	Cross-sectional	Rawalpindi Islamabad	Punjab/Federal Capital	10–18	Schools in an urban setting	1,360	- Not stated - Questionnaire	Overweight: 2%; Obese: 0.2%; Dietary intake patterns
48	Warraich et al. (43)	Cross-sectional	Karachi	Sindh	11–17	Private and government schools in an urban setting. Children from all socioeconomic backgrounds	284	Not stated	Underweight: 52%; Overweight: 8%; Obese: 6%
49	Zahid et al. (44)	Cross-sectional	Faisalabad	Punjab	5–12	Private and government schools in an urban setting. Children from all socioeconomic backgrounds	200	WHO	Wasting: 20%; Stunting: 25.5%; Underweight: 26.5%; Overweight: 8.5%; Obese: 9.5%
50	Zainab et al. (45)	Cross-sectional	Karachi	Sindh	10–14	Communities in urban setting	385	WHO	Thinness: 8.1; Overweight: 17.9%; Obese: 3.6%
51	Zaman et al. (46)	Cross-sectional	Sialkot	Punjab	13–16	Schools in the urban setting	328	- WHO - 3-Day dietary recall	Overweight: 18.9%; Obese: 3.96%; Dietary intake patterns

*CDC, Center for Disease Control; FFQ, Food Frequency Questionnaire; KP, Khyber Pakhtoonkhwa; NCHS, National Center for Health Sciences; WHO, World Health Organization*.

### Quality of Studies

Quality assessment using NHLBI tool for cross-sectional studies, as presented in Supplementary Table 4 and briefly as Table 2, showed that all studies had clearly stated their objective and had a participation rate of >50%, with all the subjects selected from the same population. 88.2% of studies had specified and defined their population, while only 33.3% had justified sample size calculation. Since all the studies were cross-sectional, exposure was not measured prior to outcomes, studies were assessed at one point in time and therefore had no follow-ups. Outcomes were defined by 74.5% of the studies, while none of the studies reported outcomes to be blinded to assessors. 25.5% of studies measured confounding variables and adjusted them statistically to assess associations to the outcomes.

**Table 2:** Summary of NHLBI quality assessment.

**Assessment query**	**Response (%)**
Was the research question or objective in this paper clearly stated?	Yes; 100%
Was the study population clearly specified and defined?	Yes; 100%
Was the participation rate of eligible persons at least 50%?	Yes; 100%
Were all the subjects selected or recruited from the same or similar populations (including the same time period)? Were inclusion and exclusion criteria for being in the study prespecified and applied uniformly to all participants?	Yes; 100%
Was a sample size justification, power description, or variance and effect estimates provided	Yes; 33.3%
For the analyses in this paper, were the exposure(s) of interest measured prior to the outcome(s) being measured	NA
Was the timeframe sufficient so that one could reasonably expect to see an association between exposure and outcome if it existed	NA
For exposures that can vary in amount or level, did the study examine different levels of the exposure as related to the outcome (e.g., categories of exposure, or exposure measured as continuous variable)?	NA
Were the exposure measures (independent variables) clearly defined, valid, reliable, and implemented consistently across all study participants	Yes; 17.6%
Was the exposure(s) assessed more than once over time?	No; 100%
Were the outcome measures (dependent variables) clearly defined, valid, reliable, and implemented consistently across all study participants?	Yes; 74.5%
Were the outcome assessors blinded to the exposure status of participants?	No; 100%
Was loss to follow-up after baseline 20% or less?	NA
Were key potential confounding variables measured and adjusted statistically for their impact on the relationship between exposure(s) and outcome(s)?	Yes; 25.5%

### Anthropometric Indices

**Figure 2:**
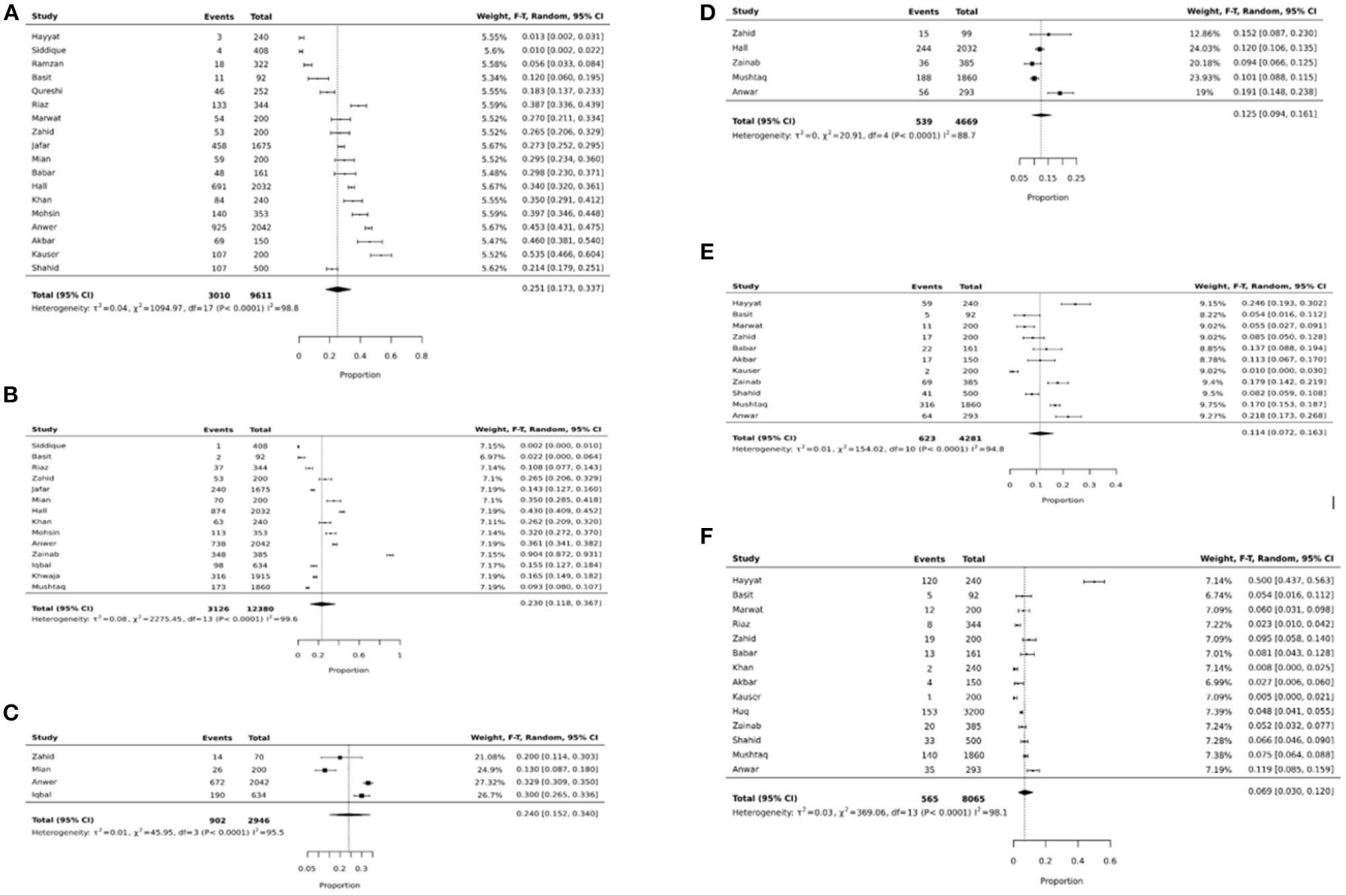
**(A)** Underweight pooled prevalence in 5–15 years. **(B)** Stunting pooled prevalence in 5–15 years. **(C)** Wasting pooled prevalence in 5–15 years. **(D)** Thinness pooled prevalence in 5–15 years. **(E)** Overweight pooled prevalence in 5–15 years. **(F)** Obese pooled prevalence in 5–15 years.

We identified 43 studies reporting data on anthropometric indices (18, 19, 30, 32–40, 42–52, 54, 56, 58, 59, 61–68). Our focus was to report the prevalence of malnutrition for the age group 5–15 years, however, some studies reported data beyond our age group of interest due to which an overall analysis, with overlapping data from 5 to 15 years age group, was also conducted for age groups zero to 19 and 5–19 years as depicted in Table 3, Supplementary Figures 1–3. The age group 5–19 was also separately reported to understand the overall malnutrition trends in children above 5 years of age. Anthropometric indices reported amongst school-going children and early adolescents age 5–15 years across provinces in Pakistan are depicted in Table 4, Figure 2, however, no data amongst children from Balochistan in this age group was available. Anthropometric indices with respect to gender, geographic setting (urban or rural), and type of school attended (private or government), along with indices of children affected by natural disasters (e.g., flood, earthquake, etc.) and child laborers in this age group have also been reported in Table 4, Supplementary Figures 4–9.

**Table 3:** Prevalence of Anthropometric Measures in Pakistan according to age groups.

	**Underweight**	**Stunting**	**Wasting**	**Thinness**	**Overweight**	**Obese**
5–15 years [Pooled prevalence (range); no. of studies; no. of participants; heterogeneity]	25.1% (17.3–33.7%) 18 studies; *n* = 9,611; *I*^2^ = 98.8	23% (11.8–36.7%) 14 studies; *n* = 12,380; *I*^2^ = 99.6	24% (15.2–34%) 4 studies; *n* = 2,946; *I*^2^ = 95.5	12.5% (9.4–16.1%) 5 studies; *n* = 4,669; *I*^2^ = 88.7	11.4% (7.2–16.3%) 11 studies; *n* = 4,281; *I*^2^ = 94.8	6.9% (3–12%) 14 studies; *n* = 8,065 ; *I*^2^ = 98.1
5–19 years [Pooled prevalence (range); no. of studies; no. of participants; heterogeneity]	26.9% (19–35.7%) 23 studies; *n* = 13,072; *I*^2^ = 99.1	23.1% (12.5–35.8%) 15 studies; *n =* 12,802; *I*^2^ = 99.6	24% (15.2–34%) 4 studies; *n =* 2,946; *I*^2^ = 95.5	12.7% (10.1–15.6%) 6 studies; *n =* 5,091; *I*^2^ = 85	11.4% (8–15.3%) 20 studies; *n =* 9,574; *I*^2^ = 96.6	6.8% (4.1–10.1%) 23 studies; *n =* 33,276; *I*^2^ = 99
0–19 years [Pooled prevalence (range); no. of studies; no. of participants; heterogeneity]	25.8% (18.5–33.7%) 26 studies; *n =* 15,457; *I*^2^ = 99.1%	22.5% (13.5–33.0%) 19 studies; *n =* 27,571; *I*^2^ = 99.7	19% (9.1–31.4%) 5 studies; *n =* 3,026; *I*^2^ = 97.4	12.7% (10.1–15.6%) 6 studies; *n =* 5,091; *I*^2^ = 85	10.8% (7.9–14.2%) 23 studies; *n =* 18,209; *I*^2^ = 97.5	6.3% (3.9–9.1%) 26 studies; *n =* 34,258; *I*^2^ = 98.8

**Table 4:** Setting specific anthropometric indices in children age 5–15 years.

	**5–15 years**	**Disaster affected**	**Child laborers**	**Gender**		**School system**		**Socioeconomic status**		**Setting**		**Province**			
				**Male**	**Female**	**Private**	**Government**	**Low**	**High**	**Urban**	**Rural**	**Sindh**	**Punjab**	**KP**	**Balochistan**
Underweight	25.1% (17.3–33.7%) 18 studies; *n =* 9,611; *I*^2^ = 98.8	36.3% (30.9–41.9) 2 studies; *n =* 2,385; *I*^2^ = 76		24.3% (17.1–32.4%) 9 studies; *n =* 2,853; *I*^2^ = 95.2	31.2% (21.7–41.5%) 10 studies; *n =* 3,122; *I*^2^ = 96.8	16.1% (12.9–19.6%) 4 studies; *n =* 470; *I*^2^ = 0	24.6% (16.1–33.4%) 4 studies; *n =* 678; *I*^2^ = 86.2	41% (30.3–52.2%) 1 study; *n =* 78; *I*^2^ = 0	11.2% (1.4–27.9%) 2 studies; *n =* 405; *I*^2^ = 92	22.7% (15.2–31.3%) 13 studies; *n =* 5,507; *I*^2^ = 97.9	64.7% (61.4–68%) 1 study; *n =* 817; *I*^2^ = 0	22.7% (15.9–30.4%) 5 studies; *n =* 2,759; *I*^2^ = 93.3	24.8% (12–40.2%) 8 studies; *n =* 4,160; *I*^2^ = 98.9	10.1% (0–47.4%) 2 studies; *n =* 608; *I*^2^ = 99.1	
Stunting	23% (11.8–36.7%) 14 studies; *n =* 12,380; *I*^2^ = 99.6	37.7% (27.3–48.7%) 2 studies; *n =* 2,385; *I*^2^ = 93.5	54.4% (0–100) 2 studies; *n =* 1,019; *I*^2^ = 99.9	15.5% (14.5–16.6%) 10 studies; *n =* 4,644; *I*^2^ = 94.9	19.1% (11.5–28%) 10 studies; *n =* 4,779; *I*^2^ = 97.9	17.8% (0.7–48.7%) 3 studies; *n =* 353; *I*^2^ = 97.5	22.4% (10.7–36.7%) 3 studies; *n =* 2,233; *I*^2^ = 95.4	16.7% (14–19.7%) 1 study; *n =* 651; *I*^2^ = 0	2% (0.7–4%) 1 study; *n =* 299; *I*^2^ = 0	18.6% (9.3–30.2%) 7 studies; *n =* 3,954; *I*^2^ = 98.4	27.8% (7.9–54%) 2 studies; *n =* 2,732; *I*^2^ = 99.4	13.7% (5.3–25.3) 4 studies; *n =* 3,922; *I*^2^ = 98.5	29.4% (17.1–43.3) 5 studies; *n =* 4,574; *I*^2^ = 98.7	0.2% (0–1%) 1 study; *n =* 408; *I*^2^ = 0	
Wasting	24% (15.2–34%) 4 studies; *n =* 2,946; *I*^2^ = 95.5		30% (26.5–33.6%) 1 study; *n =* 634; *I*^2^ = 0	19.7% (17.1–22.4%) 2 studies; *n =* 892; *I*^2^ = 0	27.7% (5–59.1%) 2 studies; *n =* 1,220; *I*^2^ = 92.3	20% (7.3–36.5%) 1 study; *n =* 30; *I*^2^ = 0	20% (8.8–34%) 1 study; *n =* 40; *I*^2^ = 0			19.1% (8.4–32.8) 3 studies; *n =* 1,625; *I*^2^ = 96	33.3% (30.1–36.6%) 1 study; *n =* 817; *I*^2^ = 0		21.8% (11.1–34.8) 3 studies; *n =* 2,312; *I*^2^ = 93.5		
Thinness	12.5% (9.4–16.1%) 5 studies; *n =* 4,669; *I*^2^ = 88.7	12% (10.6–13.5%) 1 study; *n =* 2,032; *I*^2^ = 0	9.4% (6.6–12.5%) 1 study; *n =* 385; *I*^2^ = 0	15% (7.8–23.9%) 3 studies; *n =* 1,158; *I*^2^ = 83	12.9% (8.4–18.2%) 3 studies; *n =* 1,094; *I*^2^ = 64.4	19.1% (14.8–23.8%) 1 study; *n =* 293; *I*^2^ = 0	28.8% (17.2–42%) 1 study; *n =* 52; *I*^2^ = 0	14.3% (11.7–17.1%) 1 study; *n =* 651; *I*^2^ = 0	5% (2.8–7.8%) 1 study; *n =* 299; *I*^2^ = 0	11.5% (7.2–16.7%) 2 studies; *n =* 1,959; *I*^2^ = 59.4			14.2% (8.9–20.6) 3 studies; *n =* 2,252; *I*^2^ = 85.5		
Overweight	11.4% (7.2–16.3%) 11 studies; *n =* 4,281; *I*^2^ = 94.8		17.9% (14.2–21.9%) 1 study; *n =* 385; *I*^2^ = 0	12.6% (7.1–19.4%) 5 studies *n =* 1,568; *I*^2^ = 89.5	8.5% (3.4–15.4%) 7 studies; *n =* 1,685; *I*^2^ = 93.6	17.1% (8.7–27.4%) 4 studies; *n =* 1,425; *I*^2^ = 93.6	13.1% (8.7–18.2%) 3 studies; *n =* 1,132; *I*^2^ = 62.4	2.6% (0–7.6%) 1 study; *n =* 78; *I*^2^ = 0	24.1% (15.4–33.9%) 1 study; *n =* 83; *I*^2^ = 0	10.7% (5.9–16.6%); 9 studies; *n =* 3,746; *I*^2^ = 95.6		7.6% (5.6–9.9) 2 studies; *n =* 592; *I*^2^ = 0	12.5% (6.5–20) 7 studies; *n =* 3,799; *I*^2^ = 96.9	5.5% (2.7–9.1%) 1 study; *n =* 200; *I*^2^ = 0	
Obese	6.9% (3–12%) 14 studies; *n =* 8,065; *I*^2^ = 98.1		5.2% (3.2–7.7%) 1 study; *n =* 385; *I*^2^ = 0	7.5% (4.9–10.5%) 6 studies; *n =* 3,168; *I*^2^ = 83.6	5% (2.6–8.1%) 7 studies; *n =* 3,285; *I*^2^ = 89.7	13.0% (10.9–15.3%) 4 studies; *n =* 1,425; *I*^2^ = 17.8	1.5% (0.3–3.4%) 3 studies; *n =* 1,128; *I*^2^ = 46.9	3.8% (0.5–9.5%) 1 study; *n =* 78; *I*^2^ = 0	12% (5.8–20%) 1 study; *n =* 83; *I*^2^ = 0	8.4% (2.9–16.2%) 10 studies; *n =* 3,986; *I*^2^ = 97.9	4.8% (4.1–5.5%) 1 study; *n =* 3,200; *I*^2^ = 0	3.8% (0.7–8.8%) 3 studies; *n =* 832; *I*^2^ = 86.4	10.5% (2.7–22.3%) 7 studies; *n =* 3,799; *I*^2^ = 98.8	4.8% (4.1–5.5%) 2 studies; *n =* 3,400; *I*^2^ = 0	

We noticed similar trends of pooled prevalence for children age 0–19 and 5–19 across all anthropometric indices as shown in Table 3. This could be because of the overlap in data across all three age groups.

The pooled prevalence of **underweight** amongst school-going children and adolescents age 5 to 15 years was 25.1% (95% CI: 17.3–33.7%; 18 studies; 9,611; *I*^2^: 98.8) (Table 4, Figure 2A). The prevalence was found to be higher amongst females (31.2%; 95% CI: 21.7–41.5%), children from government schools (24.6%; 95% CI: 16.1–33.4%), belonged to low SES (41%; 95% CI: 30.3–52.2%), from the province of Punjab (24.8% 95% CI: 12–40.2%), and Sindh (22.7%; 95% CI: 15.9–30.4%), and from disaster striken areas (36.3%; 95% CI: 30.9–41.9%) (Supplementary Figure 4).

The overall pooled prevalence of **stunting** in school-going children and adolescents age 5–15 years was 23% (95% CI: 11.8–36.7%; 14 studies; 12,380 participants; *I*^2^: 99.6) (Figure 2B). The prevalence was was higher amongst females (19.1%; 95% CI: 11.5–28%), children going to government schools (22.4%; 95% CI: 10.7–36.7%), those from a low SES (16.7%; 95% CI: 14–19.7%), and those who lived in rural areas (27.8%; 95% CI: 7.9–54%) (Table 4). The highest stunting pooled prevalence was noted to be amongst children from the province of Punjab (29.4%; 95% CI: 17.1–43.3%), those who were laborers (54.4%; 95% CI: 0–100%), and disaster striken areas (37.7%; 95% CI: 27.3–48.7%) (Supplementary Figure 5).

The pooled prevalence of **wasting** amongst school-going children and adolescents age 5–15 years was 24% (95% CI: 15.2–34%; 4 studies; 2,946 participants; *I*^2^: 95.5) (Figure 2C). Wasting was reported to be higher amongst females (27.7%; 95% CI: 5–59.1%), and those who lived in rural areas (33.3%; 95% CI: 30.1–36.6%). Data on wasting prevalence was only available for the province of Punjab with a pooled prevalence of 21.8% (95% CI: 11.1–34.8%) (Supplementary Figure 6).

The overall prevalence of **thinness** was 12.5% (95% CI: 9.4–16.1; 4 studies; 4,669 participants; *I*^2^: 88.7) (Figure 2D). Thinness was reported to be higher amongst males (15%; 95% CI: 7.8–23.9%), those attending government schools (28.8%; 95% CI: 17.2–42%), and those from a low SES (14.3% 95% CI: 11.7–17.1%) (Table 4). Data on thinness prevalence was only available for the province of Punjab with a pooled prevalence of 14.2% (95% CI: 8.9–20.6). For children from disaster-affected regions and child laborers, the pooled prevalence of thinness was reported to be 12% (95% CI: 10.6–13.5%) and 9.4% (95% CI: 6.6–12.5%), respectively (Supplementary Figure 7).

The overall **overweight** pooled prevalence for school-going children and adolescents age 5–15 years was 11.4% (95% CI: 7.2–16.3%; 11 studies; 4,281 participants; *I*^2^: 94.8) (Figure 2E). Overweight pooled prevalence was noted to be higher amongst males (12.6%; 95% CI: 7.1–19.4%), children going to private schools (17.1%; 95% CI: 8.7–27.4%), and those from a high SES (24.1%; 95% CI: 15.4–33.9%) (Table 4). Between provinces, the highest overweight prevalence was amongst children from Punjab (12.5%; 95% CI: 6.5–20%), followed by Sindh (7.6%; 95% CI: 5.6–9.9%) and the least in KP (5.5%; 95% CI: 2.7–9.1%) (Supplementary Figure 8).

The pooled prevalence on **obesity** was 6.9% (95% CI: 3–12%; 14 studies; 8,065 participants; *I*^2^: 98.1) (Figure 2F). The pooled prevalence of obesity was noted to be higher amongst males (7.5%; 95% CI: 4.9–10.5%), children attending private schools (13%; 95% CI: 10.9–15.3%), those from high SES (12%; 95% CI: 5.8–20%), and those living in urban areas (8.4%; 95% CI: 2.9–16.2%) (Table 4). The highest obesity pooled prevalence was reported amongst children from Punjab (10.5%; 95% CI: 2.7–22.3%), followed by KP (4.8%; 95% CI: 4.1–5.5%), and least in Sindh (3.8%; 95% CI: 0.7–8.8%). Only 5.2% (95% CI: 3.2–7.7%) obesity pooled prevalence was reported amongst child laborers (Supplementary Figure 9).

For the age group of 5–10 years, we could only calculate pooled prevalence for underweight which was 6.5% (95% CI: 2–13.1%; 5 studies, 1,569 participants, *I*^2^: 94.7%) and stunting at 4% (95% CI: 0 to 12.7%; 4 studies, 1,044 participants, *I*^2^:96%) (Supplementary Figure 10). While for the age group 10–15 years, pooled prevalence was only calculated for overweight at 5.9% (95% CI: 3.4 to 8.9%; 2 studies, 678 participants, *I*^2^: 56.8%) and obesity at 2.5% (95% CI: 0.5–5.8%; 2 studies, 678 participants, *I*^2^:79.3%) (Supplementary Figure 11). This is due to lack of data on anthropometric indices for these age groups specifically.

#### Dietary Intake

Our systematic review includes 21 studies which reported dietary intake trends amongst school-going children and adolescents aged 5 to 15 years (19–22, 24, 25, 30–32, 36, 40–42, 46, 53, 55, 57–59, 67, 68). The tools used to assess dietary intake patterns are presented in Table 1.

The recommended percentage of daily energy contribution, according to the Acceptable Macrnonutrient Distribution Ranges (AMDR), for carbohydrates, proteins, and fats in children age 4–18 years is 45–65%, 10–30%, and 25–35%, respectively (69). Aziz 2014 reported that children from schools across Pakistan had an overall increased daily intake of carbohydrates (60–75%) (57). Two separate studies conducted in different cities across Pakistan reported the highest carbohydrate consumption amongst children from Balochistan (41, 57). Aziz et al. (30) conducted a study on children from Karachi and reported they have an upper limit of carbohydrate consumption (30).

Aziz et al. (41) reports children generally had the lowest consumption of protein compared to the recommended daily allowance (RDA) (41). Sultana et al. (42) conducted a study on children from Punjab and reported they have the highest protein intake (12%) when compared to other provinces (57). A study assessing lunch box contents amongst 1,360 students noticed meals to be low in proteins and fiber but high in fat (42). Aziz et al. (41) and Aziz and Hosain (57) conducted two studies assessing **fat** intake and it was noted that fat intake amongst children across Pakistan was below the recommended daily standards (41, 57).

For **micronutrients**, Kausar 2018 reported girls to have inadequate dietary intake with their daily consumption being less than the Recommended Daily Allowance (RDA) (22). This was seconded by Zaman et al. (46), reporting female participants to have an overall lower energy intake and failure to meet the recommended intake of vitamins A, C, D, E, folic acid, phosphorus, zinc, sodium, potassium, iron, and magnesium as compared to the RDA (46). Males on the other hand were found to have a higher carbohydrate, sugar, fiber, and fat consumption (46). Children from high socioeconomic status settings were observed to have a higher vitamin and supplements intake (68).

Aziz et al. (55) reported **breakfast consumption** varied with socioeconomic status as children from rural areas or squatter settlements were more likely to skip breakfast. However, Shaukat et al. (40) reported 29% of their population from an urban setting skipped breakfast. A single study reported 8% of their population skipped breakfast and were more likely to be overweight or obese (*p* < 0.002) (31). Qureshi et al. (32), on the other hand, reports 82.2% of their population had insufficient breakfast and found a higher prevalence of thinness and stunting amongst them.

There are 11 studies included in our systematic review that reported dietary intake in children according to food groups. Table 5 below gives an overview of the dietary intake patterns. It can be noted that children have suboptimal vegetable and fruit intake while consumption of soft drinks and sweets/chocolates is high.

**Table 5:** Dietary intake frequency.

**References**	**Poultry and meat**	**Dairy and milk products**	**Eggs**	**Fruits**	**Vegetables**	**Snacks**	**Sweets and chocolates**	**Grains and cereal**	**Beans and pulses**	**Soft drinks**	**Junk food**
Anwar et al. (19)	20.1% (5–7 times/wk)	48.5% (5–7 times/wk)		53.2% (5–7 times/wk)	21.8% (5–7 times/wk)	31.4% (5–7 times/wk)	23.5% (5–7 times/wk)	50.5% (5–7 times/wk)	3.1% (5–7 times/wk)		
Irshad et al. (61)	100% (occasionally)	13.75% (2 times/day) 50% (1 times/wk)	13.75 (1 time/day) 61.25% (1 times/ wk); 25% (occasionally)	100% (1x/mo)	13.75% (1 times/ day) 86.25% (2 times/day)				Pulses: 0% (1 times/ day); 90% (2 times/day)		
Afzal et al. (53)		Milk: 47.2% (1–3 times/wk) Milk:28.8% (daily) Yogurt: 51.5% (daily) Cheese: 44.65% (1–3 times/wk)		54% 1–3 days/wk	52.55% 1–3 times/wk					51.5% 4–6 days/wk	
Aziz et al. (55)	8.4% 6–7 days/wk	Milk and alternatives: 42.1% 6–7 days/wk	15.6% (6–7 days/wk) 59% (0–2 days/wk)	16.7% (6–7 days/wk) 50.5% (0–2 days/wk)	26.2% (6–7 days/ wk); 21.6% (0–2 days/wk)				Lentils and pulses: 7.5% (6–7 days/ week) 51.4% (0–2 days/wk)		64.7% (6–7 days/wk) 9.2% (0–2 days/wk)
Aziz et al. (30)				<15%							
Iqbal et al. (68)		Milk: 57% (everyday) Yogurt: 35.8% (everyday) Cheese:13.2% everyday		38% (at least 1 time/day)	21.1% (everyday)		40.9% (at least/day)				
Ishaque et al. (20)				16.7% (>3x/day) 6.03% do not eat fruits at all	10.4% (>3x/day) 14.2% do not eat vegetables.					13.2% (everyday)	
Jafar et al. (21)				19.8% did not consume fruits and vegetables at all	19.8% did not consume fruits and vegetables at all		71.2% (at least 1x/day)				
Qureshi et al. (32)		34.6% did not drink milk 65.4% drank milk regularly		13.7% denied eating fruits 86.3% ate fruits regularly							
Shaukat et al. (40)		Milk: 68% (2 glasses/day)		57% (everyday)	57% (everyday)					29% (everyday)	
Sultana et al. (42)										84.2% (everyday) 23.9% (two or more drinks everyday)	

*Wk, week*.

## Discussion

In the present systematic review targeting 62,148 individuals, the limited evidence suggests the presence of DBM among school-going children and adolescents age 5 to 15 years. Our pooled analyses have found that approximately one-quarter of these children are underweight (25.1%), stunted (23%), wasted (24%); while 12.5% have thinness, 11.4% are overweight and 6.9% are obese. Dietary intake patterns in school-going children and adolescents aged 5–15 years show relatively high carbohydrate intake and low intake of protein-rich foods, compared to RDA, with suboptimal consumption of fruits and vegetables and increased intake of soft drinks and sweets/chocolates.

In the 1990s, using data on children <5 years of age, Pakistan was only dealing with a high prevalence of undernutrition. However, in the 2010s, this rhetoric changed and Pakistan emerged as a country facing DBM with >30% overweight prevalence (3). A similar transition was noted amongst countries within the lower quartile Gross Domestic Product (GDP) per capita purchasing power parity. This change has been associated with the concept of nutritional transition, which is about changes in the dietary patterns, physical activity, and tendency toward a sedentary lifestyle affecting body composition, fat distribution, and nutritional problems thereby leading to a rapid increase in overweight, obesity, and nutritional related non-communicable diseases (70). Pakistan has also been experiencing this nutritional transition with the rapid urbanization and change in diets. This trend is observed in our systematic review with children reporting an increased intake of carbohydrates, soft drinks, and sweets/chocolates.

The subgroup analysis (Table 4) revealed a higher prevalence of undernutrition (underweight, stunting, and wasting), except thinness, amongst girls, while overweight, obesity, and thinness were higher amongst boys. This disparity highlights the issue of gender inequality which has been embedded in the Pakistani culture, with parents having a strong preference for sons, leading to girls being neglected (54). The National Nutrition Survey (NNS) 2018 of Pakistan, on the other hand, reports higher prevalence of underweight and obesity in adolescent boys and higher overweight prevalence in adolescent girls age 10–19 years (71). 78.4% of the studies were conducted in a school setting and according to Pakistan Annual Report 2016 by UNICEF, 22.6 million children age 5–16 years in Pakistan are out of school (72), hence, more data is needed from communities and rural areas to generalize trends of different anthropometric indices for children across Pakistan (71). Although this is a region-specific finding, even globally there is limited data on school-going children and early adolescents 5–15 years of age (6).

Higher undernutrition prevalence was also noted amongst children attending government schools, children from low socioeconomic backgrounds, and children living in rural areas. This could be attributed to poor living standards and food insecurity coupled with poor dietary practices amongst individuals living in poverty (26). The NNS 2018 survey reports 36.7% households in Pakistan to be facing food insecurity (71). A higher prevalence of overnutrition (overweight and obesity) was noted amongst children attending private schools, children from high socioeconomic backgrounds, and children living in urban areas. This trend is most likely due to the rapid urbanization and change in diet to higher consumption of carbohydrate rich foods, fast foods and carbonated/energy drinks with high sugar content along with a change to a more sedentary lifestyle.

Best 2010 conducted a review to assess the nutritional status of children age 5–12 years from Latin America, Africa, Asia, and the Eastern Mediterranean region and reported high underweight and thinness prevalence in South-East Asia and Africa while overweight prevalence was reported to be below 15% (73). In 2010, East Africa, the Pacific, and sub-Saharan Africa were reported to have a greater overweight prevalence (26.5 and 22.2%, respectively) than that of underweight (7.9 and 12.1%, respectively) (74). A cross-sectional study conducted in Lebanon also reported coexistence of under- and over-nutrition manifested as an overall prevalence of stunting to be 13.7% and overweight to be 7.2% amongst 153 5–14 years (75). On the other hand, a more recent analysis by Caleyachetty 2018 of data from global school-based student health surveys on children age 12–15 years from 57 LMICs and reported an overall 10.2% stunting prevalence, 5.5% thinness, and 21.4% overweight and obesity prevalence (76).

Dietary studies of school-aged children in Pakistan depict relatively high carbohydrate intake and low intake of protein-rich foods, fruits, and vegetables (46, 57, 77, 78). The culture, myths, and misconceptions about dietary habits are different in every region and hence cannot be used to generalize this trend across Pakistan. Two studies have reported the highest carbohydrate intake amongst children from Quetta and Balochistan (41, 55), however, more evidence is needed as not many studies have reported data specifically from these regions. There is a need to develop context-specific behavior change messages for school-aged children to encourage consumption of easily available, accessible, and affordable protein- and vitamin-rich foods such as lentils, seasonal fruits, and vegetables, as well as milk and its derivatives. An increase in consumption of a healthy, balanced diet will also help support the agrarian economy and encourage the utilization of local products to boost immunity and reduce chances of chronic diseases and, therefore, a reduction in the burden on the health sector (79).

Ochola 2014 conducted a systematic review on dietary intake habits of children age 6–12 years from different LMICs. They reported limited diversity and availability of food groups for children and reported children to have a higher intake of plant-based food sources, but an overall low fruit and vegetable intake and limited animal foods, thereby many being deficient in micronutrients. In Kuala Lumpur, 20% of school-going children and adolescents skipped at least one meal a day, with the most commonly skipped meal being breakfast (12.6%) while 32% of adolescents rarely consumed breakfast in Ghana. An increasing trend of processed and fast-food consumption was noted amongst children living in urban areas, with a greater preference for foods high in sugar, salt, and saturated fats. Ochola and Masibo (80) highlighted the need for nutrition education, not only for the school management, children, and parents but also the community at large, to spread awareness and sensitize the people about healthy eating habits (80).

The limitations identified in this review included that (i) studies used different tools and standards, such as the WHO or CDC criteria or did not specify, to categorize anthropometric indices, which led to lack of uniformity and possibility of over-or under-estimation of anthropometric measures, (ii) majority of the studies were conducted in urban setting with most of the data collected from the cities of Lahore and Karachi alone, (iii) majority of the studies had a sample size <500 (*n* = 27), (iv) poor assessment of macro-and micronutrient consumption amongst children and (v) overall poor quality assessment of the included studies with 88.2% studies clearly specified and defined their population, while only 33.3% provided justification for sample size calculation with outcomes defined by 74.5% of the studies, no study had outcomes blinded to assessors and only 25.5% of the studies measured confounding variables and adjusted them statistically to assess associations to the outcomes. We could not measure publication bias for this review using SUMARI, as the estimates were proportions. It is recommended that good quality, large-scale cross-sectional surveys should be conducted for this age group especially in LMICs, along with micronutrient assessment as a component of future research for a better understanding of the problems and to help design specific programs to ameliorate the specific needs.

## Conclusion

This systematic review identifies the burden of malnutrition and dietary patterns in school-going children and early adolescents from Pakistan and highlights the gaps that need to be addressed. Large-scale population-representative studies are still required, with standardized tools for anthropometry and dietary assessment. As the prevalence of DBM for school-going children and early adolescents age 5–15 years in other LMICs is not known, similar reviews from each region also need to be conducted. Such reviews will allow epidemiologists to first assess the availability of data in this age group, then identify their malnutrition trends, and thereby allow them to recognize the gaps and formulate interventions that can better tackle the issue of DBM in this age group globally. Notwithstanding, the need for more evidence; the recent review identifies the high burden of both under- nutrition and over- nutrition in this age group and the relevant mult-sectoral stakehlders should a take a note and plan for programs for this specific and very important age goup.

## Data Availability Statement

The original contributions presented in the study are included in the article/Supplementary Material, further inquiries can be directed to the corresponding author/s.

## Author Contributions

DK and JD: formed the search strategy, identified relevant articles, extracted data, and analyzed it. They also conducted a quality assessment for all included studies. ZB and JD: conceptualized and designed this study. ZL: performed the analysis. ZB, JD, and ZL: guided other authors throughout the process. SZ, AS, MR, AD, and AK: reviewed, provided critical inputs, and revised the manuscript. All authors contributed to the article and approved the submitted version.

## Funding

This systematic review was funded by SCANS consortium including the Trust for Vaccines & Immunizations (Pakistan) and the Aga Khan University (Karachi, Pakistan). The authors declare that this study also received funding from Mother & Child Care & Research Inc. The funder was not involved in the study design, collection, analysis, interpretation of data, the writing of this article or the decision to submit it for publication.

## Conflict of Interest

The authors declare that the research was conducted in the absence of any commercial or financial relationships that could be construed as a potential conflict of interest.

## Publisher's Note

All claims expressed in this article are solely those of the authors and do not necessarily represent those of their affiliated organizations, or those of the publisher, the editors and the reviewers. Any product that may be evaluated in this article, or claim that may be made by its manufacturer, is not guaranteed or endorsed by the publisher.

## Abbreviations

DBM: Double Burden of Malnutrition
LMIC: Low- and middle-income country
NWFP: North West Frontier Province
RDA: Recommended Daily Allowance.
