# Supplementary material for: Nutritional Status and Dietary Intake of School-Age Children and Early Adolescents: Systematic Review in a Developing Country and Lessons for the Global Perspective
Source: Front Nutr. 2022 Feb 2;8:739447. doi: 10.3389/fnut.2021.739447 (PMC8848764; doi:10.3389/fnut.2021.739447)

**Supplementary Table 1a Search Strategy PubMed**

("Nutritional Status"[Mesh] OR "Nutrition Policy"[Mesh] OR "Nutrition Assessment"[Mesh] OR "Nutrition Surveys"[Mesh] OR "Diet, Food, and Nutrition"[Mesh] OR "Diet"[Mesh] OR "Micronutrients"[Mesh] OR "Exercise"[Majr:noexp] OR "Anthropometry"[Mesh] OR "Body Mass Index"[Mesh] OR "Feeding Behavior"[Majr:noexp] OR "Adolescent Nutritional Physiological Phenomena"[Mesh] OR "Child Nutritional Physiological Phenomena"[Mesh] OR "child nutrition sciences"[Mesh] OR "Health Behavior"[Mesh] OR "Malnutrition"[Mesh] OR "Diet Records"[Mesh] OR "Energy Intake"[Mesh] OR "nutrition"[All Fields]) AND ("Schools"[Mesh] OR "child"[MeSH Terms:noexp] OR "Child/education"[Mesh:noexp] OR "Child/epidemiology"[Mesh:noexp] OR "Child/growth and development"[Mesh:noexp] OR "Child/statistics and numerical data"[Mesh:noexp] OR "Adolescent"[Mesh] OR "teenager*"[All Fields] OR "children"[All Fields]) AND ("Pakistan"[Mesh] OR "South Asia"[All Fields] OR "developing countries*"[All Fields] OR "third world countries"[All Fields] OR "Pakistan"[All Fields])

**Table 1b Search Strategy Medline**

| **#** | **Searches** | **Results** |
| --- | --- | --- |
| 1 | exp *"diet, food, and nutrition"/ or *food/ or *nutritional physiological phenomena/ or *child nutritional physiological phenomena/ or exp diet/ or *feeding behavior/ or exp nutritional requirements/ or nutritional status/ or *nutritive value/ | 1084727 |
| 2 | exp health policy/ or exp nutrition policy/ or exp recommended dietary allowances/ or *politics/ or *private sector/ or *public sector/ | 120045 |
| 3 | *health impact assessment/ or *nutrition assessment/ or *records as topic/ or *registries/ or *"surveys and questionnaires"/ or *vital statistics/ | 74670 |
| 4 | exp "surveys and questionnaires"/ or *health care surveys/ or *health surveys/ or *nutrition surveys/ or *diet surveys/ or *patient health questionnaire/ or *vital statistics/ | 907251 |
| 5 | *Micronutrients/df, st, sn [Deficiency, Standards, Statistics & Numerical Data] | 446 |
| 6 | *anthropometry/ or *body fat distribution/ or *body mass index/ or *body size/ or *body surface area/ or *waist-hip ratio/ | 31366 |
| 7 | *exercise/ or *physical fitness/ | 76432 |
| 8 | *child nutritional physiological phenomena/ or *adolescent nutritional physiological phenomena/ or exp diet/ or *eating/ or *feeding behavior/ or exp nutritional requirements/ or exp *nutritional status/ or *nutritive value/ or *"growth and development"/ | 323408 |
| 9 | *malnutrition/ or *severe acute malnutrition/ or *starvation/ or *overnutrition/ | 13433 |
| 10 | exp diet records/ or exp *medical records/ or *registries/ | 101494 |
| 11 | *child nutritional physiological phenomena/ or *diet/ or exp energy intake/ or exp healthy diet/ or *feeding behavior/ or *nutritional requirements/ or *nutritional status/ or *nutritive value/ | 161911 |
| 12 | exp adolescent/ or exp child/ | 2792765 |
| 13 | Adolescent/gd [Growth & Development] | 1 |
| 14 | exp asia, south/ or exp pakistan/ | 98214 |
| 15 | "teenager".mp. [mp=ti, ab, tx, ct, sh, ot, nm, hw, fx, kf, px, rx, an, ui, sy] | 12923 |
| 16 | "child".mp. [mp=ti, ab, tx, ct, sh, ot, nm, hw, fx, kf, px, rx, an, ui, sy] | 2598325 |
| 17 | "5-15 years".mp. [mp=ti, ab, tx, ct, sh, ot, nm, hw, fx, kf, px, rx, an, ui, sy] | 4634 |
| 18 | "weight for height".mp. [mp=ti, ab, tx, ct, sh, ot, nm, hw, fx, kf, px, rx, an, ui, sy] | 80925 |
| 19 | "weight for age".mp. [mp=ti, ab, tx, ct, sh, ot, nm, hw, fx, kf, px, rx, an, ui, sy] | 27213 |
| 20 | "height for age".mp. [mp=ti, ab, tx, ct, sh, ot, nm, hw, fx, kf, px, rx, an, ui, sy] | 14272 |
| 21 | "pakistan".mp. [mp=ti, ab, tx, ct, sh, ot, nm, hw, fx, kf, px, rx, an, ui, sy] | 39940 |
| 22 | 1 or 2 or 3 or 4 or 5 or 6 or 7 or 8 or 9 or 10 or 11 or 18 or 19 or 20 | 2327500 |
| 23 | 12 or 13 or 15 or 16 or 17 | 3597742 |
| 24 | 14 or 21 | 123549 |
| 25 | 22 and 23 and 24 | 7880 |

**Table 2 PRISMA Checklist**

| **Section/topic** | **#** | **Checklist item** | **Reported on page #** |
| --- | --- | --- | --- |
| **TITLE** | | |  |
| Title | 1 | Identify the report as a systematic review, meta-analysis, or both. | 1 |
| **ABSTRACT** | | |  |
| Structured summary | 2 | Provide a structured summary including, as applicable: background; objectives; data sources; study eligibility criteria, participants, and interventions; study appraisal and synthesis methods; results; limitations; conclusions and implications of key findings; systematic review registration number. | 1 |
| **INTRODUCTION** | | |  |
| Rationale | 3 | Describe the rationale for the review in the context of what is already known. | 3 |
| Objectives | 4 | Provide an explicit statement of questions being addressed with reference to participants, interventions, comparisons, outcomes, and study design (PICOS). | 3 |
| **METHODS** | | |  |
| Protocol and registration | 5 | Indicate if a review protocol exists, if and where it can be accessed (e.g., Web address), and, if available, provide registration information including registration number. | 4 |
| Eligibility criteria | 6 | Specify study characteristics (e.g., PICOS, length of follow-up) and report characteristics (e.g., years considered, language, publication status) used as criteria for eligibility, giving rationale. | 4 |
| Information sources | 7 | Describe all information sources (e.g., databases with dates of coverage, contact with study authors to identify additional studies) in the search and date last searched. | 4 |
| Search | 8 | Present full electronic search strategy for at least one database, including any limits used, such that it could be repeated. | 4 |
| Study selection | 9 | State the process for selecting studies (i.e., screening, eligibility, included in systematic review, and, if applicable, included in the meta-analysis). | 4 |
| Data collection process | 10 | Describe method of data extraction from reports (e.g., piloted forms, independently, in duplicate) and any processes for obtaining and confirming data from investigators. | 4 |
| Data items | 11 | List and define all variables for which data were sought (e.g., PICOS, funding sources) and any assumptions and simplifications made. | 4 |
| Risk of bias in individual studies | 12 | Describe methods used for assessing risk of bias of individual studies (including specification of whether this was done at the study or outcome level), and how this information is to be used in any data synthesis. | 4 |
| Summary measures | 13 | State the principal summary measures (e.g., risk ratio, difference in means). | 5 |
| Synthesis of results | 14 | Describe the methods of handling data and combining results of studies, if done, including measures of consistency (e.g., I^2^) for each meta-analysis. | 4, 5 |
| Risk of bias across studies | 15 | Specify any assessment of risk of bias that may affect the cumulative evidence (e.g., publication bias, selective reporting within studies). | 4 |
| Additional analyses | 16 | Describe methods of additional analyses (e.g., sensitivity or subgroup analyses, meta-regression), if done, indicating which were pre-specified. | 4, 5 |
| **RESULTS** |  |  |  |
| Study selection | 17 | Give numbers of studies screened, assessed for eligibility, and included in the review, with reasons for exclusions at each stage, ideally with a flow diagram. | 5, 16 |
| Study characteristics | 18 | For each study, present characteristics for which data were extracted (e.g., study size, PICOS, follow-up period) and provide the citations. | 5, 10-12 |
| Risk of bias within studies | 19 | Present data on risk of bias of each study and, if available, any outcome level assessment (see item 12). | 4 |
| Results of individual studies | 20 | For all outcomes considered (benefits or harms), present, for each study: (a) simple summary data for each intervention group (b) effect estimates and confidence intervals, ideally with a forest plot. | 16, 17 |
| Synthesis of results | 21 | Present results of each meta-analysis done, including confidence intervals and measures of consistency. | 5,6,16,17 |
| Risk of bias across studies | 22 | Present results of any assessment of risk of bias across studies (see Item 15). | 13 |
| Additional analysis | 23 | Give results of additional analyses, if done (e.g., sensitivity or subgroup analyses, meta-regression [see Item 16]). | 5, 6 |
| **DISCUSSION** |  |  |  |
| Summary of evidence | 24 | Summarize the main findings including the strength of evidence for each main outcome; consider their relevance to key groups (e.g., healthcare providers, users, and policy makers). | 7 |
| Limitations | 25 | Discuss limitations at study and outcome level (e.g., risk of bias), and at review-level (e.g., incomplete retrieval of identified research, reporting bias). | 7, 8 |
| Conclusions | 26 | Provide a general interpretation of the results in the context of other evidence, and implications for future research. | 8 |
| **FUNDING** |  |  |  |
| Funding | 27 | Describe sources of funding for the systematic review and other support (e.g., supply of data); role of funders for the systematic review. | 1 |

**Supplementary Table 3 Characteristics of included studies**

| **#** | **Author Year** | **Study Design** | **City** | **Target Population** | **Setting** | | | **Sample size** | **Anthropometric indices** | | | | | **Associations** |
| --- | --- | --- | --- | --- | --- | --- | --- | --- | --- | --- | --- | --- | --- | --- |
|  |  |  |  |  | **U/R** | **S/C** | **P/G** |  | **thinness** | **stunting** | **Under-weight** | **Over-weight** | **obese** |  |
| 1 | Ahmed, J 2013 | Cross-sectional | Hyderabad | 9-17  Class 6 to 10 | U | S | Both | 501 |  |  |  | 8 | 12 | -Underweight children excluded  -Obesity greater in boys compared to girls  -Children who ate at fat food outlets rather than home were significantly more likely to be obese (p=0.007)  -Children >12y were more protected against obesity than the younger age group  -Children from upper SES were less likely to to be obese compared to lower SES  -Children who rated themselves poor in athletic ability were 5.50 times (p<0.001) more likely to be obese  -Those who ate fruits >= 4 times per week were less likely to be obese ( p=0.02) |
| 2 | Afzal, N. 2017 | Cross-sectional | Okara | 13-18  Girls only | Both | S | Both | 850 | x | x | x | x | x | -Private school girls were significantly taller and heavier than government schoolgirls  -Private school adolescents had greater consumption of fast food |
| 3 | Akbar, F.N. 2015 | Cross-sectional | NA | 6-11 | X | S | X | 150 |  |  | 45.5 | 11 | 2.46 | -Food consumption reported which was assessed using FFQ  -BMI of school children increased with age due to high consumption of fatty and vendor foods and lunchbox avoiding behavior |
| 4 | Anwar, A 2010 | Cross-sectional | Lahore | 10-14  Class 6-7 | U | S | P | 293 |  |  |  | 21.8 | 11.9 | -74.3% obese children watched TV for 1-2 hours  -48.6% obese children did not participate in field sports, 34.3% had less than 3 hours participation  -71.4% didn't attend gym or any other physical training  -21.5% mentioned their parents' attitude towards outdoor activities was discouraging  -22.9% parents of obese children never advise them against 'eating junk food' |
| 5 | Anwer, I 2003 | Cross-sectional | Faisalabad | 6-12 | Both | S | Both | 2,042 | Wasting: 32.9 | 36.1 | 45.3 |  |  | -Children from poor and middle-income SES  -Nutritional status among urban school going children was better  -Higher proportion of stunted, underweight and wasted children are female. |
| 6 | Aziz, A. 2018 | Cross-sectional | Dadu Jacobabad  Kambar  Karachi Kashmore Khairpur  Larkana  Shahdadkot Sukkur | 7-11 | Both | S | G | 1109 |  |  |  |  |  | -Skipping breakfast and snacks was related to low SES and rural residence  -Urban residence and better SES had a positive impact on a child’s frequency and variety of daily protein and fruit intake  -Junk food was consumed more by children living in urban areas and from better SES strata |
| 7 | Aziz, S. 2009 | Cross-sectional | Karachi | 6-17 | U | S | P | 398 |  |  |  | 19.35 | 6 | -46% of obese children consumed more than the upper limit of fat in their diet  -29% of obese children consumed more than the upper limit of carbohydrates in their diet  -Per day carbohydrate, protein and fat consumption by obese children was 57.9%, 12.6% and 35.4% -respectively  -Per day carbohydrate, protein and fat consumption by overweight children was 56.8%, 12.5% and 35.5% respectively  -Less than 15% consumed fruits daily  -85% did not have physical activity as part of their daily routine |
| 8 | Aziz, S. 2009 | Cross-sectional | Karachi  Lahore  Quetta | 6-18 | U | S | P | 652 |  |  |  |  |  | -Children from Karachi consumed more junk food compared to Lahore and Quetta  -Fat intake amongst children across Pakistan is below normal recommended standards  -Carbohydrate consumption was adequate amongst children across Pakistan  -BMI highest amongst boys from Quetta compared to Karachi and Lahore |
| 9 | Aziz, S. 2012 | Cross-sectional | Cities across Pakistan | 3-16 | Both | S | Both | 12,837 |  | 14 |  |  | 5.1 | -Stunting more prevalent in boys as compared to girls |
| 10 | Aziz, S 2014 | Cross-sectional | Cities across Pakistan | 6-16 | Both | C | X | 11,237 |  |  |  |  |  | -High consumption of CHO amongst children across Pakistan  -Children in Pakistan consume deficient amounts of protein and fat in their daily diets  -Lowest protein and highest CHO intake was by children of Balochistan  -Highest protein intake was by children of Punjab  -Fat intake was highest in Khyber Pakhtoonkhwa |
| 11 | Babar, N 2010 | Cross-sectional | Lahore | 6-11 | U | S | Both | 161 |  |  | 29.8 | 13.7 | 8.1 | -41% children with BMI < 5^th^ percentile were from lower SES compared to 19.28% in upper SES  -Malnutrition prevalence was higher amongst children of illiterate mothers compared to literate mothers |
| 12 | Basit, A 2005 | Cross-sectional | Karachi | 8-10 | U | S | X | 92 |  | 2 | 12 |  | 5 | -No significant difference in height, weight, and BMI characteristics amongst boys and girl |
| 13 | Batool, S 2012 | Cross-sectional | Faisalabad | 4-12 | U | S | P | 432 |  | 45.8 | 25.4 |  |  | -4-6y: more girls were stunted compared to boys  -7-12y: More boys stunted compared to girls |
| 14 | Fatima, F 2014 | Cross-sectional | Islamabad | 11-19 | U | C | X | 150 |  |  | 36.3 | 7.1 | 12.1 | - There was a significant association of the intake of meat with the haemoglobin level |
| 15 | Hall, A 2010 | Cross-sectional | NWFP | 5-14 | R | C | X | 2032 | 12 | 43 | 34 |  |  | -Earthquake affected children  -Boys were found to be heavier and taller than girls on average. However, there was no difference between the sexes in their anthropometric indices |
| 16 | Haq, I 2010 | Cross-sectional | Hazara Division | 5-14 | R | S | Both | 3200 |  |  |  |  | 4.78 | -Obesity higher amongst private school going children compared to those going to government school. |
| 17 | Hayyat, M 2019 | Cross-sectional | Lahore | 5-12 | U | S | Both | 240 |  |  | 1.3 | 24.6 | 50 | -Cheaper fast-food cost leads to more fast-food consumption =Fast food consumption more in friends' company compared to family |
| 18 | Iqbal, M 2020 | Cross-sectional | Karachi  Thatta  Hyderabad | 5-14 | X | C | X | 634 | Wasted 30 | 15.5 |  |  |  | -Conducted on child labourers  -Stunting was highest amongst children working in agriculture  -Wasting highest amongst migrant workers  -Stunting significantly more common in girls (21%) compared to boys (9.6%) |
| 19 | Iqbal, T.A. 2017 | Cross-sectional | Islamabad  Rawalpindi | 11-16 | U | S | Both | 332 |  |  |  |  |  | -Incidence of taking vitamins and supplements was found to be more in respondents from high SES  -Incidence of eating lunch brought from home at schools was more amongst respondents from high SES  -Frequency of sweet and dessert consumption was high amongst children from low SES -Children from low SES had a lower vegetable consumption compared to those from middle and high SES  -Fast-food intake was significantly higher in respondents from high SES  -Dairy consumption was found to be higher in respondents from high SES |
| 20 | Irshad, R 2019 | Cross-sectional | Kohistan | 0-14 | X | C | X | 80 | 3.75 | 3.75 | 26.25 |  |  | -Anthropometric findings amongst children from Paras valley are consistent with findings from other backward areas across Pakistan |
| 21 | Ishaque,A. 2012 | Cross-sectional | Karachi | 13-16 | U | S | Both | 431 |  |  |  |  |  | -28% of the students had weight greater than the 85^th^ centile corresponding to overweight and obesity  -There was a higher prevalence of overweight and obesity in boys as compared to girls  -Eating junk food and energy dense food was associated with overweight and obesity  -Positive association was established between students not regularly eating breakfast with overweight and obesity |
| 22 | Jafar, T.H. 2007 | Cross-sectional | Karachi | 5-14 | U | C | X | 1675 |  | 14.6 | 27.9 |  |  | -No child ate the recommended 4-5 daily servings of fruits and vegetables  -19.8% children reported not eating fruits and vegetables at all  -71.2% ate chocolates and sweets at least once a day  -Boys were found to be twice more active compared to girls  -Over 20% children did no exercise either in or out of school |
| 23 | Kauser, T. 2018 | Cross-sectional | Sargodha | 12-15 | U | X | X | 200 |  |  | 53.5 |  |  | -Majority of the girls belonged to low SES, had insufficient diet and poor diet quality  -Majority of the girls had inadequate dietary intake and consume less than the RDA  -65.5% consumed breakfast daily  -4.5% reported never consuming breakfast |
| 24 | Khan, K 2016 | Cross-sectional | Karachi | 7-12 | U | S | Both | 240 |  | 26.3 | 34.9 |  | 0.8 | -Prevalence of underweight was higher in children from govt schools compared to private school going children. |
| 25 | Khan, S 2019 | Cross-sectional | Multan | 3-18 | U | S | Both | 1872 |  |  |  | 10 | 5 | -Children skipping breakfast were significantly more likely to become overweight and obese  -Children who consumed fast-food one to two times per week directly associated with overweight and obesity  -9.3% children physically inactive  -27.7% did physical activity less than 2 times/week -63% were physically more active had a negative association with overweight and obesity  -Use of TV, computer, video games were highly associated with overweight and obesity.  -The chi-square test revealed that family size, no, of siblings, mother’s working status, skipping breakfast, fast food consumption, physical activity and sedentary lifestyle were significantly (P value < 0.05) linked with overweight and obesity. -Prevalence of obesity amongst females was higher compared to males due to skipping breakfast and high consumption of fast foods**.** |
| 26 | Khan, Z 2015 | Cross-sectional | Quetta | 11-16  (Mean age 14; grade 6 to 9) | U | S | P | 423 |  |  |  |  |  | -61% ate breakfast  -54% brought lunch from school canteen |
| 27 | Khuwaja, S 2005 | Cross-sectional | Thatta  Badin  Mirpur Khas  Tharparkar | 6-12 | R | S | G | 1915 |  | 16.5 |  |  |  | -Stunting higher in girls compared to boys, they were 1.26 times more likely to be stunted compared to boys.  -Stunted children were significantly more likely to be female, more than 7 years of age, have a father working as a labourer/farmer, government employee or a shopkeeper. |
| 28 | Marwat, Z 2019 | Cross-sectional | Abbottabad | 7-13 | U | S | Both | 200 |  |  |  |  |  | -Poor grades in underweight children  -Private school children were more intelligent  -Children of matriculated mothers found to be obese |
| 29 | Mian, R 2002 | Cross-sectional | Islamabad | 5-10 | U | C | X | 200 | Wasting 13 | 35 | 29.5 |  |  | -Severe malnutrition in 15.4% of the children -Prevalence of malnutrition was significantly higher among older children. -No association between nutritional status and gender -Linear trend between nutritional status and monthly family income (p<0.03) |
| 30 | Mohsin, S 2017 | Cross-sectional | Nowshera | 6-14 | R | S | G | 163 |  |  | 39.8 |  |  | -23.3% moderately underweight  -16.5% severely underweight |
| 31 | Mushtaq, M 2011 | Cross-sectional | Lahore | 5-12 | U | S | Both | 1860 |  |  |  | 17 | 7.5 | -Higher prevalence of obesity amongst boys than girls (p=0.028)  -Children living in urban areas **with** high SES were significantly at risk for being overweight and obese (p<0.001) |
| 32 | Mushtaq, M 2011 | Cross-sectional | Lahore | 5-12 | U | S | Both | 1860 | 10 | 8 |  |  |  | -Prevalence of stunting significantly increased with age (p<0.001)  -Thinness showed significant increasing trend with age among boys only (p<0.034)  -Significant correlates of thinness include rural area, urban area with low SES, low-income neighbourhoods, low parental education, more siblings, crowded housing and smoking in living place (all <0.001) |
| 33 | Mushtaq, M 2011 | Cross-sectional | Lahore | 5-12 | U | S | Both | 1860 |  |  |  |  |  | -8% skip breakfast  -43% ate fast food at least once a week  -Skipping breakfast, eating fast food and sedentary lifestyle were independent predictors of overweight. |
| 34 | Mustafa, M 2017 | Cross-sectional | Lasbela | 4-15 | R | S | X | 6363 |  |  |  | 5.8 | 3.3 | -Mild malnutrition: 22.6% -Moderate malnutrition: 15.1% -Severe acute malnutrition: 9.2% |
| 35 | Ponum, M 2020 | Cross-sectional | Multan | 4-18 | Both | C | X | 1420 |  | 24.93 |  |  |  | -Stunting greatest in children age 8-11 years  -Boys were more likely to be stunted compared to girl  -Children from rural setting were more likely to be stunted compared to children from urban setting  -More stunted children skipped breakfast than non-stunted children |
| 36 | Qureshi, M 2017 | Cross-sectional | Hyderabad | 5-18 | U | S | G | 422 | 13.7 |  | 18.3 |  |  | -18.3% children age 5-10 were underweight  -13.7% children age 5 to >12 years were thin  -Stunting was significantly associated with consuming insufficient breakfast (only consuming rusk/biscuit, roti/bread and tea)  -82.2% had insufficient breakfast consumption  -13.7% denied eating fruits  -86.3% ate fruits regularly  -34.6% did not drink milk  -65.4% drank milk regularly |
| 37 | Rahman, A 2013 | Cross-sectional | N/A | 12-16 | U | S | G | 661 |  |  |  | 7.7 | 1 | -Hypertension in children is strongly correlated with obesity, asymptomatic proteinuria and hematuria. |
| 38 | Ramzan, M 2010 | Cross-sectional | Dera Ismail Khan | 6-11 | U | S | Both | 322 |  |  | 5.59 |  |  | -Children assessed were affluent schools  -No significant difference in stunting amongst boys and girls was noted |
| 39 | Rehman, Z 2013 | Cross-sectional | Peshawar | 4-12 | U | S | X | 400 |  |  | 30 | 7 | 0 | - The prevalence of malnutrition was more (37%) among four to twelve years school going children |
| 40 | Riaz, R 2010 | Cross-sectional | N/A | 5-10 | X | S | Both | 344 |  | 10.7 | 38.7 | 0.3 |  | -School category i.e., public / private was found to have insignificant association with BMI-for-age (p>0.05).  -Association of school category with rest of the two dependent variables i.e., weight-for-age and stature-for-age was found to be highly significant -The difference in height between public and private school children was insignificant ( p> 0.05), but difference in the mean weight of public (21.3kg±0.61) and private school students (23.1k±0.58) was found to be significant both apparently and statistically (p<0.05). -More boys had normal weight-for-age and BMI-for-age but showed insignificant association with stature-for-age (p>0.05). |
| 41 | Rizwan, A 2010 | Cross-sectional | Karachi | 11-17 | U | S | P | 339 |  |  |  |  |  | odds of being overweight and obese was greater in children who consumed:   - calorie-rich snacks, sugar-sweetened beverages, - visited fast food restaurants more than twice weekly - performed less versus greater than 30 min of activity per physical education class - engaged in sedentary activities for more versus less than 1 h/day - snacked more versus less than twice per week during television watching |
| 42 | Sadiq, S 2019 | Cross-sectional | Karachi | 7-18 | U | S | Both | 1244 |  |  | 5.2 | 9.2 | 6.4 | -Majority of the participants reported normal BMI  -There was a higher prevalence of overweight rather than underweight |
| 43 | Shahid, A 2010 | Cross-sectional | Karachi | 8-14 | U | S | Both | 500 |  |  | 21.4 | 8.2 | 6.6 | -Obesity and overweight more in boys but not statistically significant -Overweight and obese children had higher BP compared to healthy children -9% of obese were hypertensive, 6% prehypertensive -7.3% overweight were hypertensive, 12.2% were prehypertensive -Regarding ethnic difference in levels of systolic blood pressure and diastolic blood pressure among the various ethnic groups residing in Karachi, mean systolic blood pressure and diastolic blood pressure was in the descending order from Muhajirs-Baluchis-Pushtuns- Sindhis-Punjabis -An increase in BMI and waist circumference was associated with raised blood pressure |
| 44 | Shahid, B 2017 | Cross-sectional | Lahore | 10-16 | U | S | P | 197 |  |  |  |  | 18.2 | -Obese and overweight reported together |
| 45 | Shaukat, F 2013 | Cross-sectional | Lahore | 6-16 | U | S | Both | 370 |  |  | 18.4 | 35.1 |  | -Strong association between high SES to overweight  -Strong association between physical activity >30 min to overweight  -Strong association between intake of carbohydrates to overweight  -Strong association between consumption of more than one beverage daily to overweight |
| 46 | Siddique, S 2013 | Cross-sectional | Abbottabad | 5-10 | X | X | X | 408 |  | 0.7 | 2.98 |  |  | -Indices assessed as a percentage of predicted height-for-age  -Significant association of father's education level with W/A  -Linear correlation of father's occupation to health (W/A) of his child |
| 47 | Sultana, F 2017 | Cross-sectional | Rawalpindi  Islamabad | 10-18 | U | S | X | 1360 |  |  |  | 2.0 | 0.2 | -Obesity in the school children was much lower than reported in western affluent societies  -21.1% played no outdoor games  -84.2% consumed soft drinks daily  -23.9% consumed two or more drinks per day |
| 48 | Warraich, H 2009 | Cross-sectional | Karachi | 11-17 | U | S | Both | 284 |  |  | 52 | 8 | 6 | -Socio-economic factors are important since obesity and overweight increase with SES.  -Higher SES groups should be targeted for overweight while underweight is a problem of lower SES. |
| 49 | Zahid, S 2017 | Cross-sectional | Faisalabad | 5-12 | U | S | Both | 200 | Wasting: 20 | 25.5 | 26.5 | 8.5 | 9.5 | -71.6% of stunted children belonged to government schools  -57.1% of children with wasting belonged to government schools  -95% obese children were from private school  -65% overweight children were from private school  -64% of the 'at the risk of becoming overweight' students belonged to private school.  -More boys obese compared to girls  -More boys stunted and suffered from wasting as opposed to girls |
| 50 | Zainab, S 2016 | Cross-sectional | Karachi | 10-14 | U | C | X | 385 | 8.1  Severe: 1.3 | Mild: 18.7  Moderate: 40.3  Severe: 31.4 |  | 17.9 | 3.6 | -Nutritional status amongst domestic child labourers in Karachi is poor with high prevalence of stunting |
| 51 | Zaman, R 2013 | Cross-sectional | Sialkot | 13-16 | U | S | X | 328 |  |  |  | 18.9 | 3.96 | -Female participants had lower total energy intake and lower energy intake/kg of body weight  -Carbohydrate, sugar, fiber and fat consumption higher amongst males  -Both genders failed to meet estimated average requirements for vitamin A, C, D, E, calcium, potassium and magnesium  -Females also failed to meet the recommended intake of vitamin A, D, E, C, folic acid, phosphorus, zinc, sodium, potassium, iron and magnesium |
| Abbreviations: BMI: Body Mass Index; BP: blood pressure; C: community; CHO: carbohydrates; FFQ: food frequency questionnaire; G: government; gvt: government; P: private; pvt: private; R: rural; RDA: Recommended Daily Allowance; S: school; SES: socio-economic status; U: urban | | | | | | | | | | | | | | |

**Supplementary Table 4 Quality Assessment of included studies**

| **Study ID** | **1. Was the research question or objective in this paper clearly stated?** | **2. Was the study population clearly specified and defined?** | **3. Was the participation rate of eligible persons at least 50%?** | **4. Were all the subjects selected or recruited from the same or similar populations (including the same time period)? Were inclusion and exclusion criteria for being in the study prespecified and applied uniformly to all participants?** | **5. Was a sample size justification, power description, or variance and effect estimates provided?** | **6. For the analyses in this paper, were the exposure(s) of interest measured prior to the outcome(s) being measured?** | **7. Was the timeframe sufficient so that one could reasonably expect to see an association between exposure and outcome if it existed?** | **8. For exposures that can vary in amount or level, did the study examine different levels of the exposure as related to the outcome (e.g., categories of exposure, or exposure measured as continuous variable)?** | **9. Were the exposure measures (independent variables) clearly defined, valid, reliable, and implemented consistently across all study participants?** | **10. Was the exposure(s) assessed more than once over time?** | **11. Were the outcome measures (dependent variables) clearly defined, valid, reliable, and implemented consistently across all study participants?** | **12. Were the outcome assessors blinded to the exposure status of participants?** | **13. Was loss to follow-up after baseline 20% or less?** | **14. Were key potential confounding variables measured and adjusted statistically for their impact on the relationship between exposure(s) and outcome(s)?** |
| --- | --- | --- | --- | --- | --- | --- | --- | --- | --- | --- | --- | --- | --- | --- |
| Ahmed, J 2013 | Yes | Yes | Yes | Yes | Yes | No | No | Yes | Yes | No | Yes | No | No | Yes |
| Afzal, N. 2017 | Yes | Yes | Yes | Yes | No | No | No | Yes | No | No | No | No | No | No |
| Akbar, F.N. 2015 | Yes | Yes | Yes | Yes | No | No | No | No | No | No | No | No | No | Yes |
| Anwar, A 2010 | Yes | Yes | Yes | Yes | No | No | No | No | No | No | Yes | No | No | No |
| Anwer, I 2003 | Yes | No | Yes | Yes | No | No | No | No | No | No | Yes | No | No | No |
| Aziz, A 2018 | Yes | No | Yes | Yes | No | No | No | No | No | No | No | No | No | No |
| Aziz, S. 2009 | Yes | Yes | Yes | Yes | No | No | No | No | No | No | Yes | No | No | No |
| Aziz, S. 2009 | Yes | Yes | Yes | Yes | No | No | No | No | No | No | No | No | No | No |
| Aziz, S 2012 | Yes | Yes | Yes | No | No | No | No | No | No | No | Yes | No | No | No |
| Aziz, S. 2014 | Yes | Yes | Yes | Yes | No | No | No | No | No | No | Yes | No | No | No |
| Babar, N 2010 | Yes | No | Yes | Yes | No | No | No | No | No | No | No | No | No | No |
| Basit, A 2005 | Yes | Yes | Yes | Yes | No | No | No | No | No | No | Yes | No | No | No |
| Batool, S 2012 | Yes | Yes | Yes | Yes | No | No | No | No | No | No | Yes | No | No | No |
| Fatima, F 2014 | Yes | Yes | Yes | Yes | Yes | No | No | No | No | No | Yes | No | No | No |
| Hall, A 2010 | Yes | Yes | Yes | Yes | Yes | No | No | No | No | No | Yes | No | No | No |
| Haq, I 2017 | Yes | Yes | Yes | Yes | No | No | No | No | No | No | Yes | No | No | Yes |
| Hayyat, M 2019 | Yes | Yes | Yes | Yes | No | No | No | No | No | No | No | No | No | No |
| Iqbal, M 2020 | Yes | Yes | Yes | Yes | Yes | No | No | No | No | No | Yes | No | No | No |
| Iqbal, T.A. 2017 | Yes | Yes | Yes | Yes | Yes | No | No | No | No | No | No | No | No | No |
| Irshad, R 2019 | Yes | Yes | Yes | Yes | No | No | No | No | No | No | Yes | No | No | No |
| Ishaque,A. 2012 | Yes | Yes | Yes | Yes | Yes | No | No | No | No | No | Yes | No | No | No |
| Jafar, T.H. 2007 | Yes | Yes | Yes | Yes | No | No | No | No | No | No | Yes | No | No | Yes |
| Kauser, T. 2018 | Yes | Yes | Yes | Yes | Yes | No | No | No | No | No | No | No | No | No |
| Khan, K 2016 | Yes | Yes | Yes | Yes | No | No | No | No | No | No | Yes | No | No | No |
| Khan, S 2019 | Yes | Yes | Yes | Yes | No | No | No | No | No | No | Yes | No | No | Yes |
| Khan, Z 2015 | Yes | Yes | Yes | Yes | Yes | No | No | No | No | No | No | No | No | No |
| Khuwaja, S 2005 | Yes | Yes | Yes | Yes | No | No | No | No | Yes | No | Yes | No | No | Yes |
| Marwat, Z 2019 | Yes | Yes | Yes | Yes | No | No | No | No | Yes | No | No | No | No | No |
| Mian, R 2002 | Yes | Yes | Yes | Yes | No | No | No | No | No | No | Yes | No | No | No |
| Mohsin, S 2017 | Yes | Yes | Yes | Yes | No | No | No | No | No | No | Yes | No | No | Yes |
| Mushtaq, M 2011 | Yes | Yes | Yes | Yes | Yes | No | No | Yes | Yes | No | Yes | No | No | Yes |
| Mushtaq, M 2011 | Yes | Yes | Yes | Yes | Yes | No | No | Yes | Yes | No | Yes | No | No | Yes |
| Mushtaq, M 2011 | Yes | Yes | Yes | Yes | Yes | No | No | Yes | Yes | No | Yes | No | No | Yes |
| Mustafa, M 2017 | Yes | No | Yes | Yes | No | No | No | No | No | No | No | No | No | No |
| Ponum, M 2020 | Yes | Yes | Yes | Yes | No | No | No | Yes y | No | No | No | No | No | No |
| Qureshi, M 2017 | Yes | Yes | Yes | Yes | No | No | No | No | No | No | Yes | No | No | No |
| Rahman, A 2013 | Yes | No | Yes | Yes | No | No | No | No | No | No | Yes | No | No | Yes |
| Ramzan, M 2010 | Yes | No | Yes | Yes | No | No | No | No | Yes | No | Yes | No | No | No |
| Rehman, Z 2013 | Yes | Yes | Yes | Yes | Yes | No | No | No | No | No | Yes | No | No | No |
| Riaz, R 2010 | Yes | Yes | Yes | Yes | Yes | No | No | No | No | No | Yes | No | No | No |
| Rizwan, A 2010 | Yes | Yes | Yes | Yes | No | No | No | Yes | Yes | No | No | No | No | Yes |
| Sadiq, S 2019 | Yes | Yes | Yes | Yes | Yes | No | No | No | No | No | Yes | No | No | No |
| Shahid, A 2010 | Yes | Yes | Yes | Yes | No | No | No | No | No | No | Yes | No | No | No |
| Shahid, B 2017 | Yes | Yes | Yes | Yes | No | No | No | No | No | No | Yes | No | No | No |
| Shaukat, F 2013 | Yes | Yes | Yes | Yes | Yes | No | No | No | No | No | Yes | No | No | No |
| Siddique, S 2013 | Yes | Yes | Yes | Yes | No | No | No | No | No | No | Yes | No | No | No |
| Sultana, F 2017 | Yes | Yes | Yes | Yes | No | No | No | No | No | No | Yes | No | No | No |
| Warraich, H 2009 | Yes | Yes | Yes | Yes | Yes | No | No | Yes | Yes | No | Yes | No | No | No |
| Zahid, S 2017 | Yes | Yes | Yes | Yes | No | No | No | No | No | No | Yes | No | No | No |
| Zainab, S 2016 | Yes | Yes | Yes | Yes | Yes | No | No | No | No | No | Yes | No | No | Yes |
| Zaman, R 2013 | Yes | Yes | Yes | Yes | No | No | No | No | No | No | Yes | No | No | No |

**Supplementary Figures**

**Fig 1a: underweight pooled prevalence in 0 to 19 years**


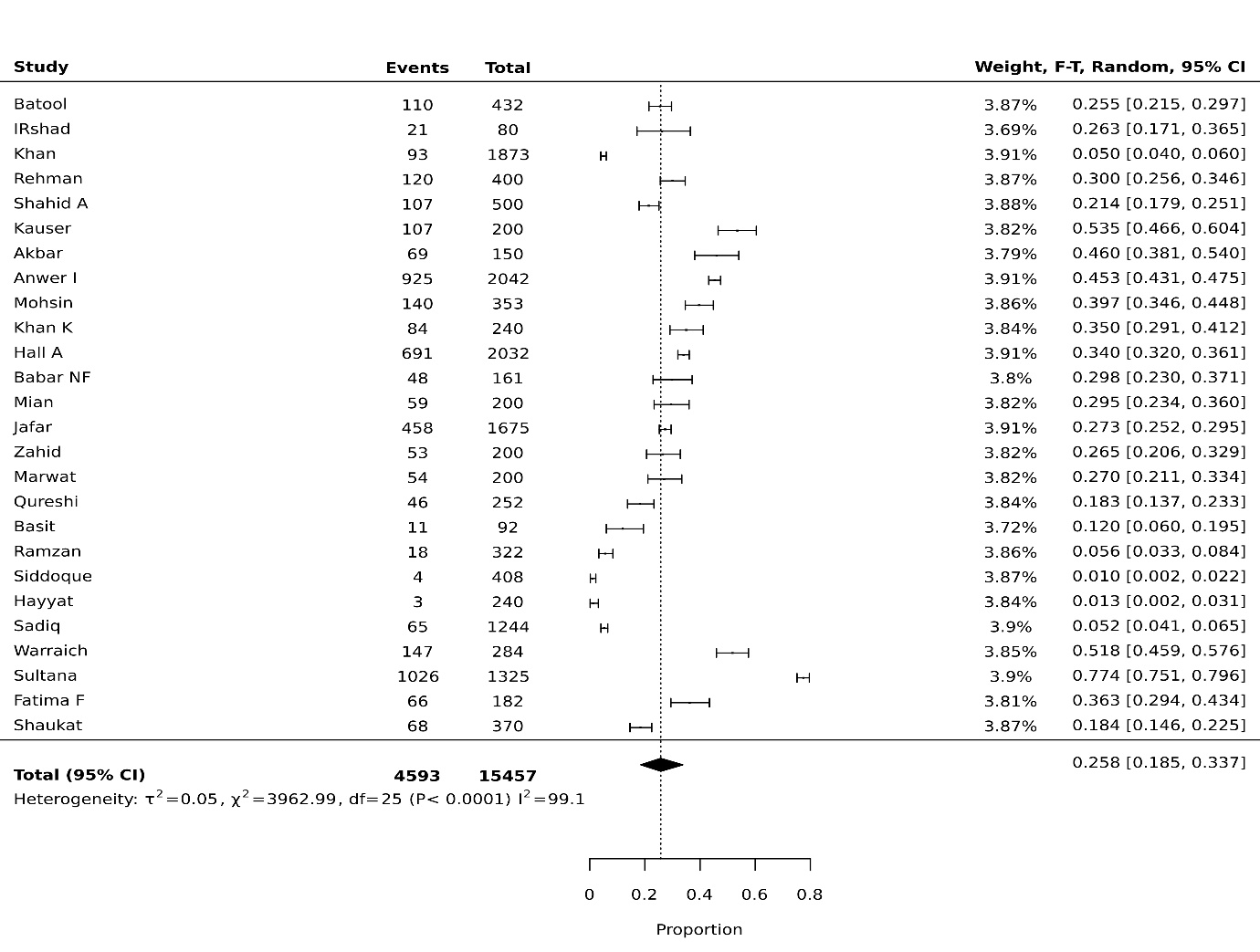


**Fig 1b: stunting pooled prevalence in 0 to 19 years**


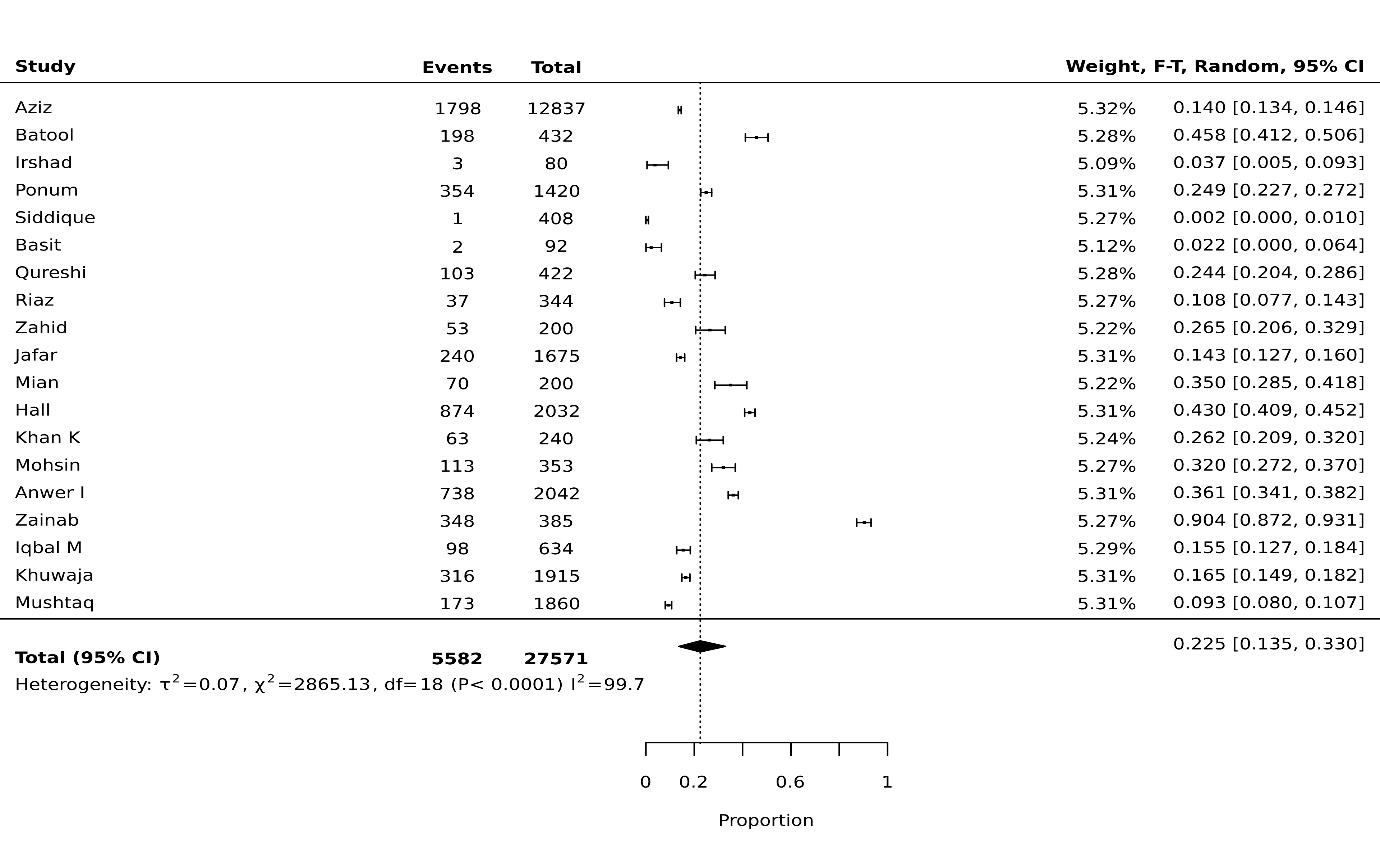


**Fig 1c: wasting pooled prevalence in 0 to 19 years**


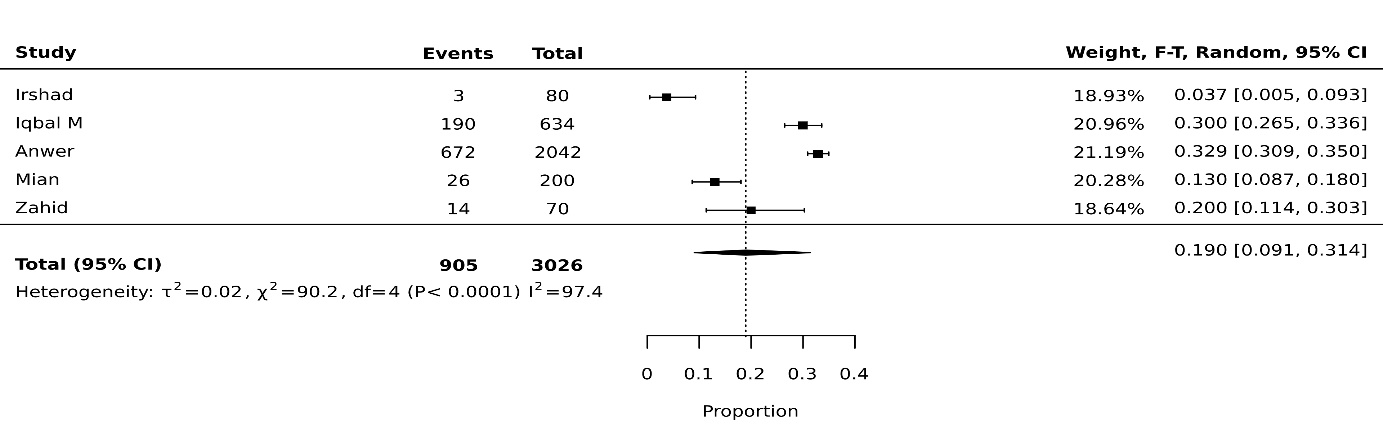
**Fig 1d: thinness pooled prevalence in 0 to 19 years**


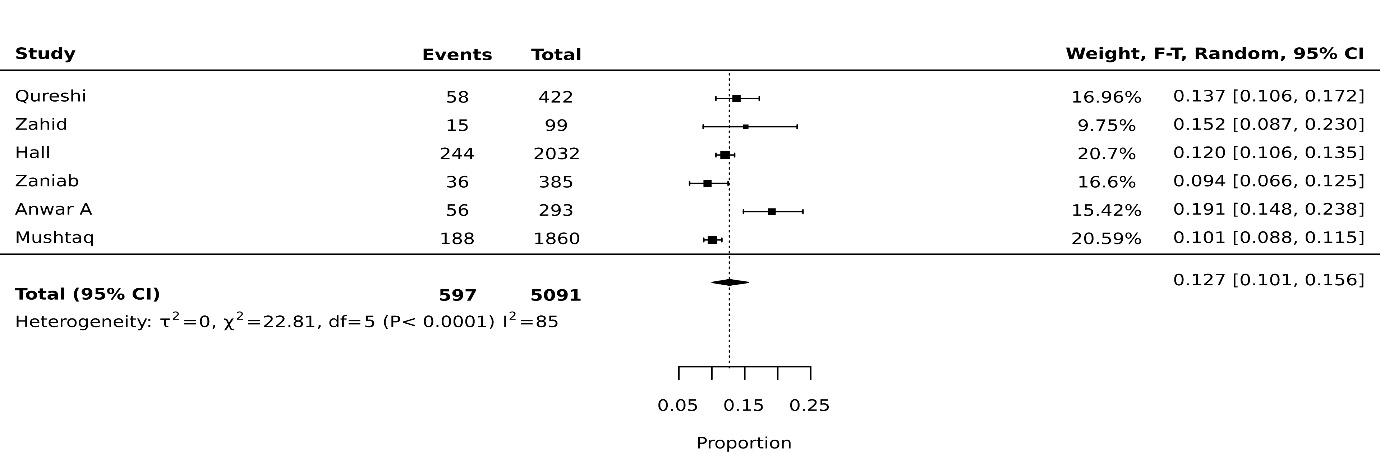
**Fig 1e: overweight pooled prevalence in 0 to 19 years**


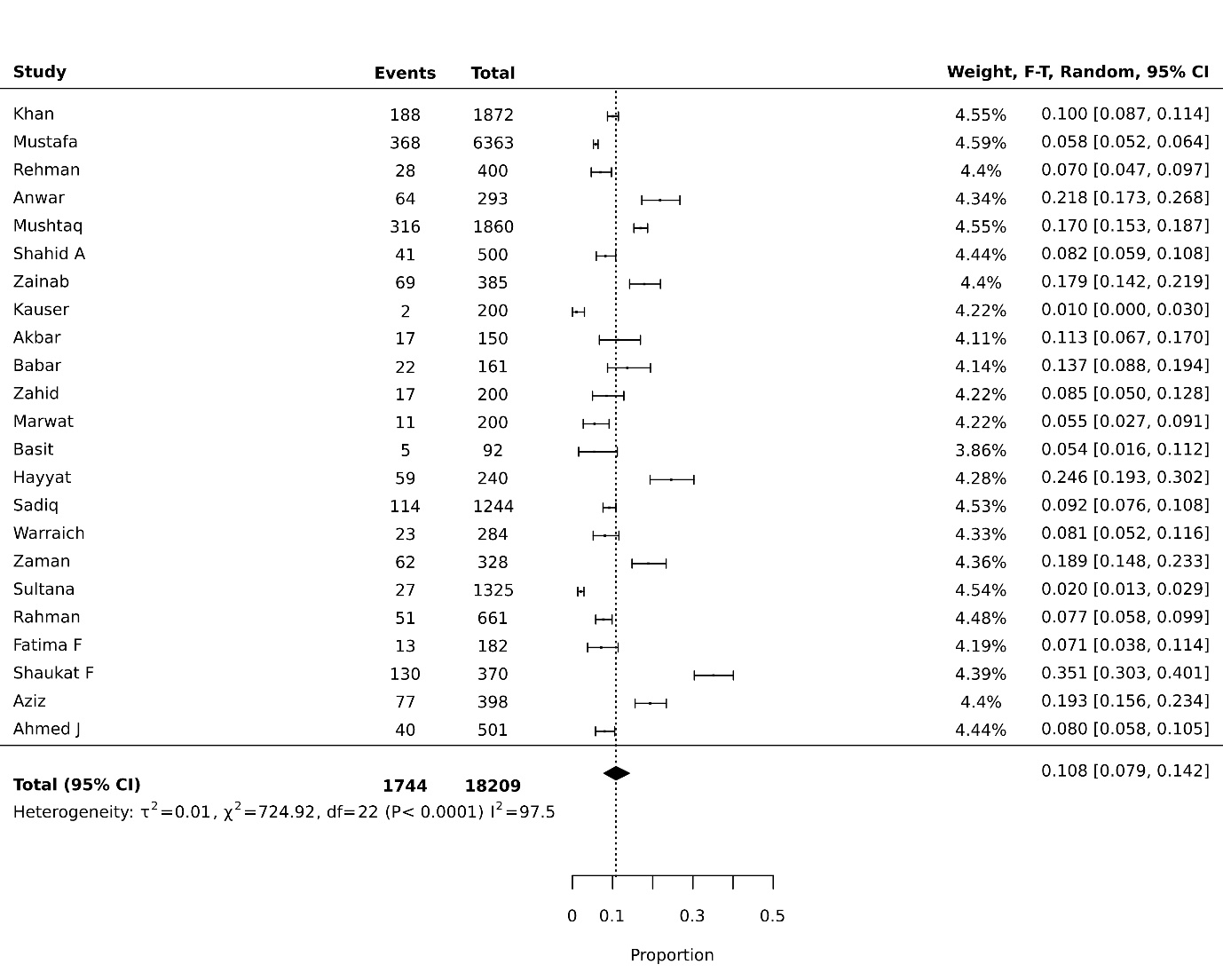


**Fig 1f: obese pooled prevalence in 0 to 19 years**


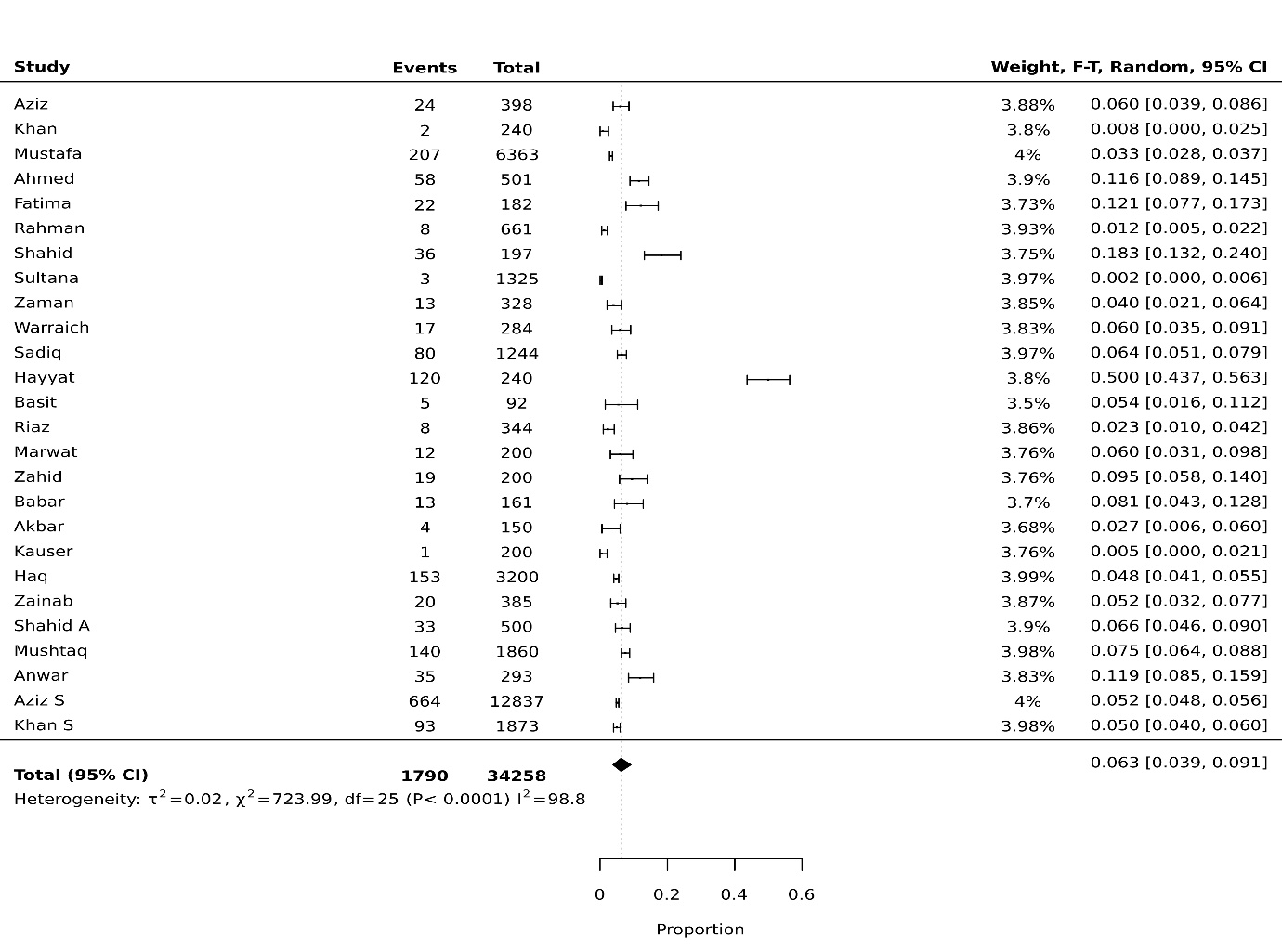


**Fig 2a: underweight pooled prevalence in 5 to 19 years**


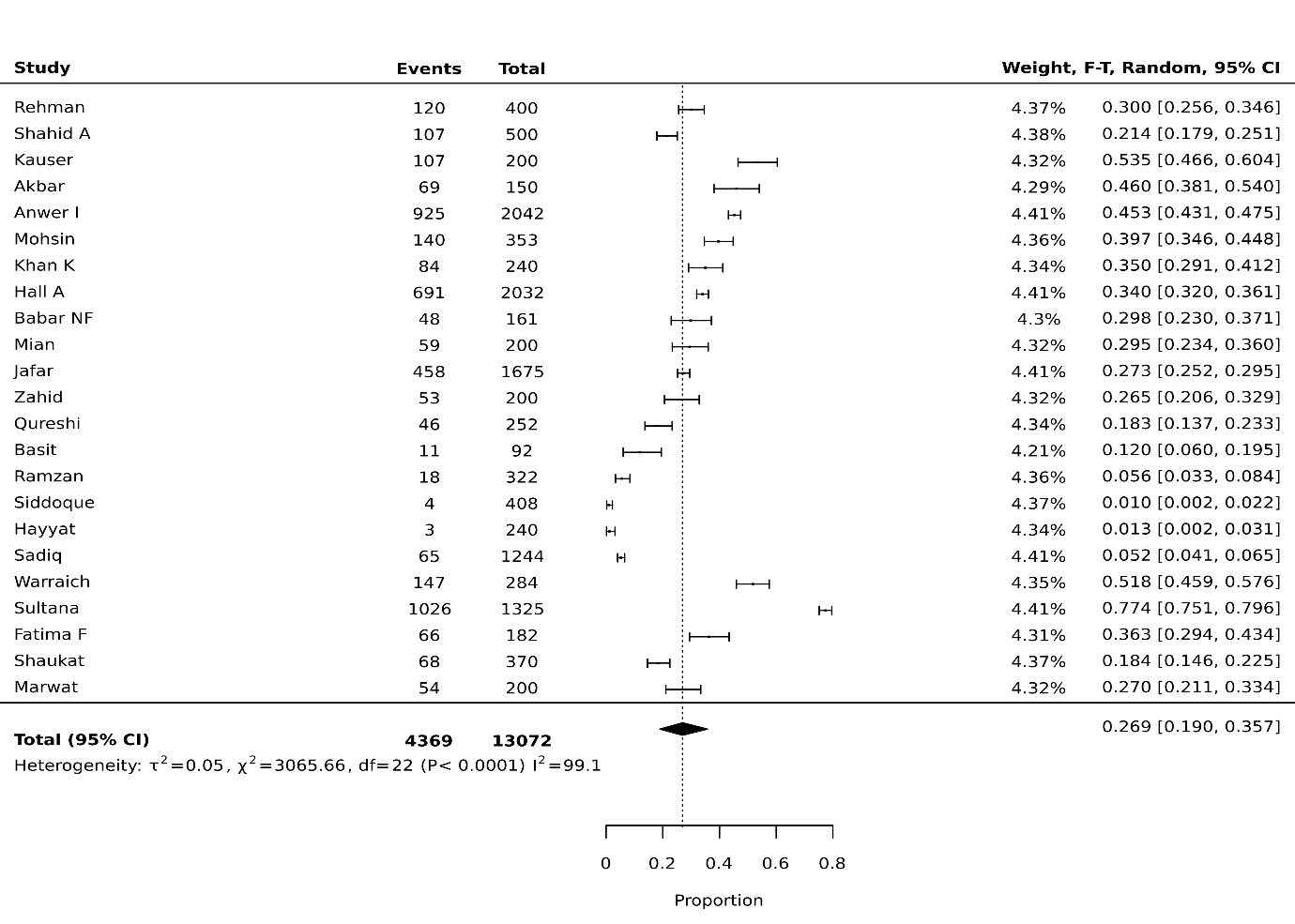


**Fig 2b: stunting pooled prevalence in 5 to 19 years**


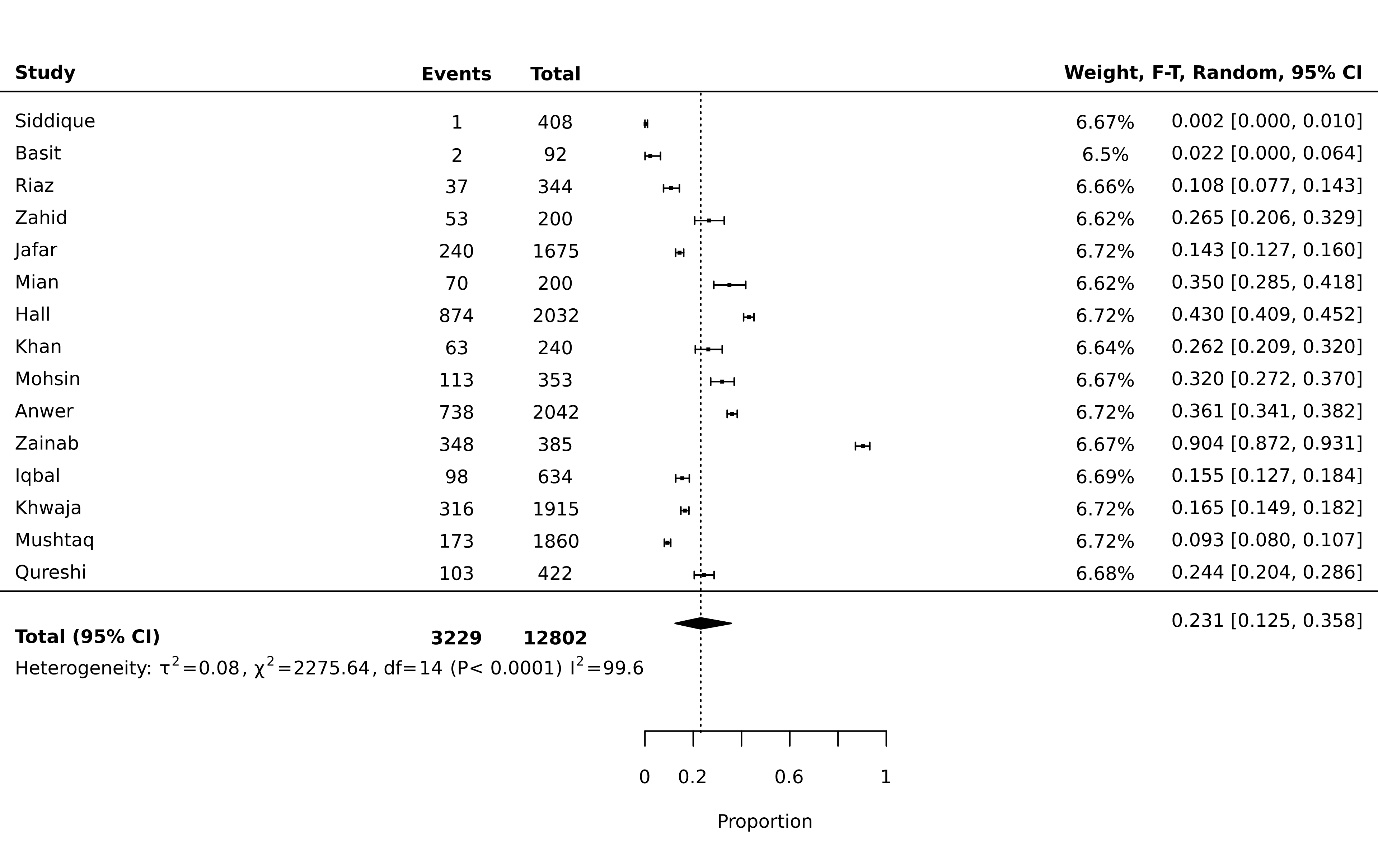


**Figure 2c: Wasting pooled prevalence in 5 to 19 years**


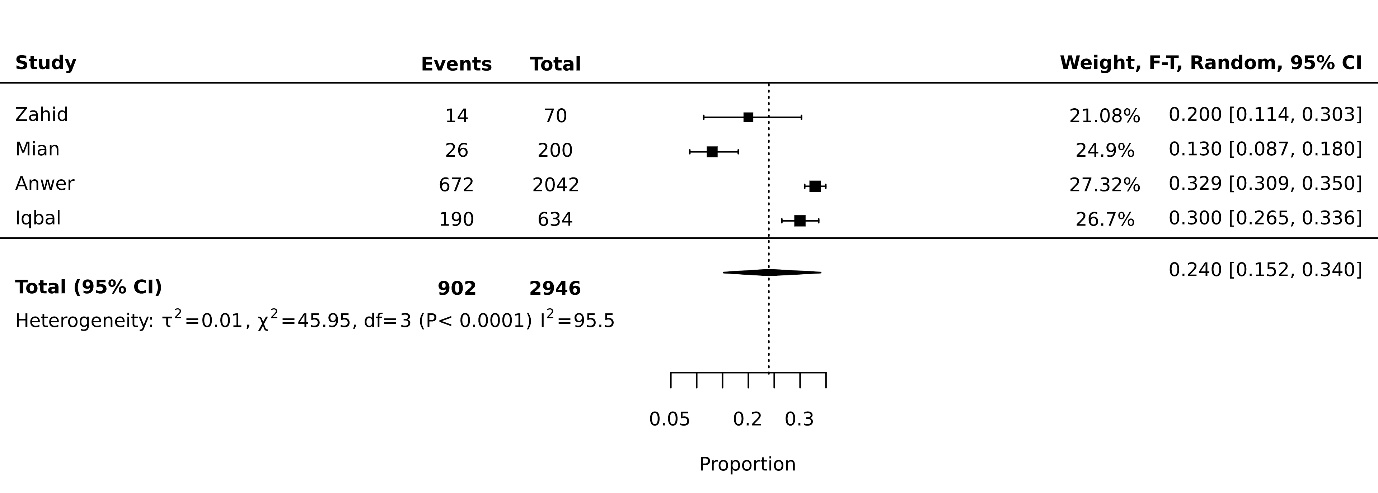


**Fig 2d: thinness pooled prevalence in 5 to 19 years**


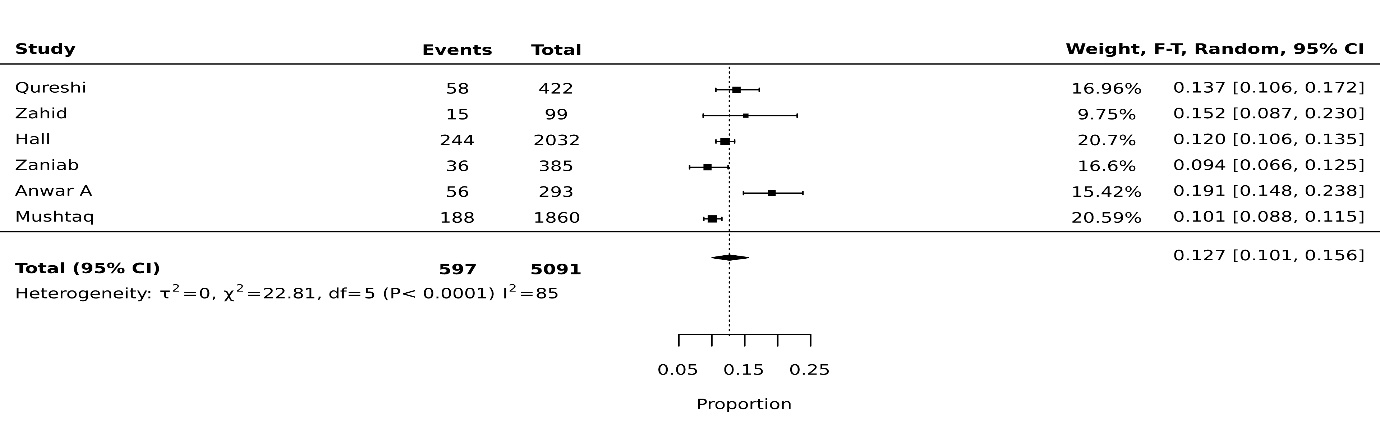


**Fig 2e: overweight pooled prevalence in 5 to 19 years**


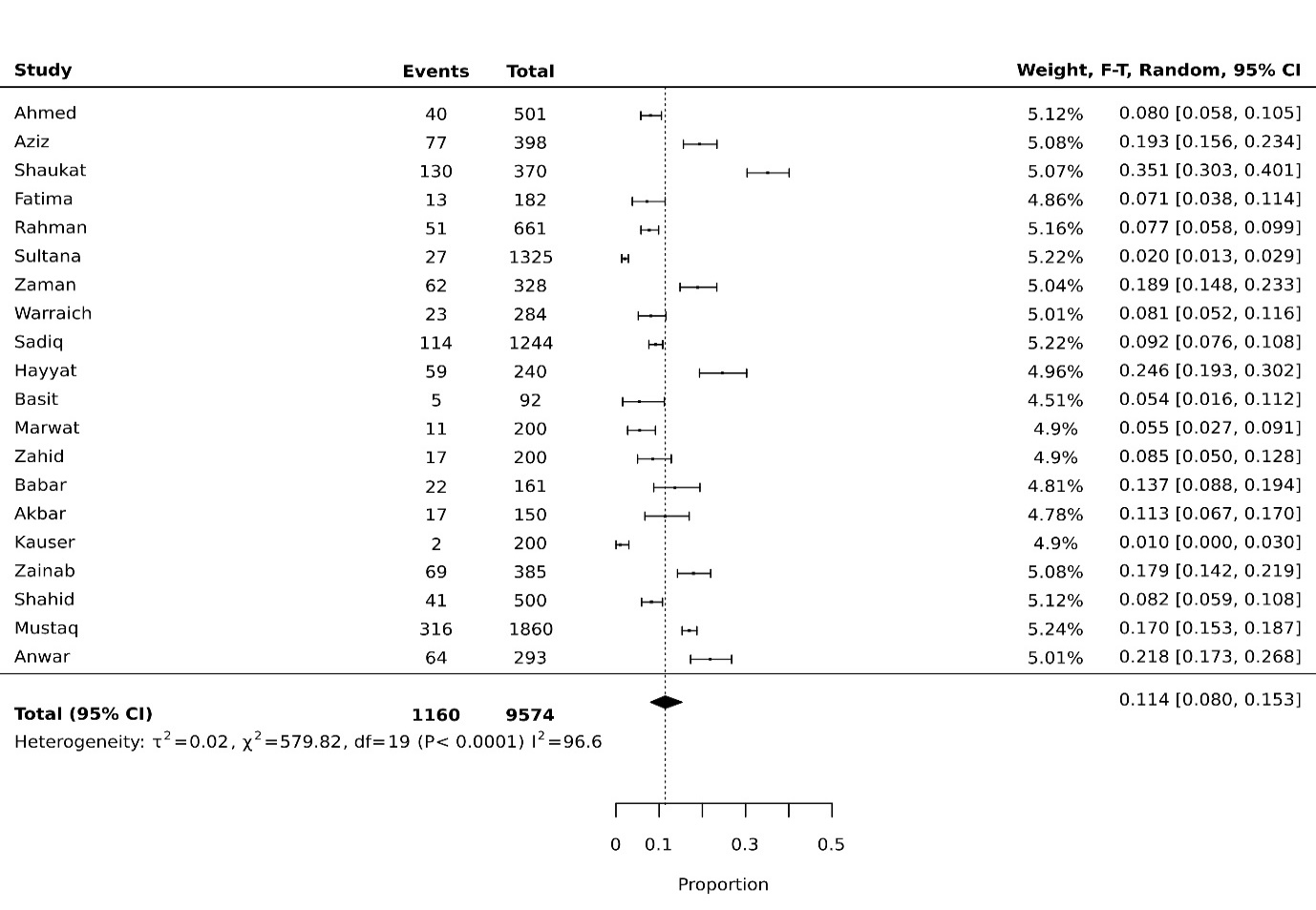


**Fig 2f: obese pooled prevalence in 5 to 19 years**


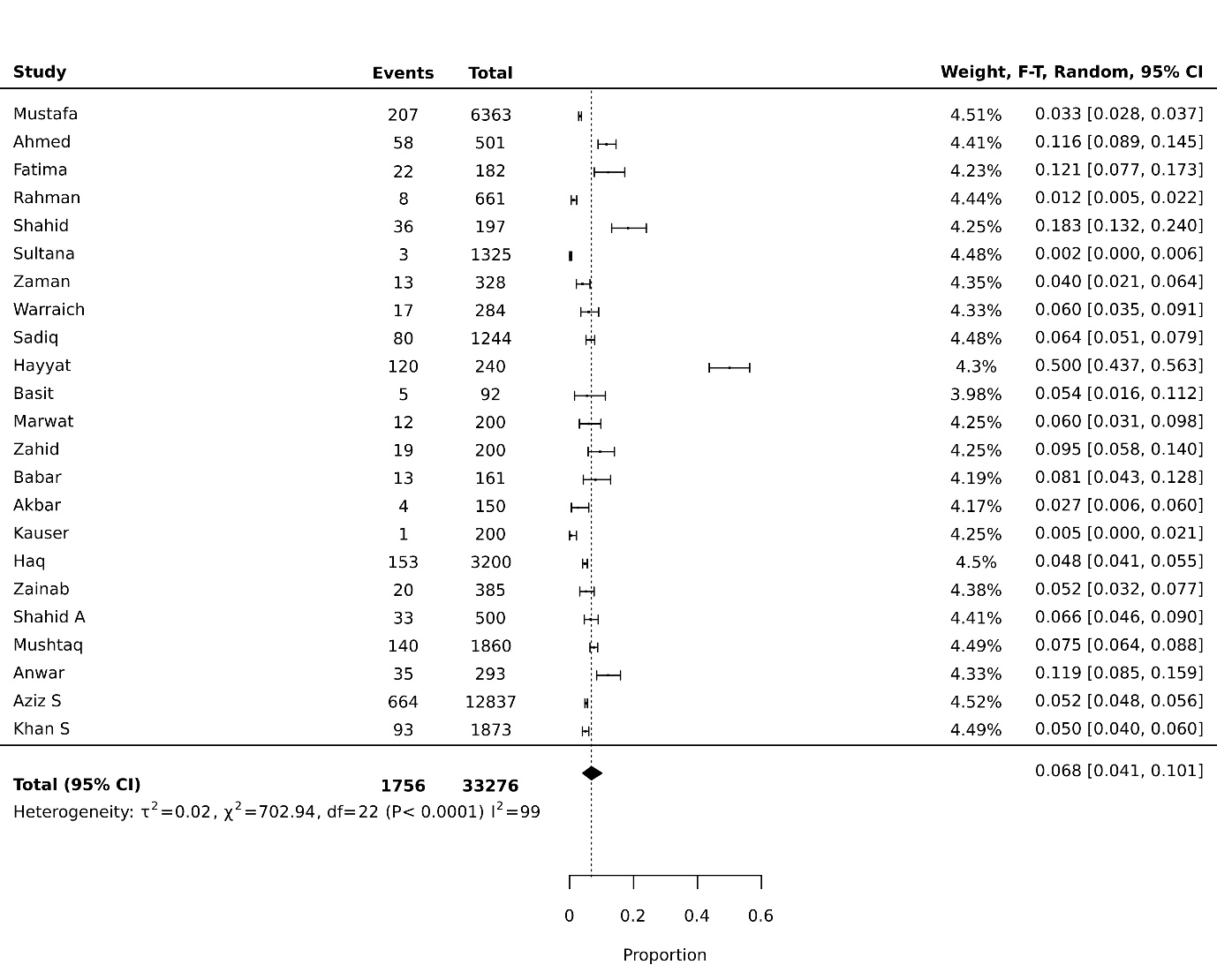


**Fig 3a: underweight pooled prevalence in 5 to 15 years**


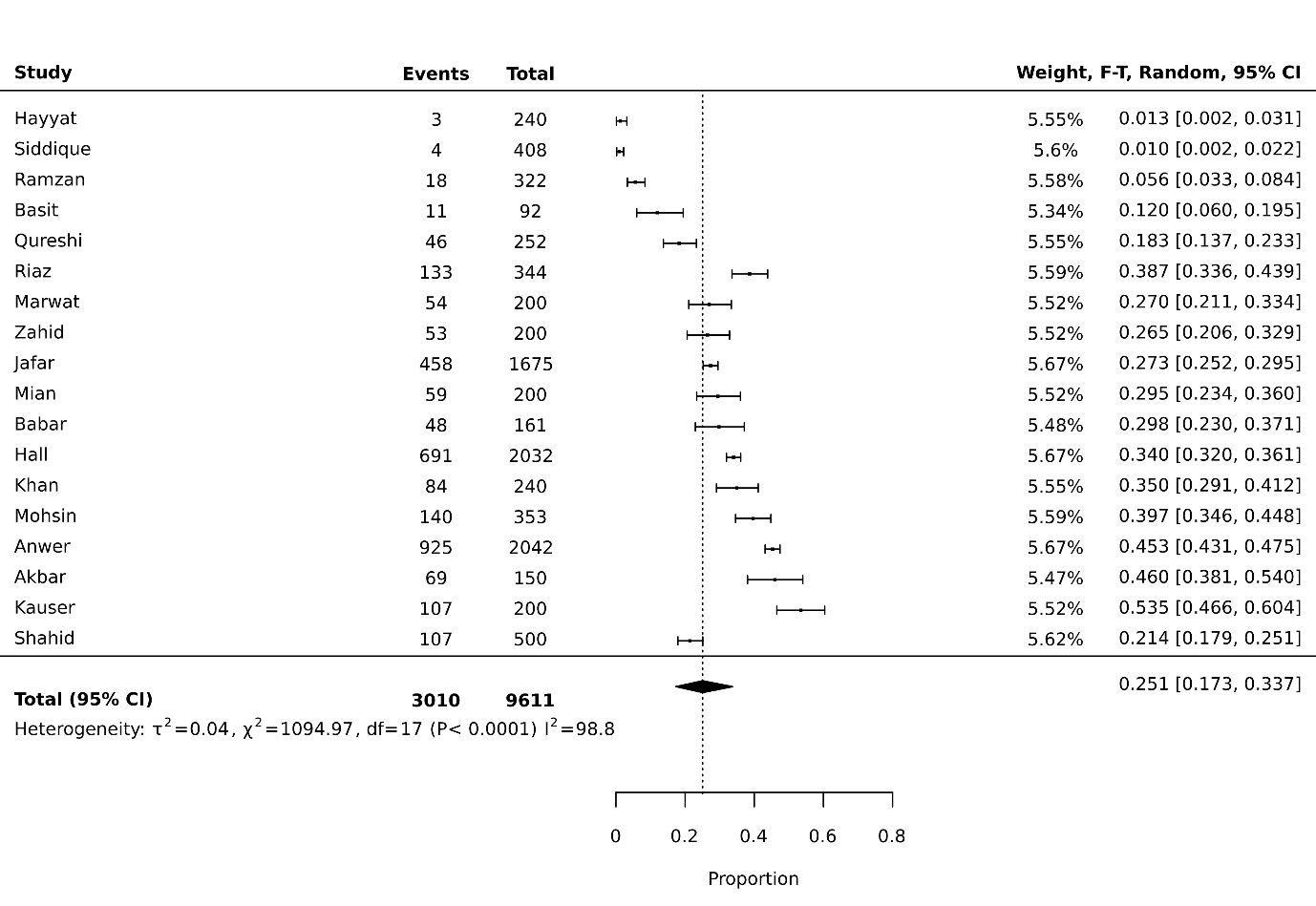
**Fig 3b: stunting pooled prevalence in 5 to 15 years**


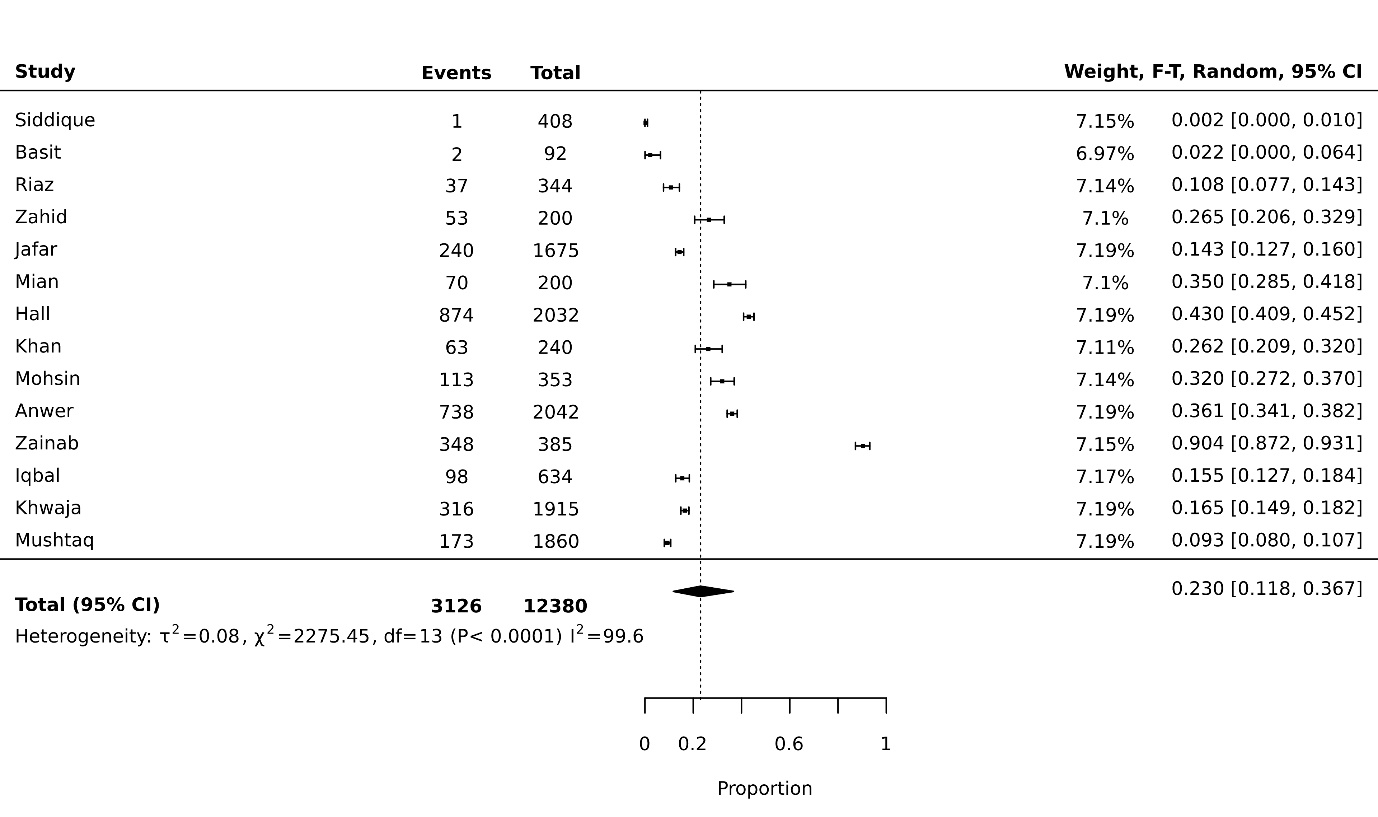


**Fig 3c: wasting pooled prevalence in 5 to 15 years**


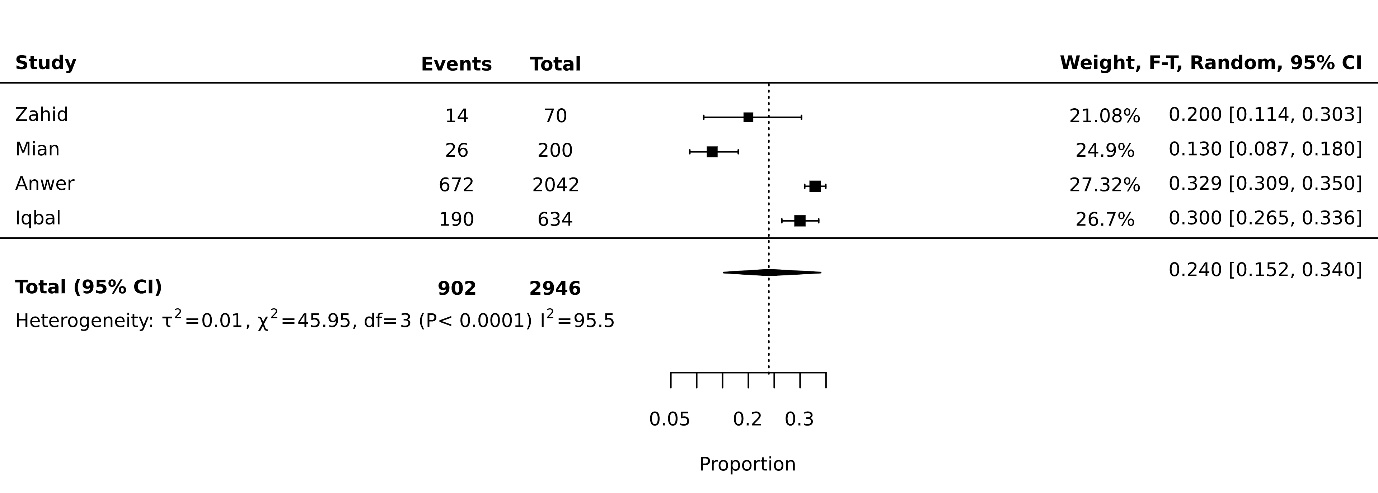


**Fig 3d: thinness pooled prevalence in 5 to 15 years**


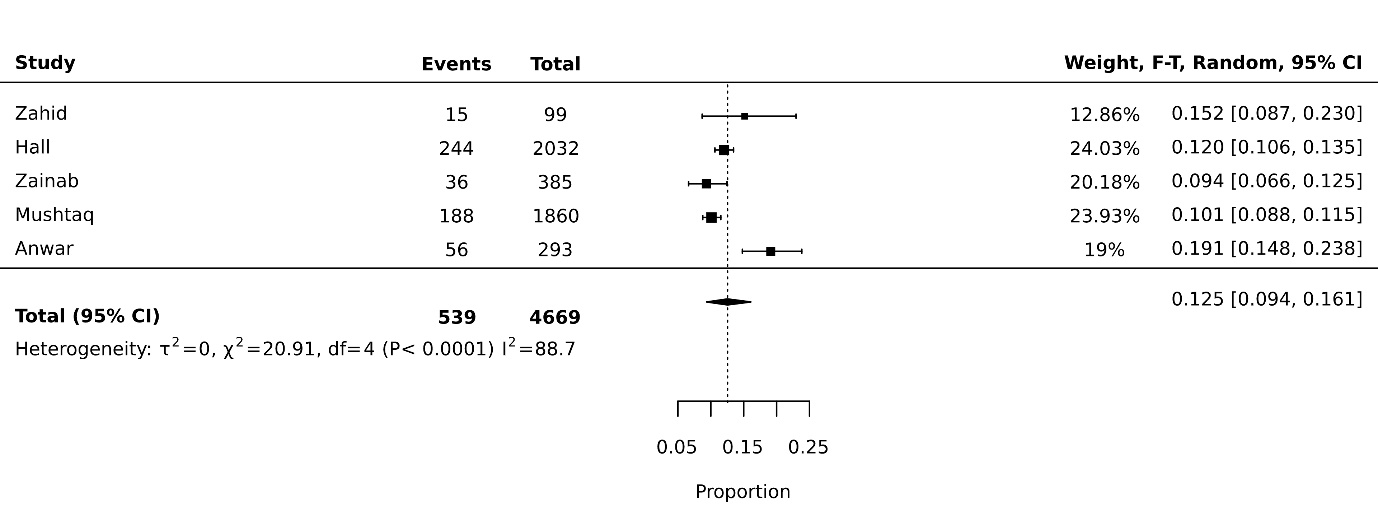


**Fig3e: overweight pooled prevalence in 5 to 15 years**


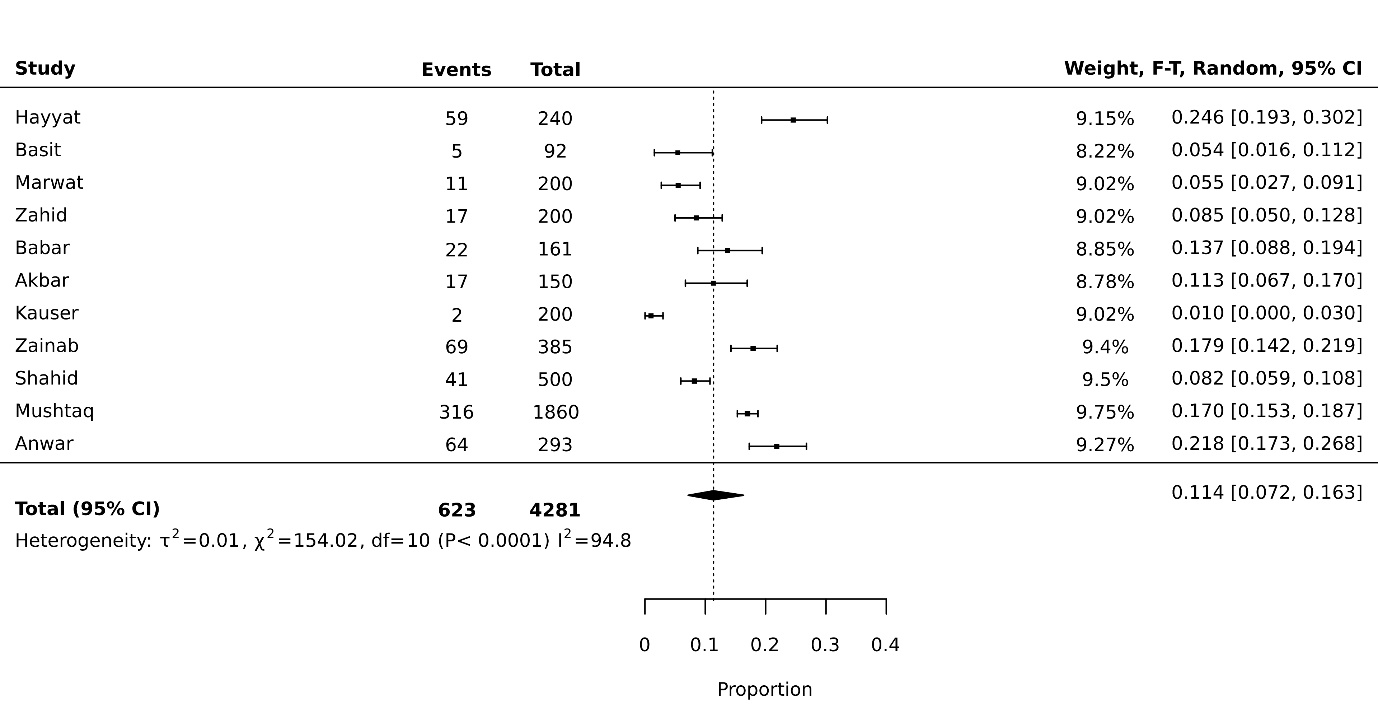


**Fig 3f: obese pooled prevalence in 5 to 15 years**


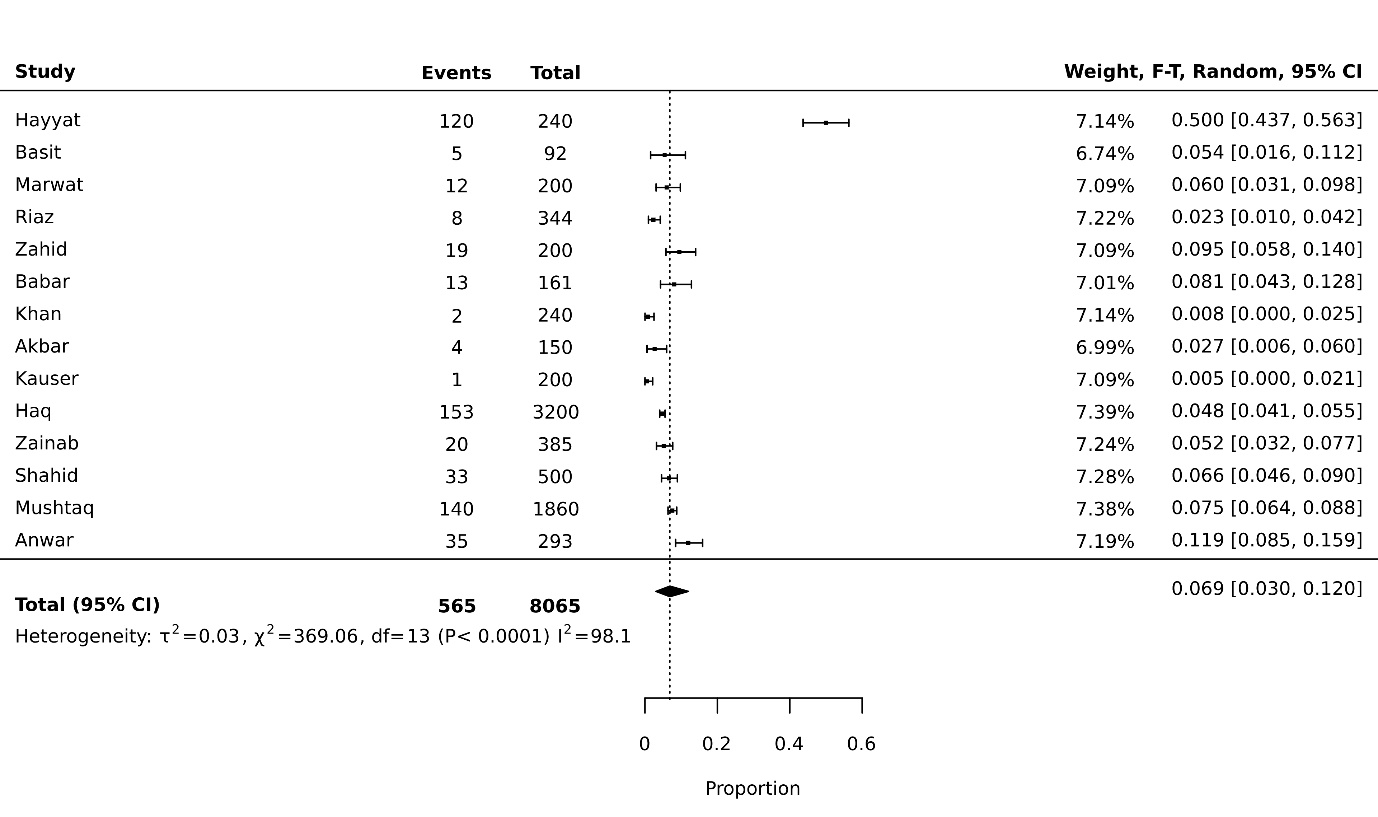


**Pooled prevalence analysis of anthropometric indices in children age 5 to 15 years with subcategories:**

**Fig 4: Underweight**

**Fig 4a: Underweight pooled prevalence in female gender**


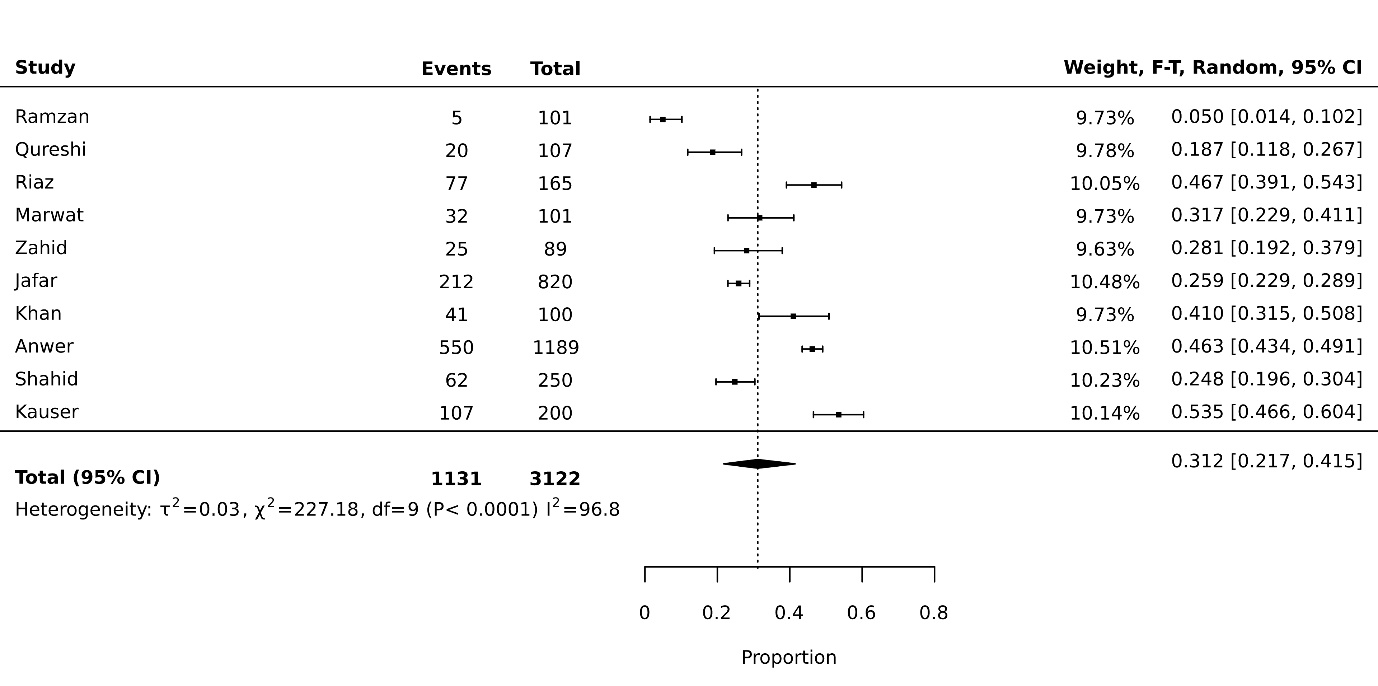


**Fig 4b: Underweight pooled prevalence in male gender**


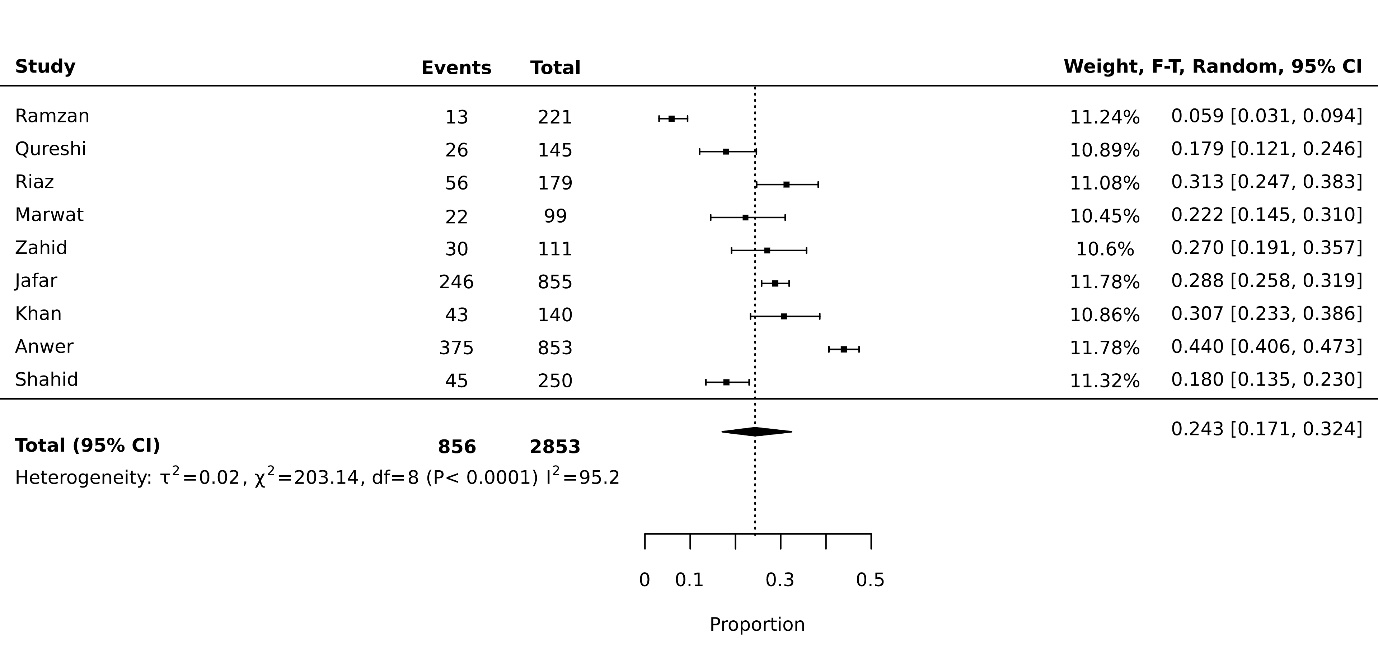


**Fig 4c: Underweight pooled prevalence in children going to private schools**


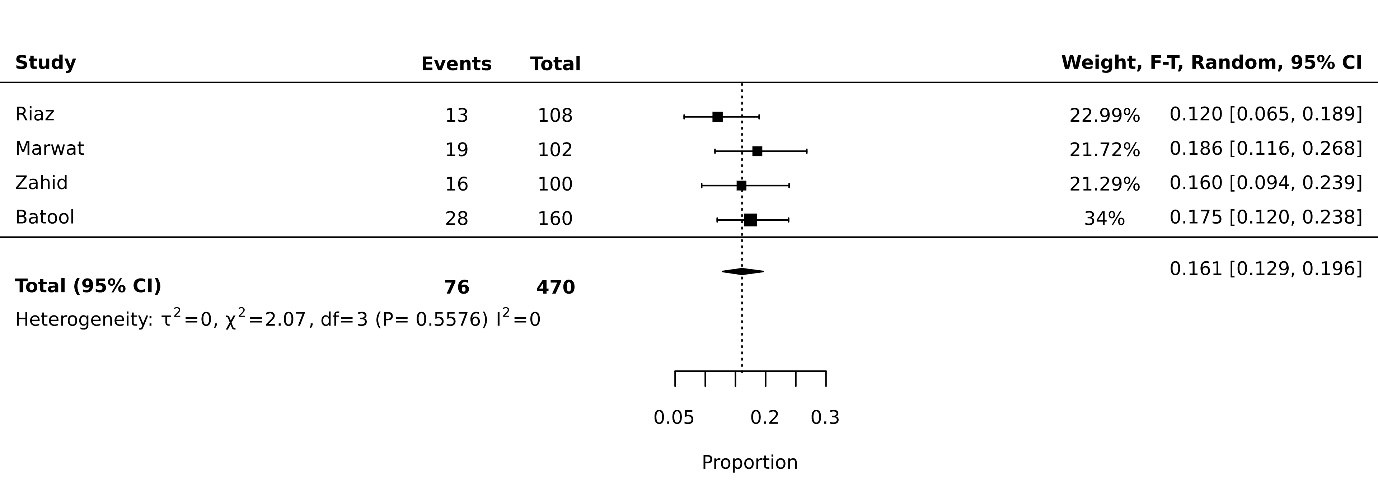


**Fig 4d: Underweight pooled prevalence in children going to government schools**


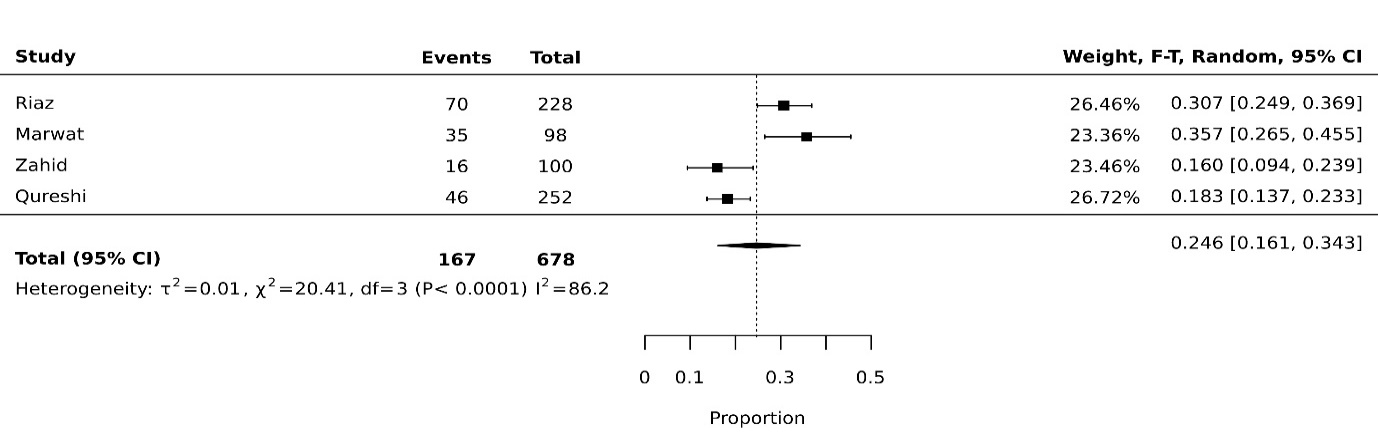


**Fig 4e: Underweight pooled prevalence in children from rural setting**


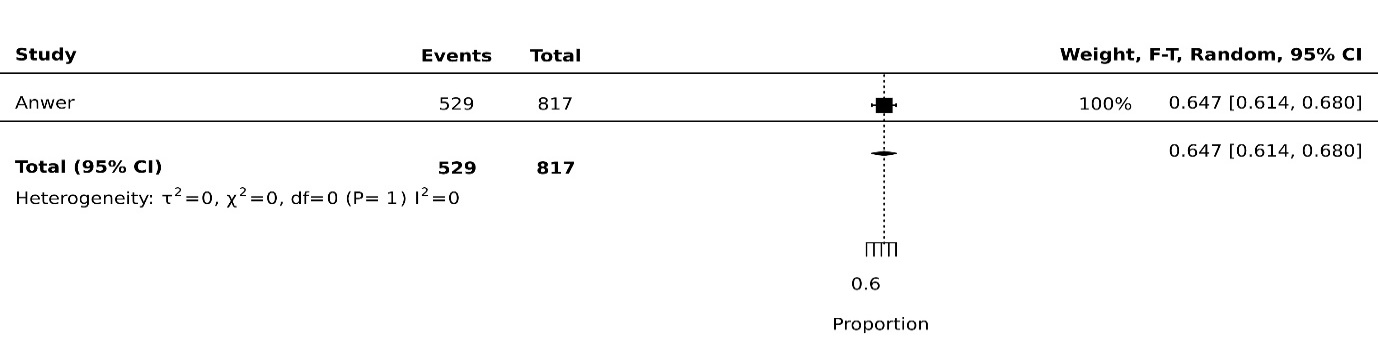


**Fig 4f: Underweight pooled prevalence in children from urban setting**


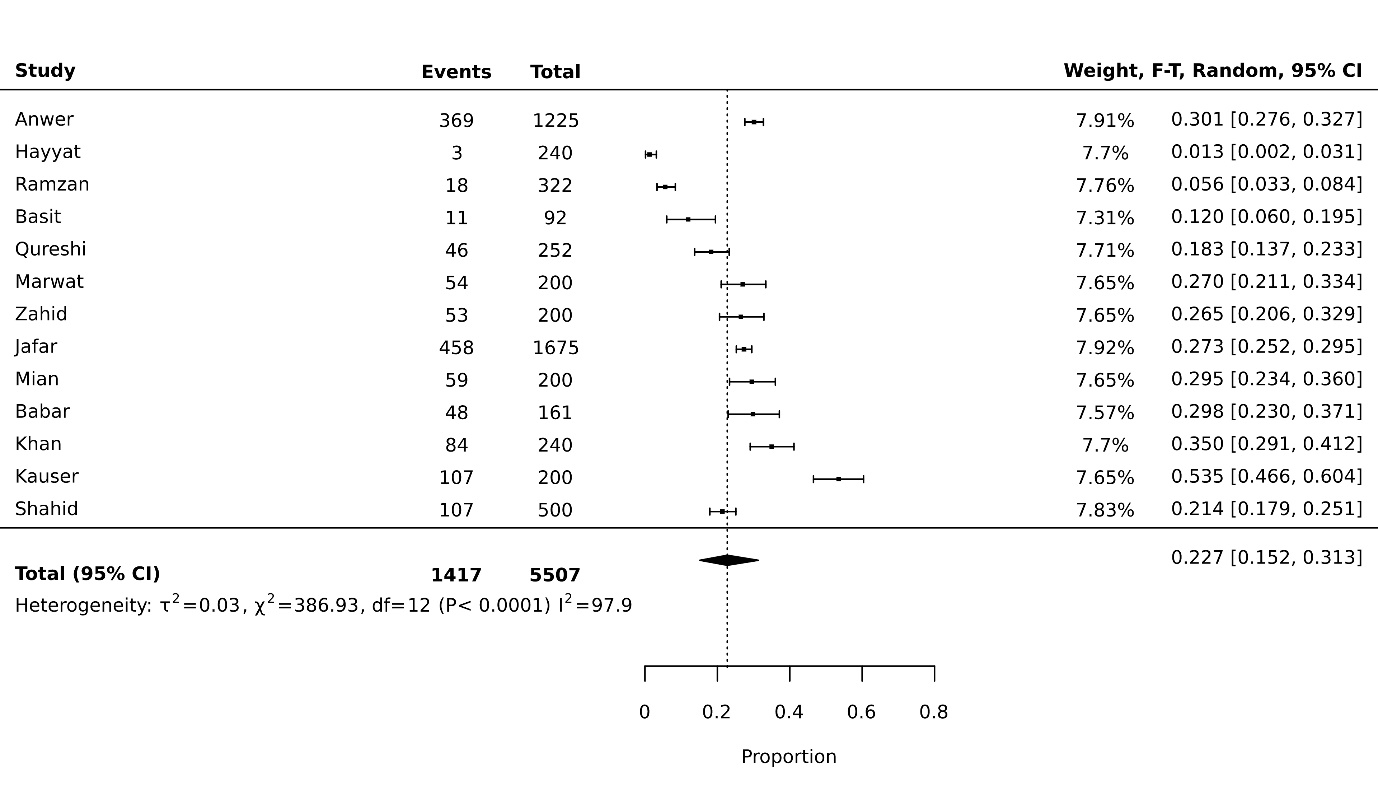


**Fig 4g: Underweight pooled prevalence in children from low socioeconomic status**


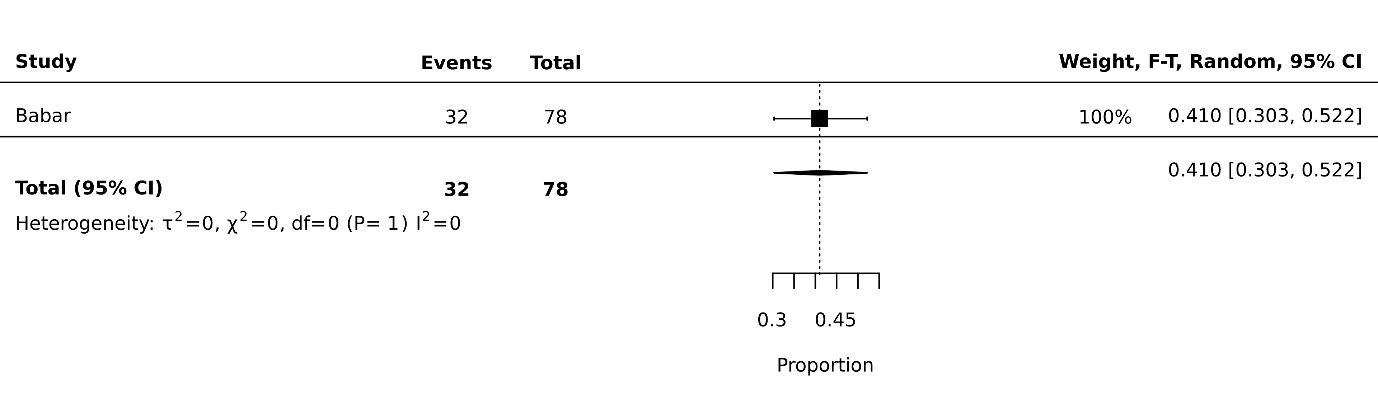


**Fig 4h: Underweight pooled prevalence in children from high socioeconomic status**


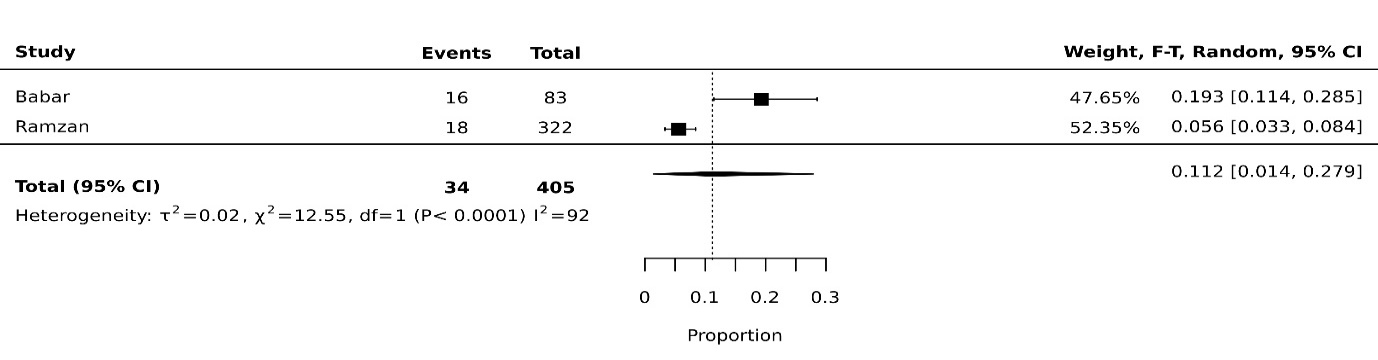


**Fig 4i: Underweight pooled prevalence in children from disaster affected regions**


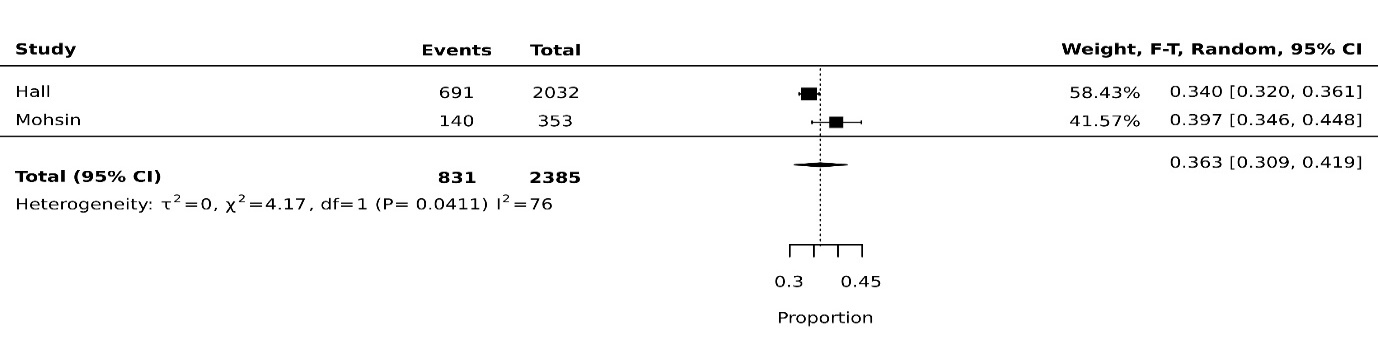


**Fig 4j: Underweight pooled prevalence in children from Sindh**
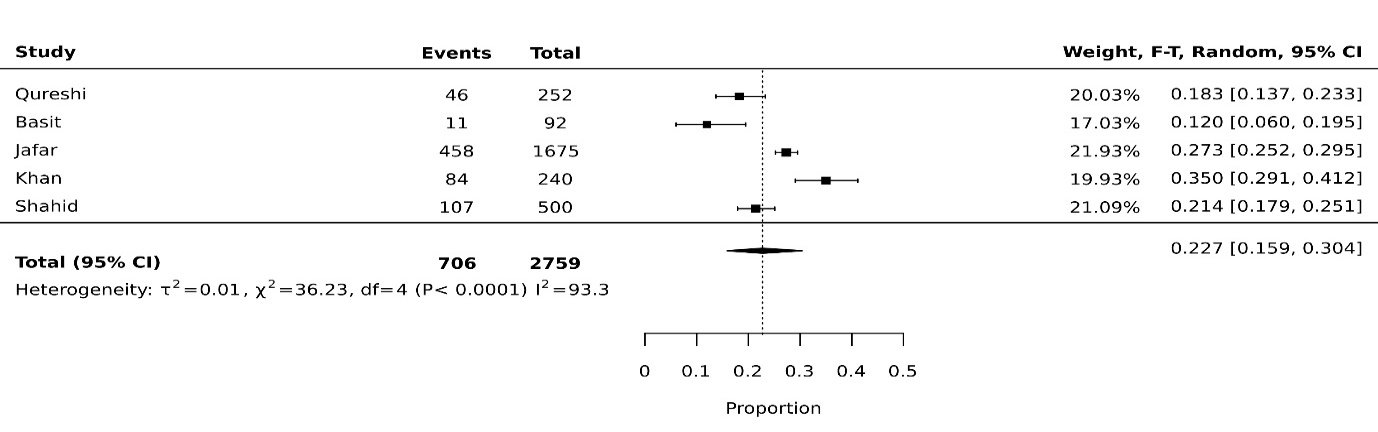


**Fig 4k: Underweight pooled prevalence in children from Punjab**
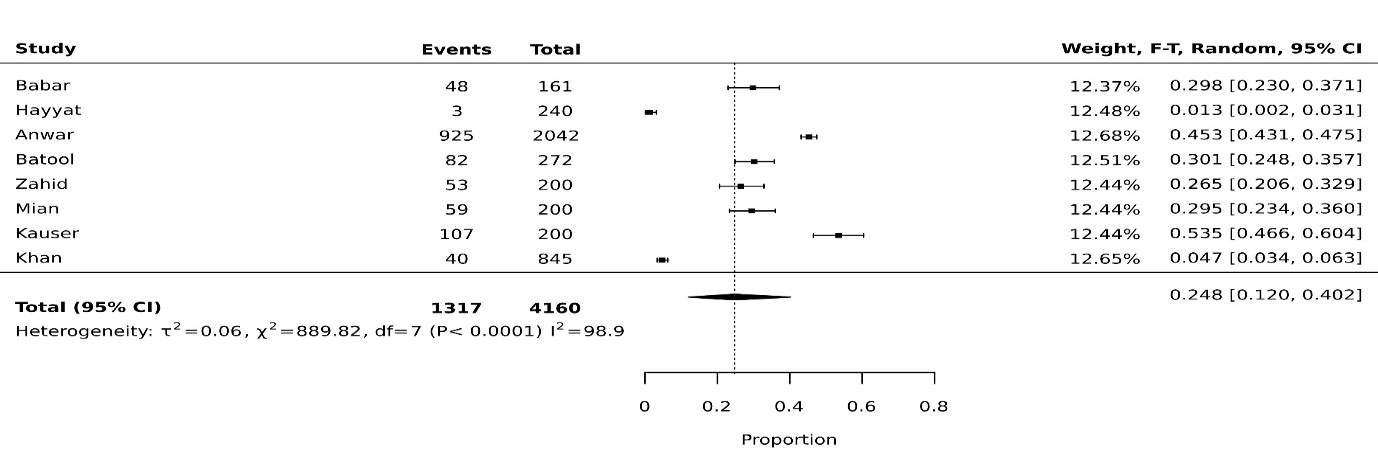


**Fig 4l: Underweight pooled prevalence in children from KP**


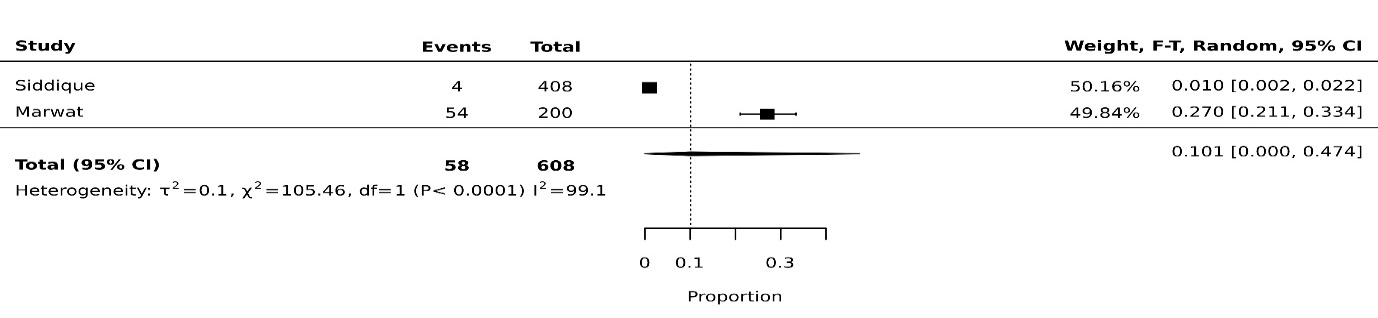


**Figure 5: Stunting**

**Fig 5a: Stunting pooled prevalence in female gender**
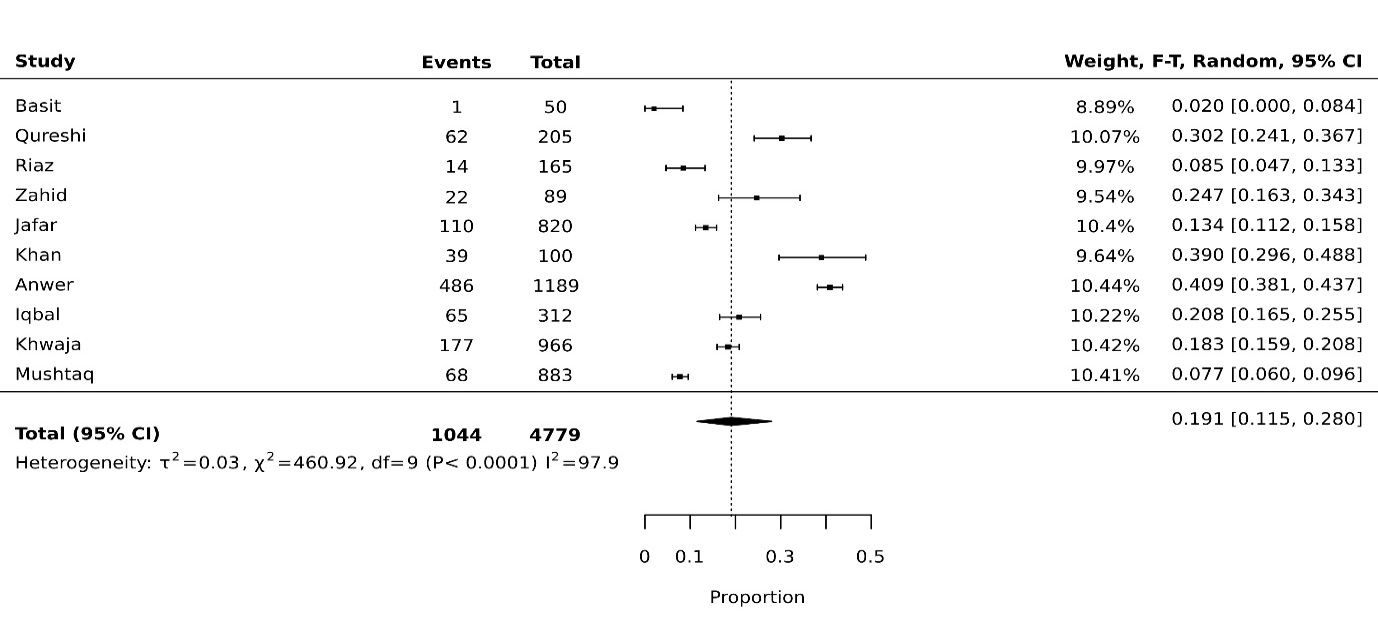
 **Fig 5b: Stunting pooled prevalence in male gender**


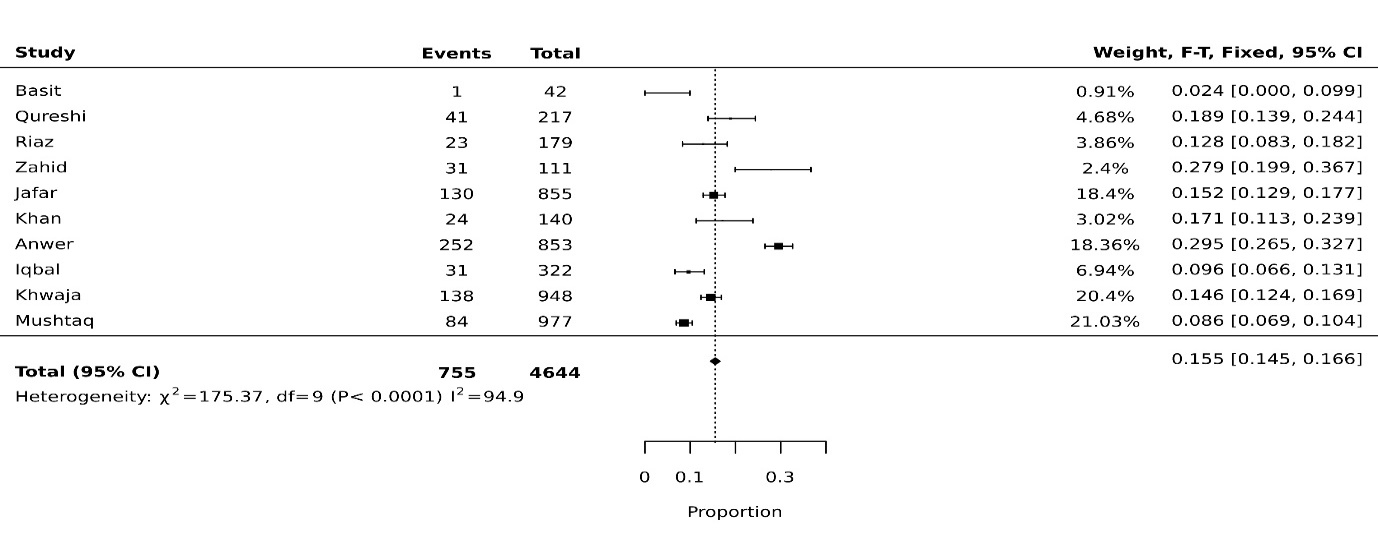


**Fig 5c: Stunting pooled prevalence in children going to private schools**


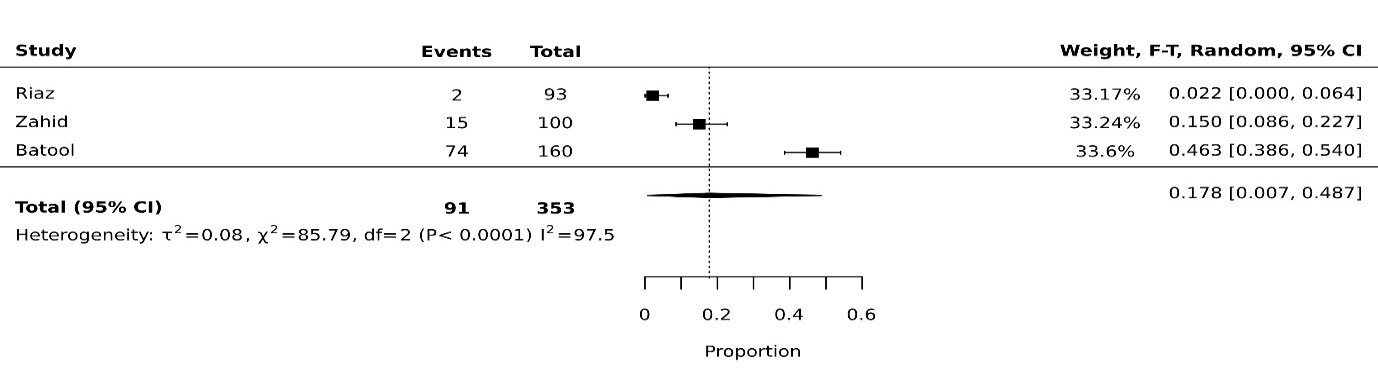


**Fig 5d: Stunting pooled prevalence in children going to government schools**


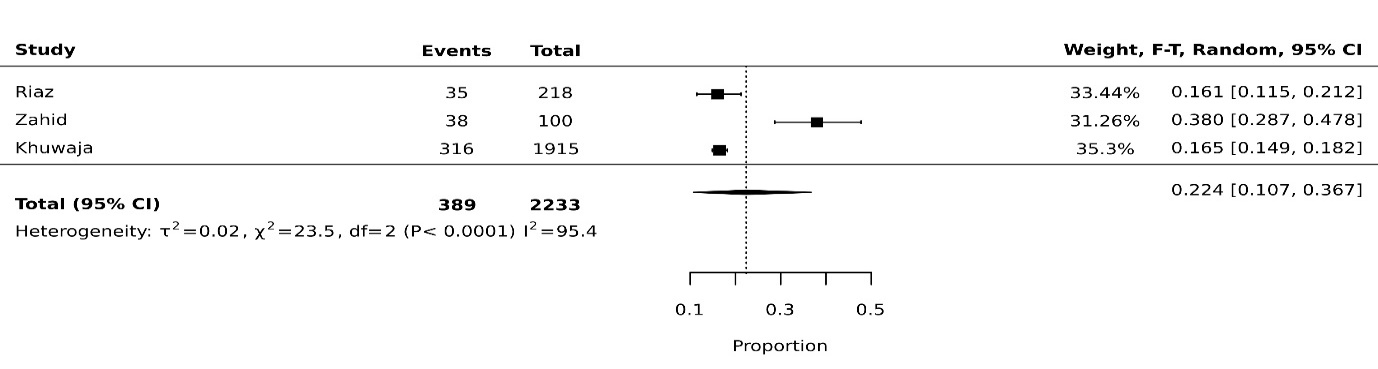


**Fig 5e: Stunting pooled prevalence in children from rural setting**


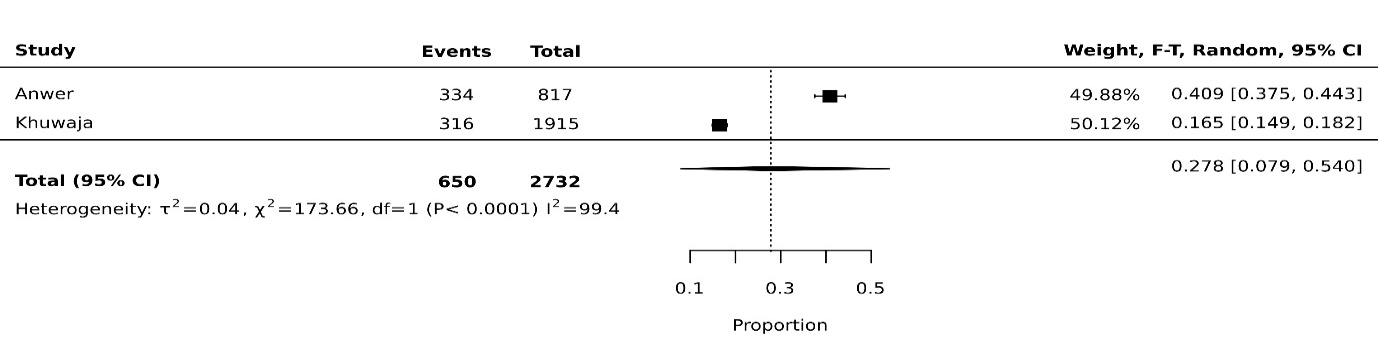


**Fig 5f: Stunting pooled prevalence in children from urban setting**


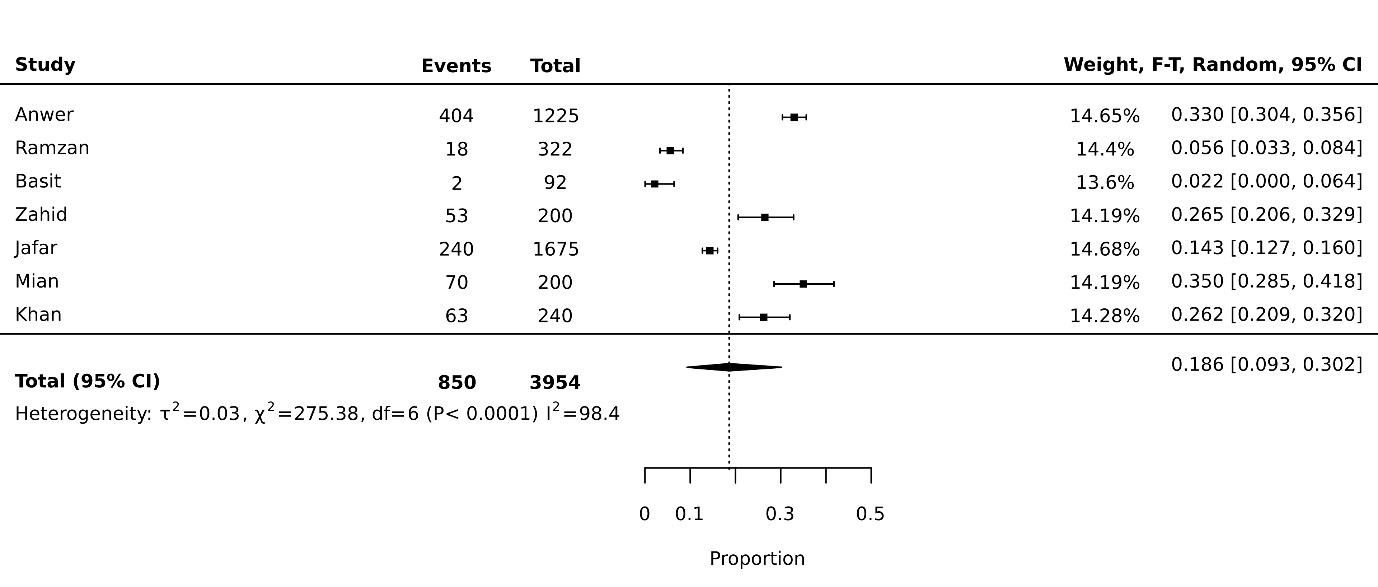


**Fig 5g: Stunting pooled prevalence in children from low socioeconomic setting**


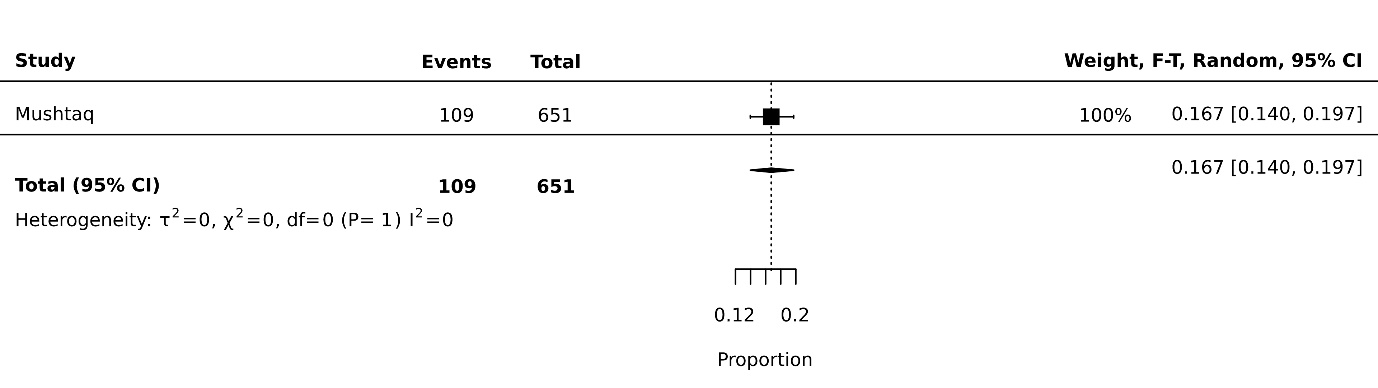


**Fig 5h: Stunting pooled prevalence in children from high socioeconomic setting**


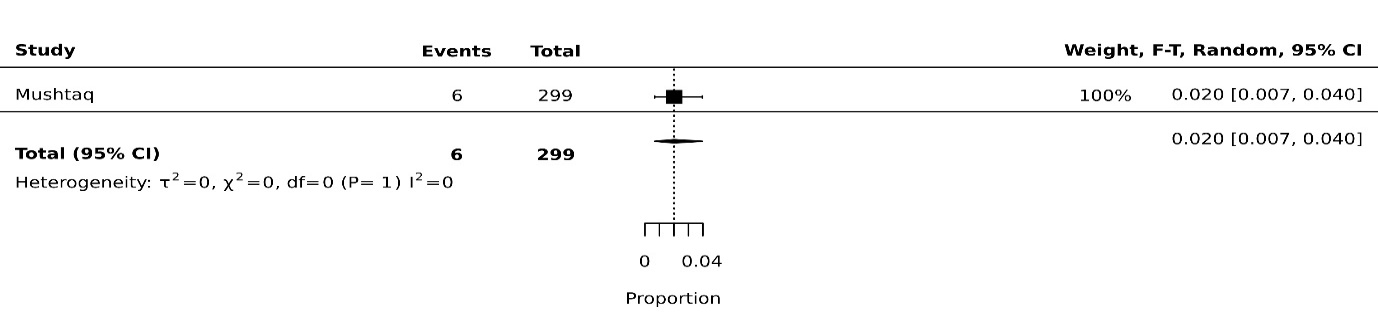


**Fig 5i: Stunting pooled prevalence in children from disaster affected regions**


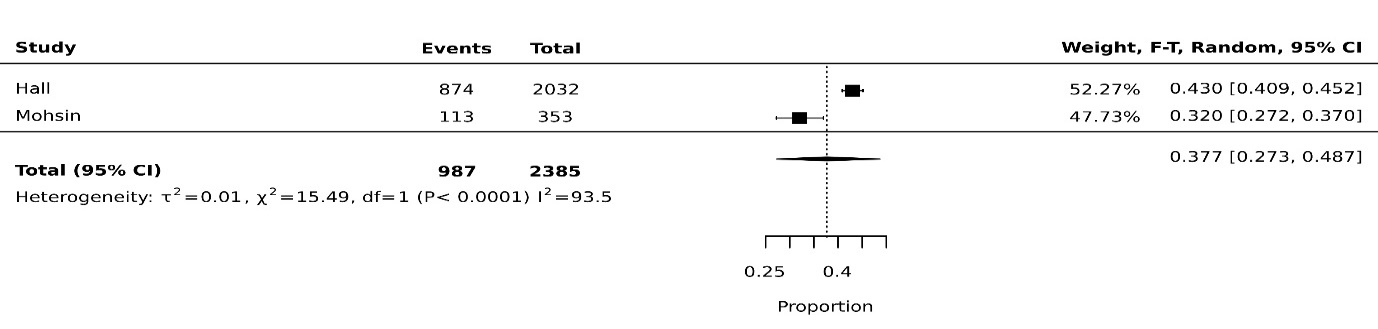


**Fig 5j: Stunting pooled prevalence in children employed as laborers**


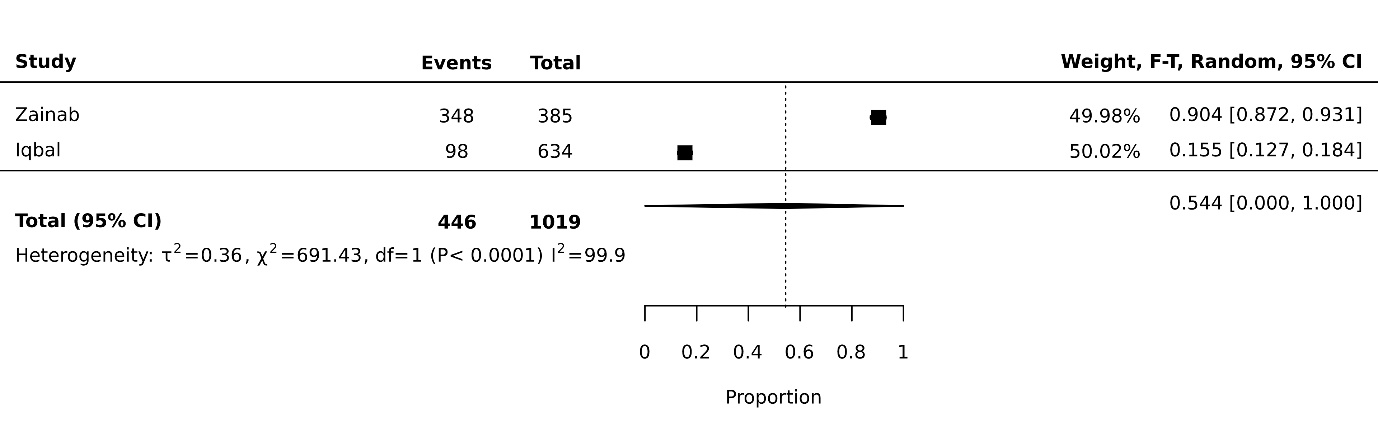


**Fig 5k: Stunting pooled prevalence in children from Sindh**


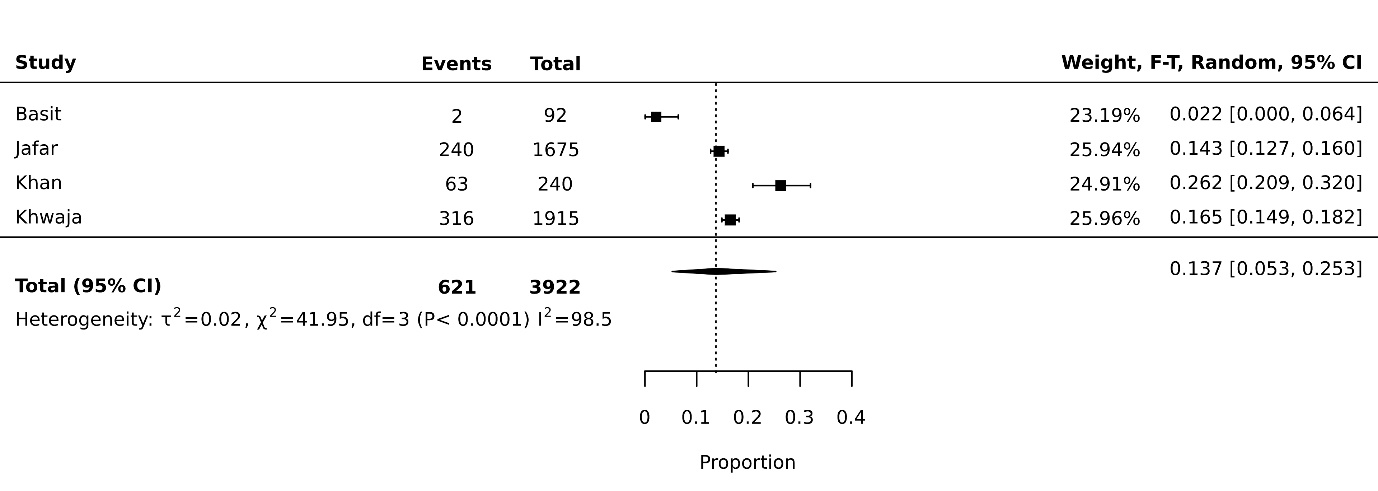


**Fig 5l: Stunting pooled prevalence in children from Punjab**


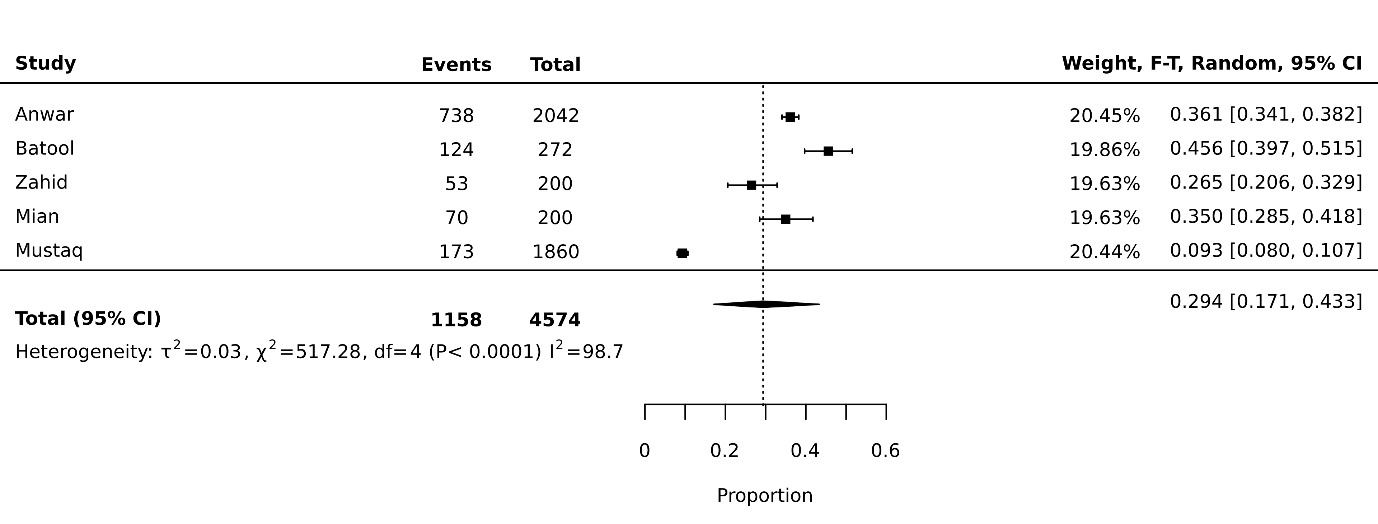


**Fig 5m: Stunting pooled prevalence in children from KP**


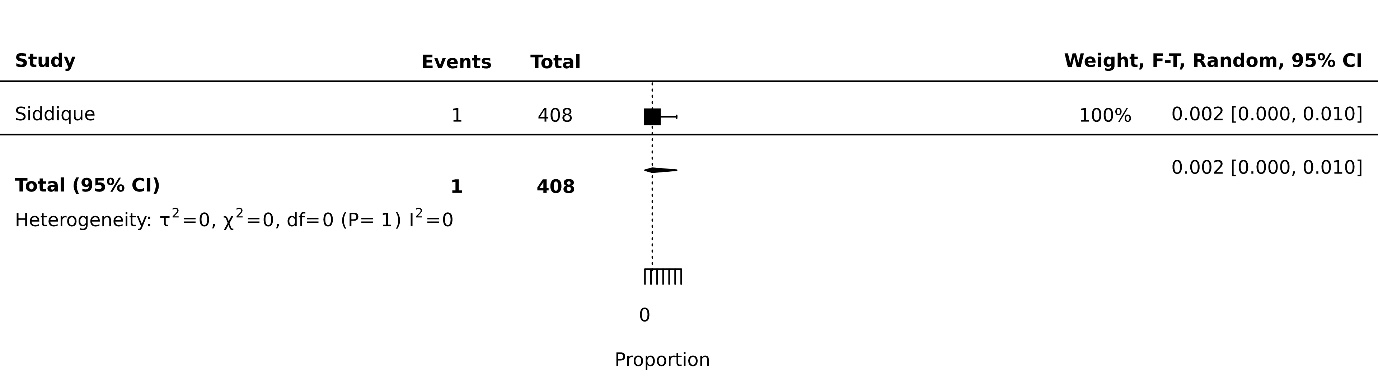


**Fig 6: Wasting**

**Fig 6a: Wasting pooled prevalence in female gender**


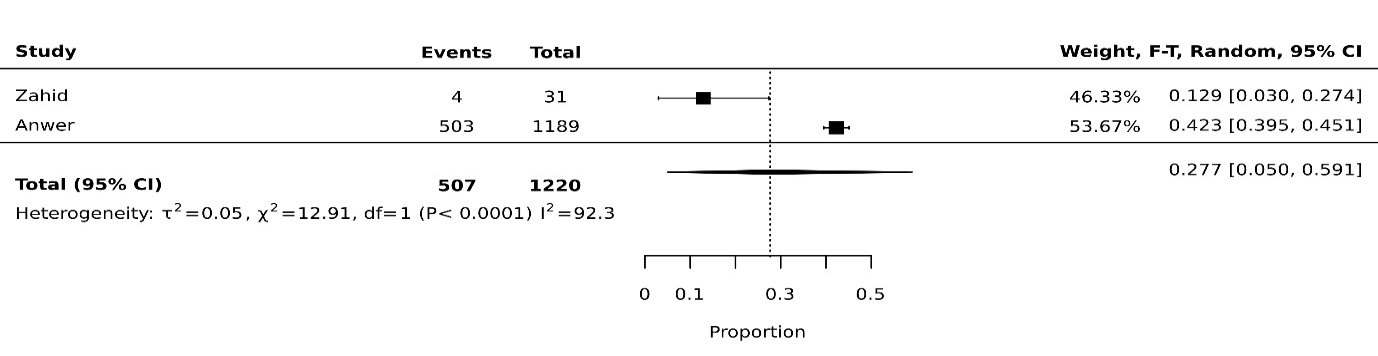


**Fig 6b: Wasting pooled prevalence in male gender**


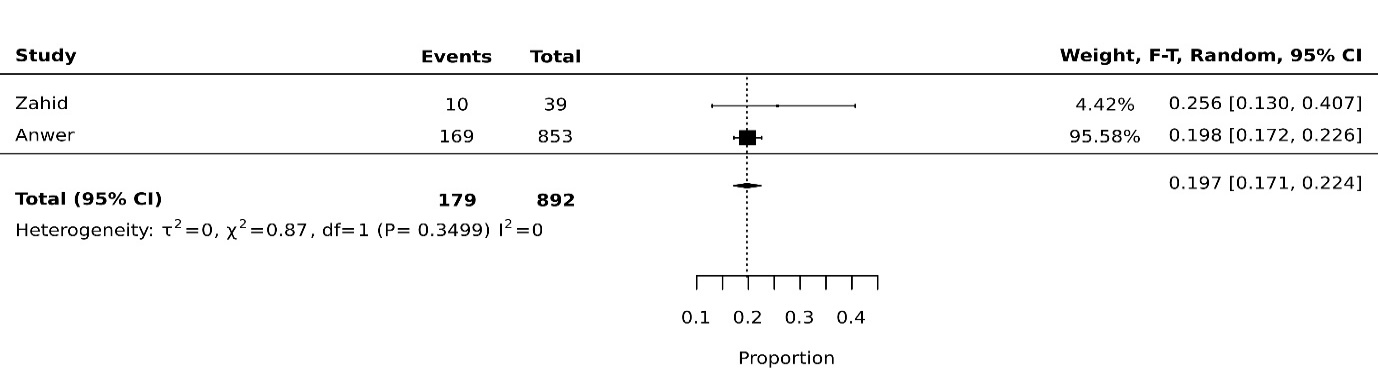
 **Fig 6c: Wasting pooled prevalence in children going to private schools**


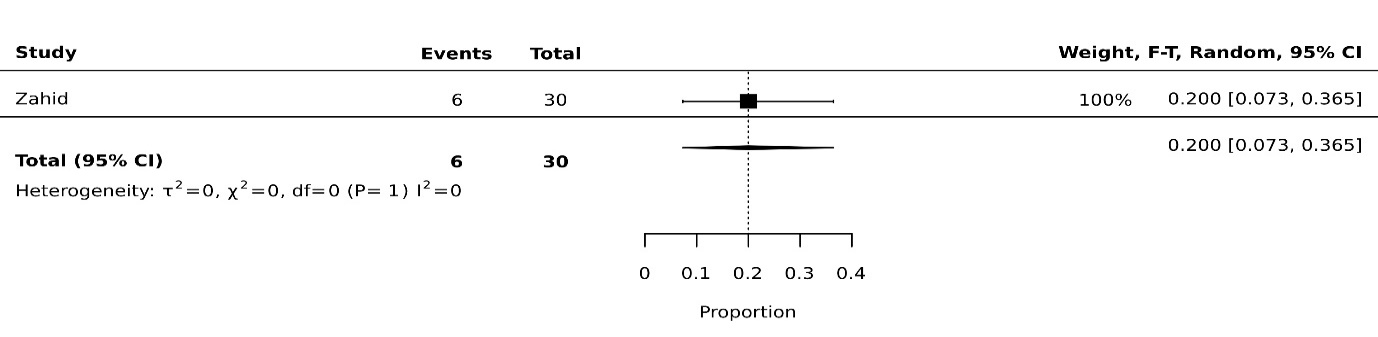
 **Fig 6d: Wasting pooled prevalence in children going to government schools**


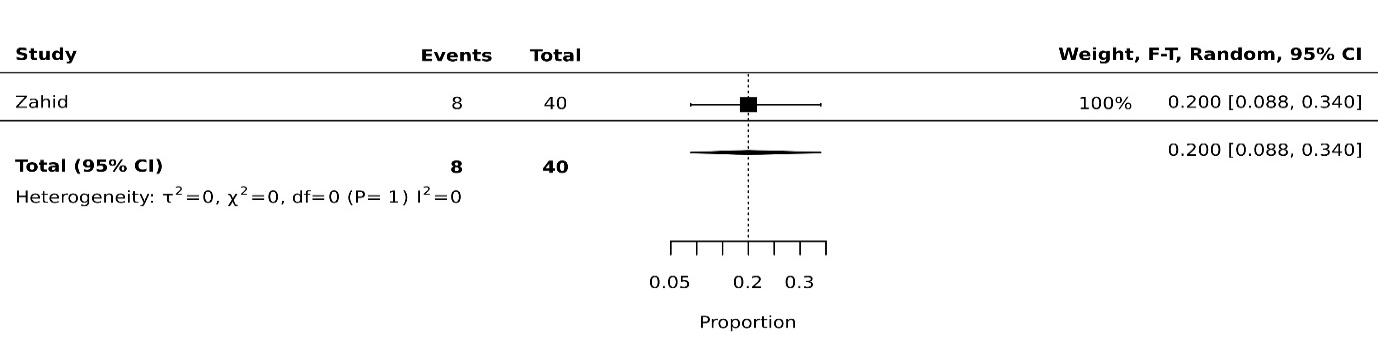


**Fig 6e: Wasting pooled prevalence in children from rural setting**


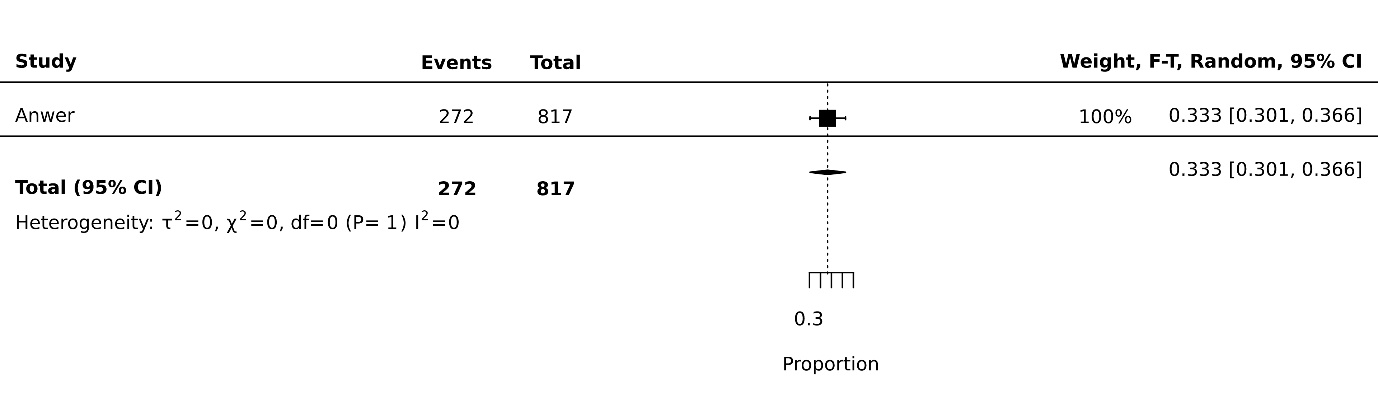


**Fig 6f: Wasting pooled prevalence in children from urban setting**


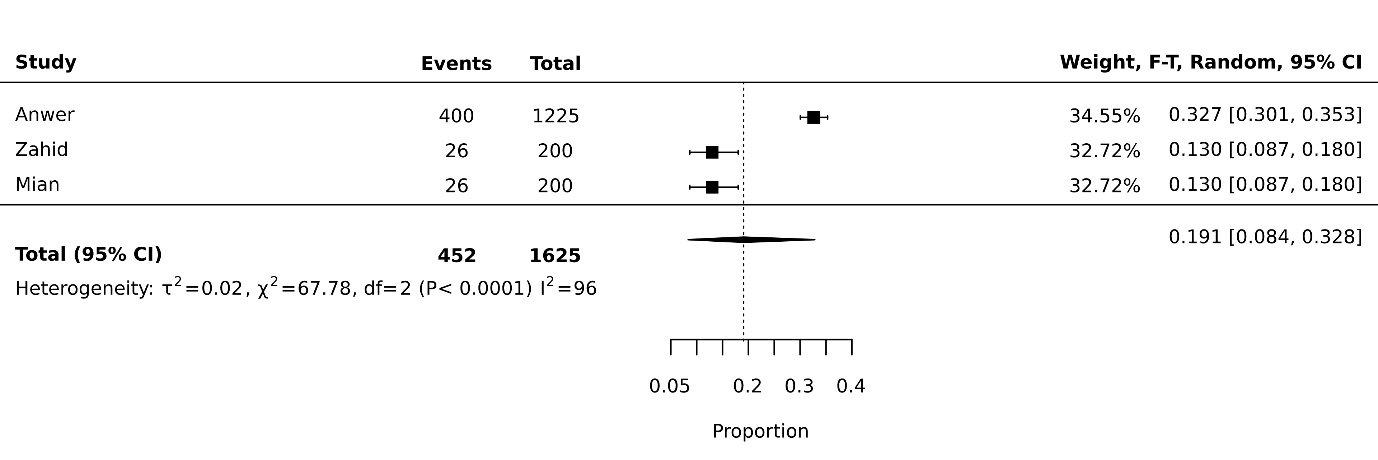


**Fig 6g: Wasting pooled prevalence in children employed as laborers**


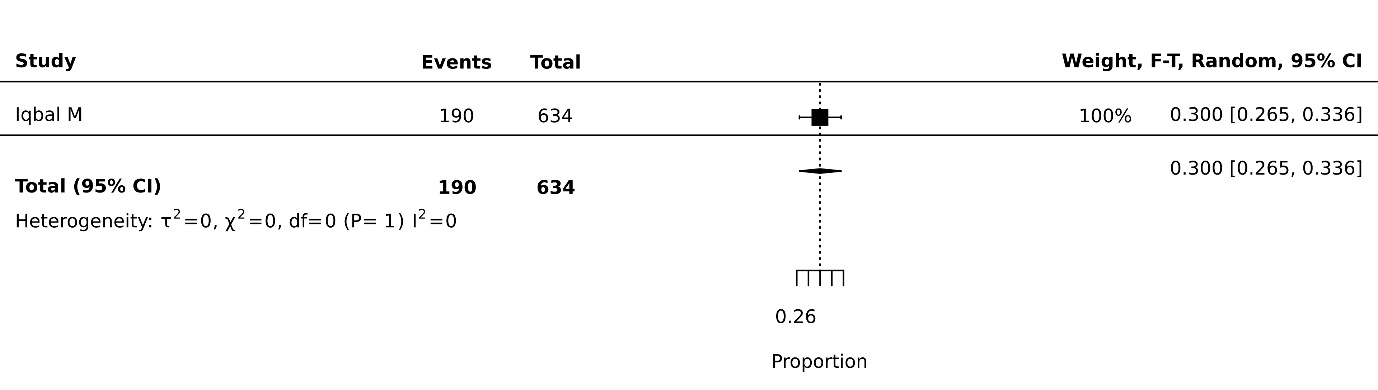


**Fig 6h: Wasting pooled prevalence in children from Punjab**


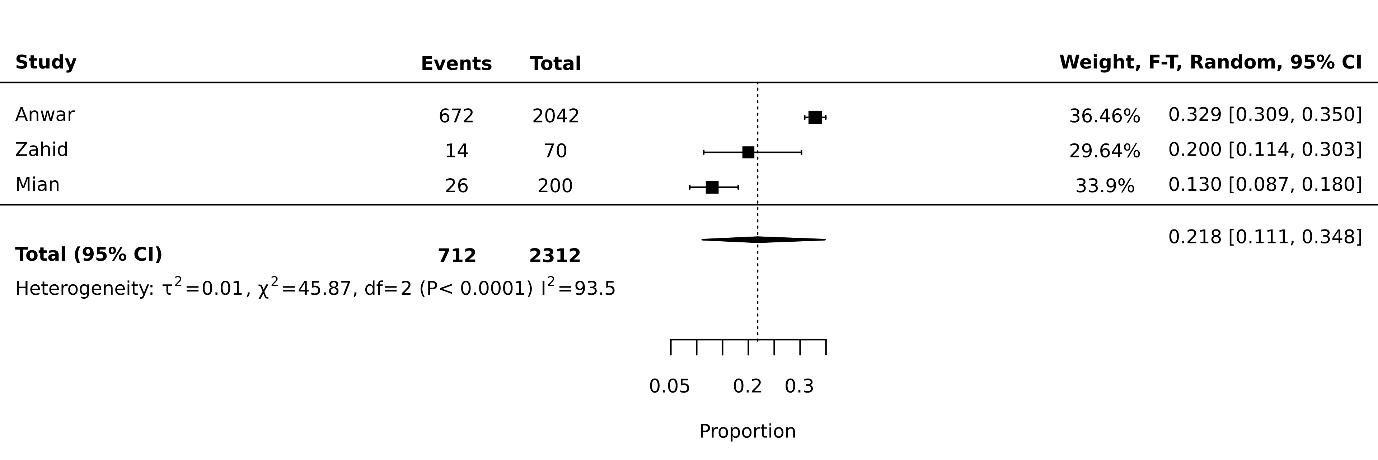


**Fig 7: Thinness**

**Fig 7a: Thinness pooled prevalence in female gender**


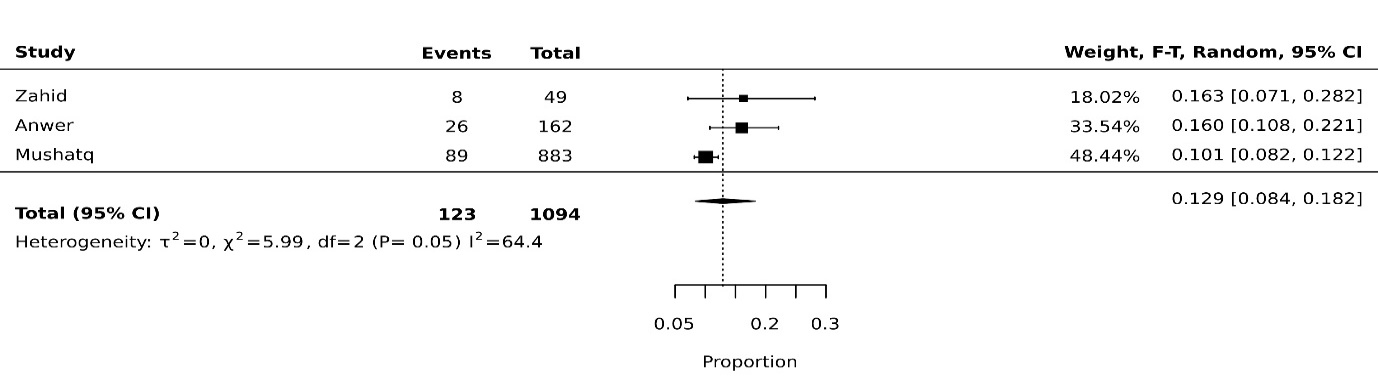
 **Fig 7b: Thinness pooled prevalence in male gender**


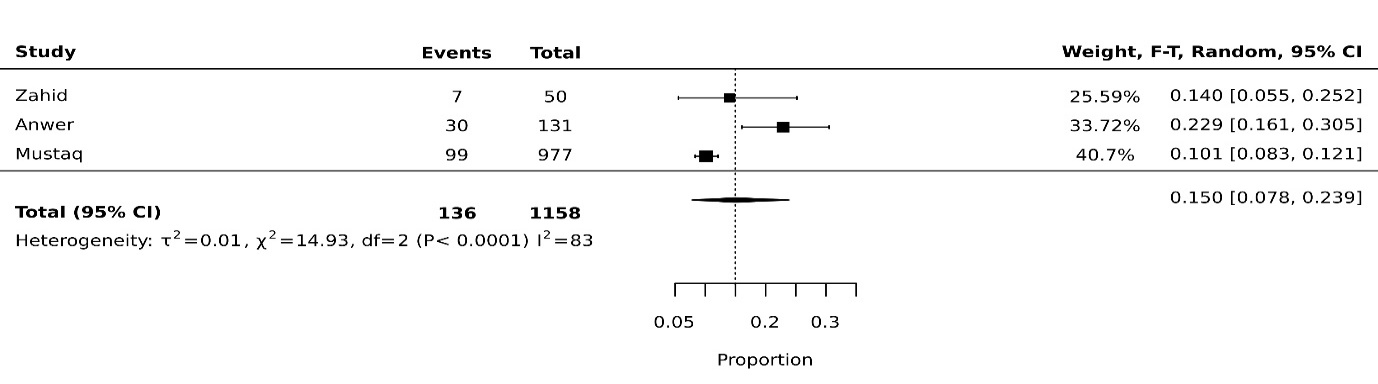
 **Fig 7c: Thinness pooled prevalence in children going to private schools**


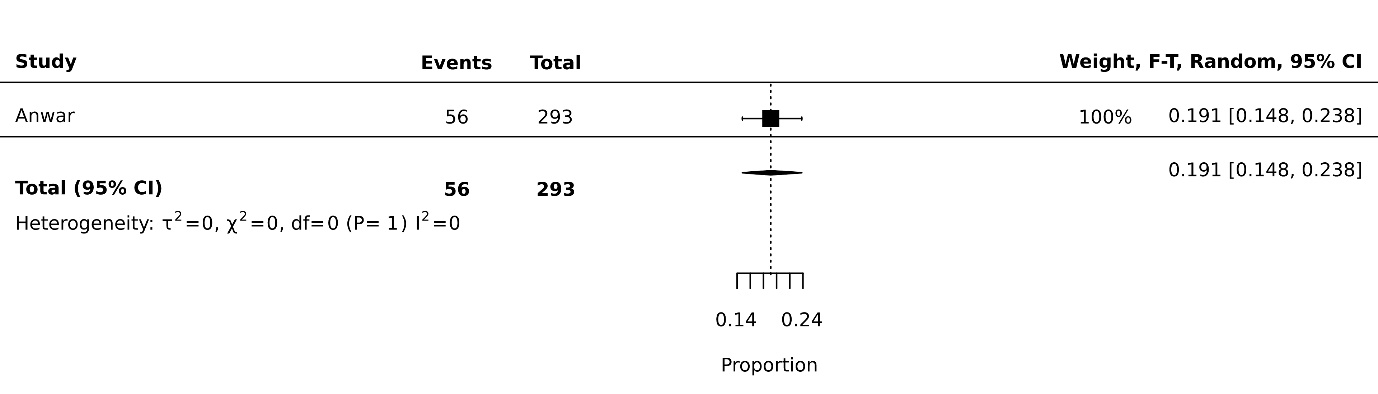


**Fig 7d: Thinness pooled prevalence in children going to government schools**
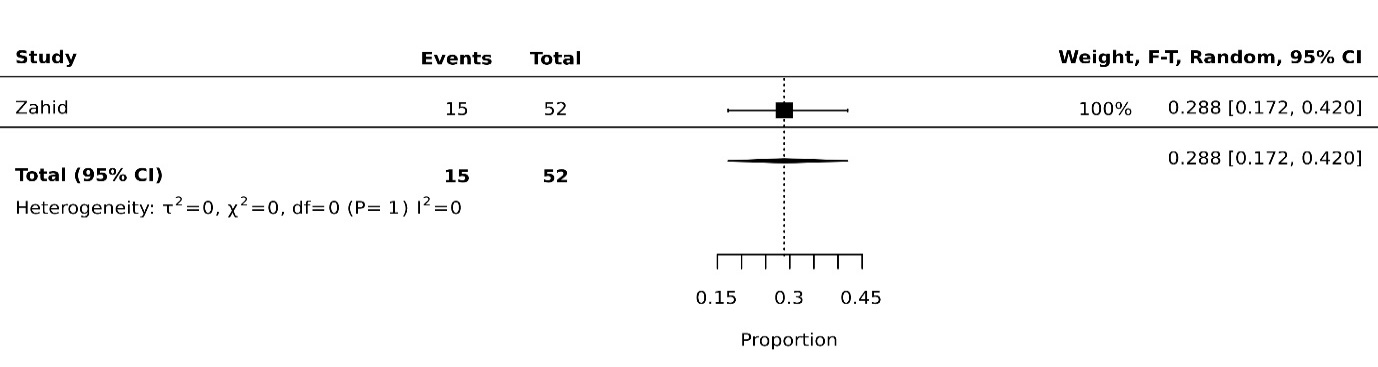
**Fig 7e: Thinness pooled prevalence in children from urban setting**
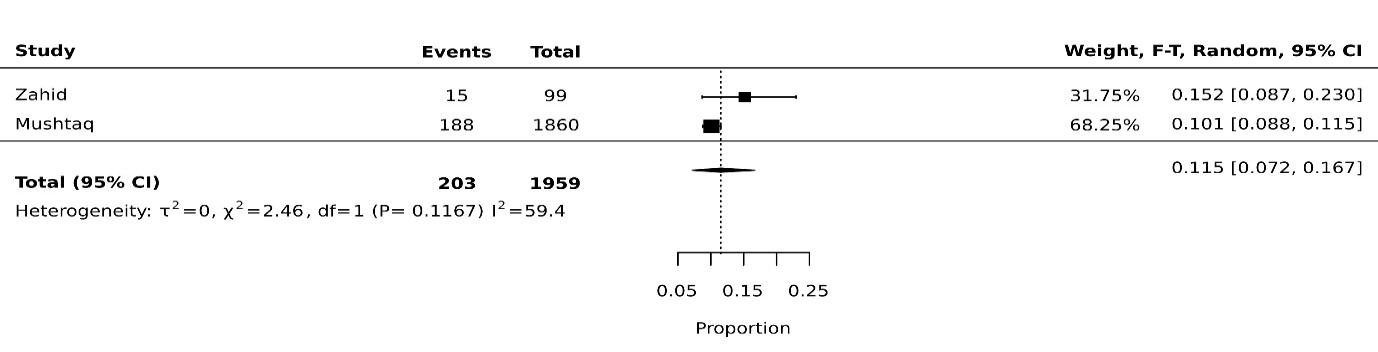
 **Fig 7f: Thinness pooled prevalence in children from low socioeconomic setting**
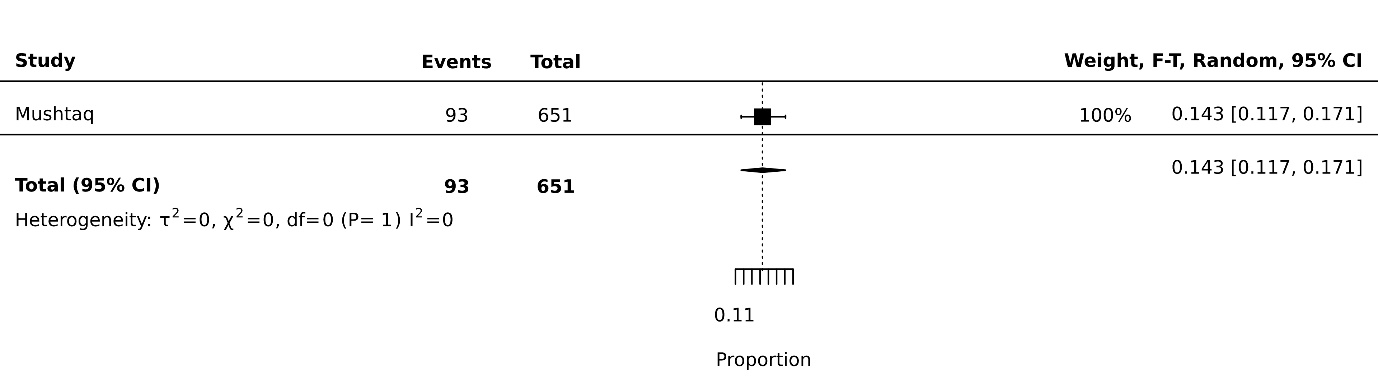


**Fig 7g: Thinness pooled prevalence in children from high socioeconomic setting**
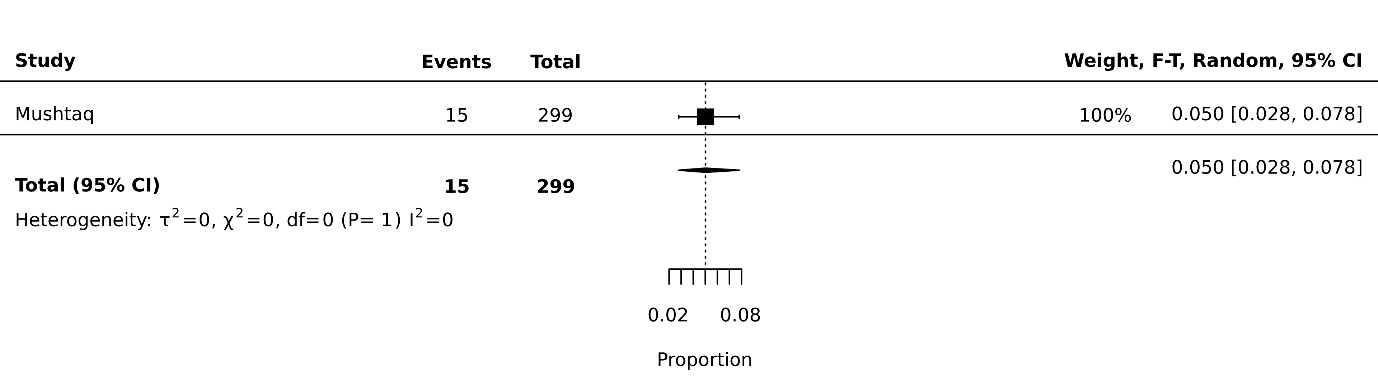


**Fig 7h: Thinness pooled prevalence in children from disaster affected regions**
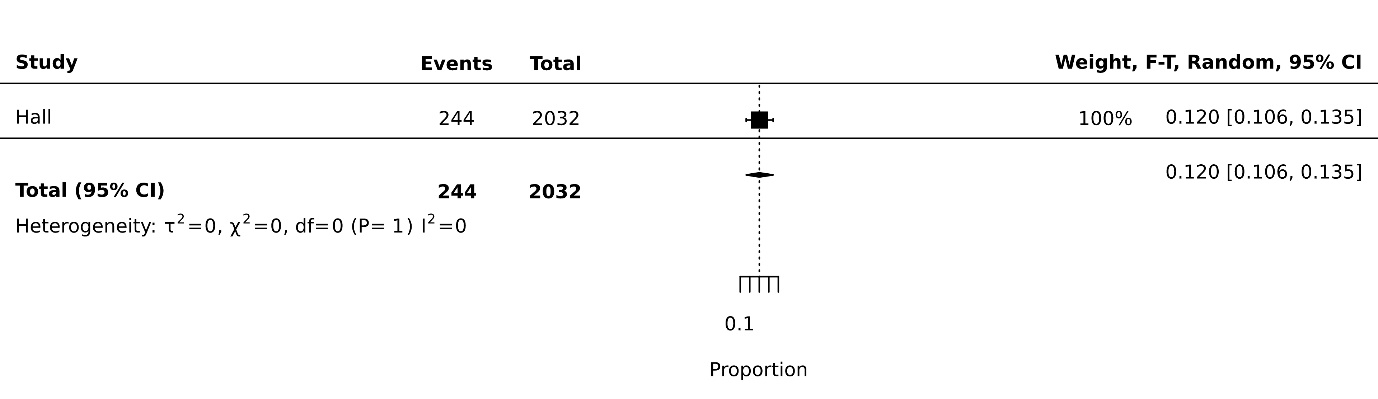


**Fig 7i: Thinness pooled prevalence in children employed as laborers**
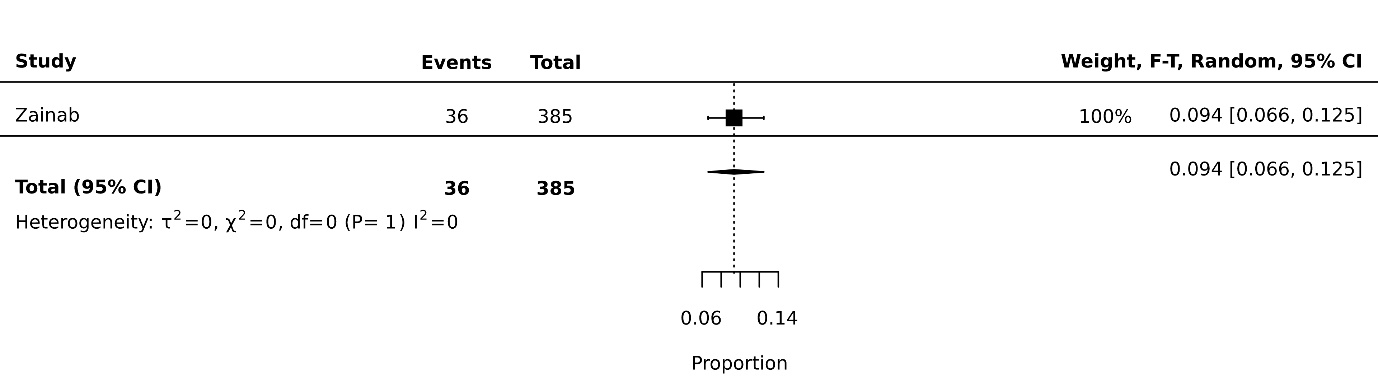


**Fig 7j: Thinness pooled prevalence in children Punjab**


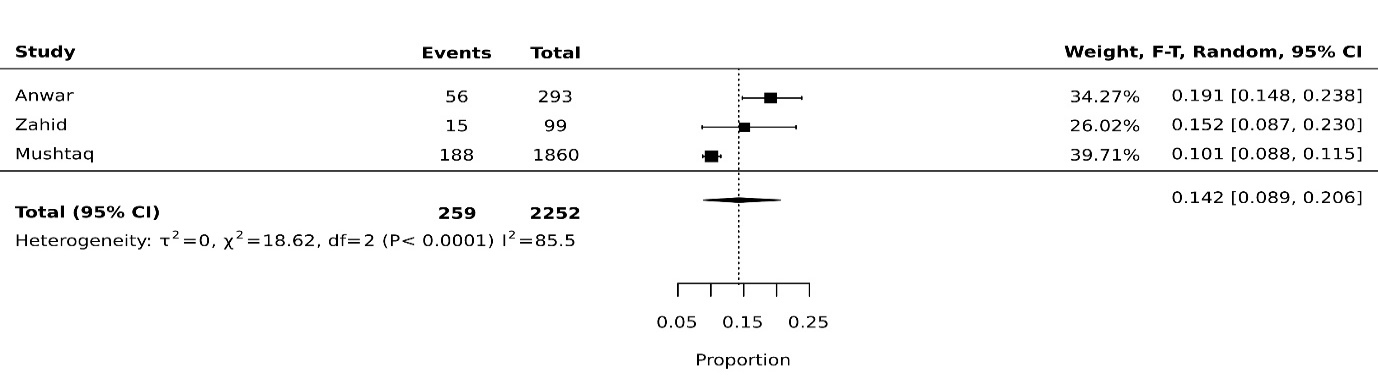


**Fig 8: Overweight**

**Fig 8a: Overweight pooled prevalence in female gender**
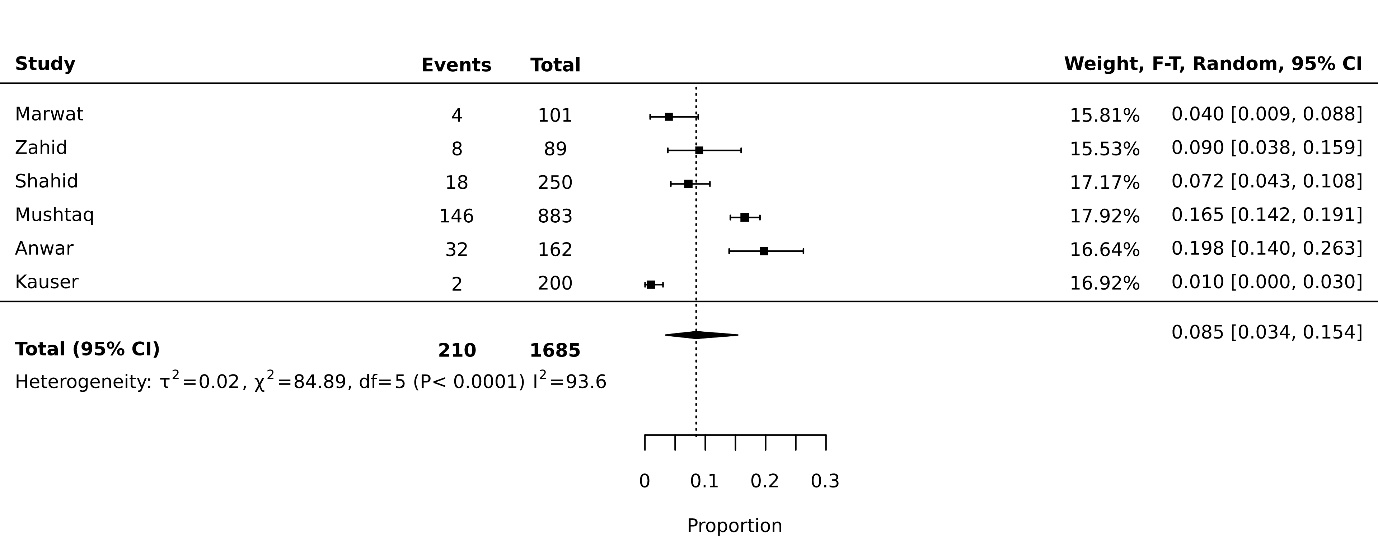


**Fig 8b: Overweight pooled prevalence in male gender**
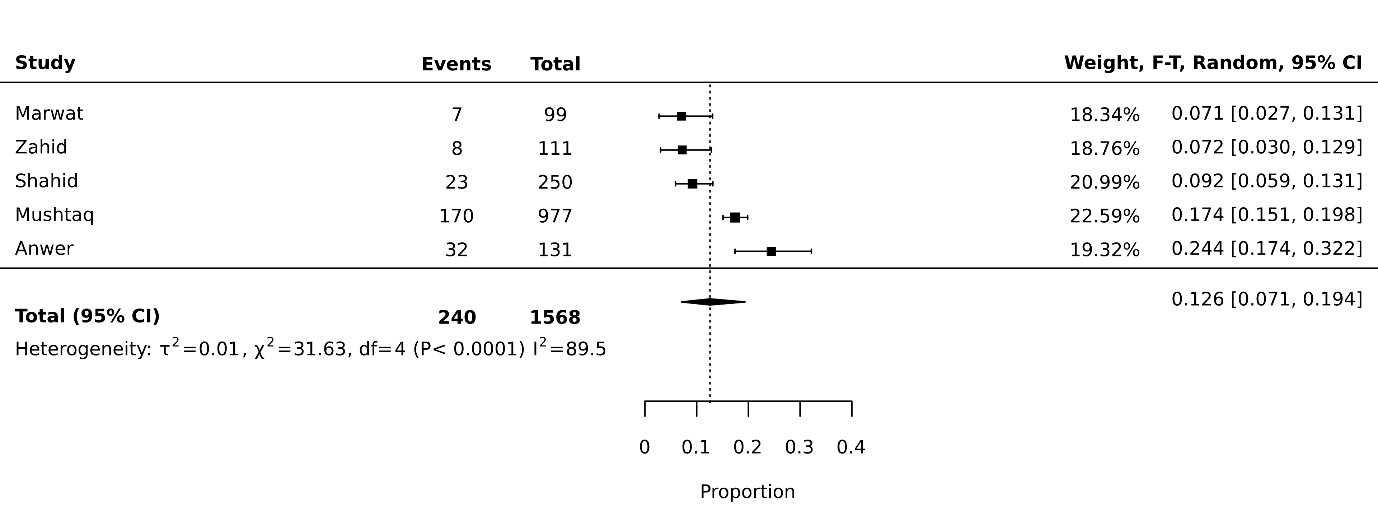


**Fig 8c: Overweight pooled prevalence in children going to private schools**


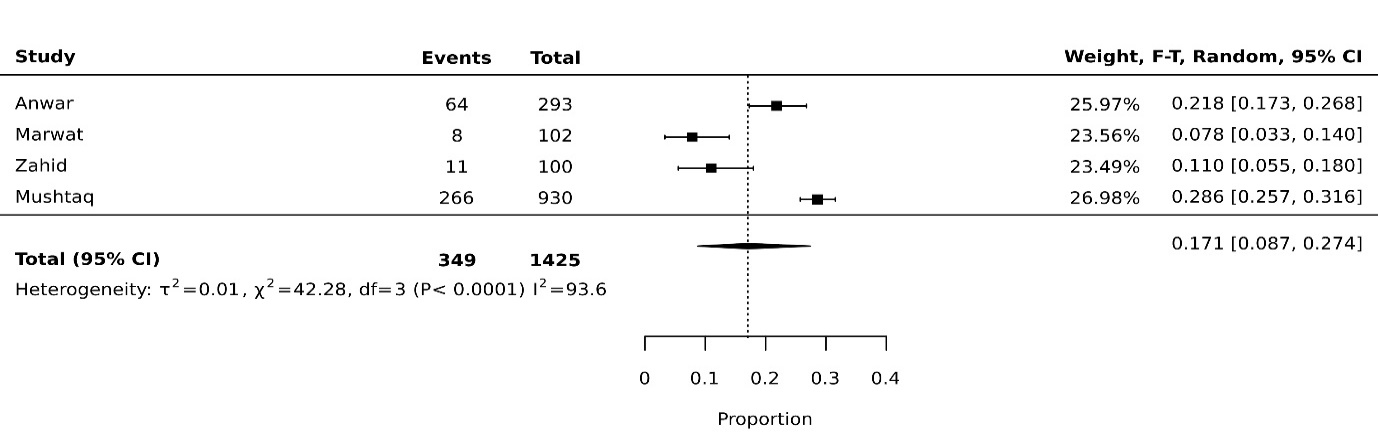


**Fig 8d: Overweight pooled prevalence in children going to government schools**


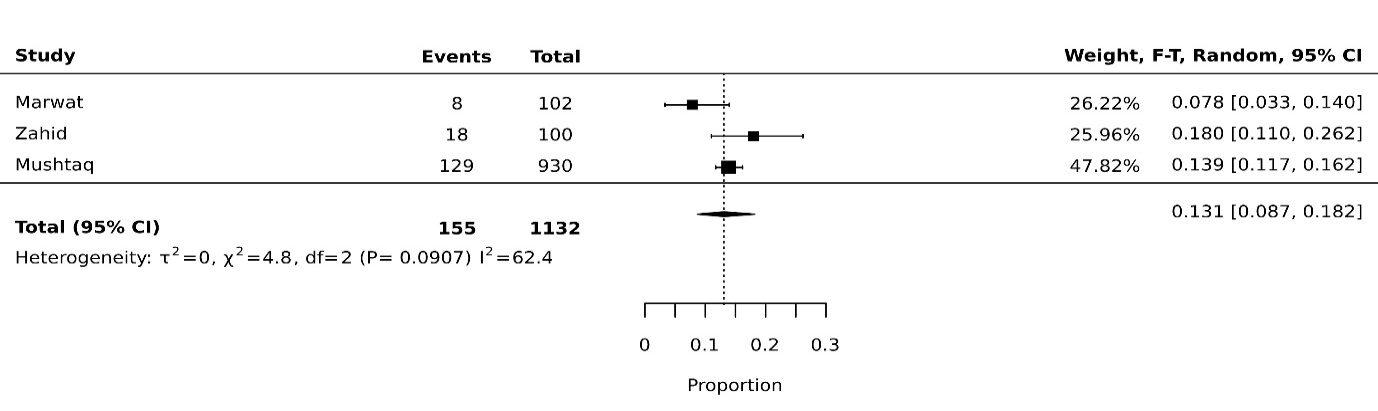


**Fig 8e: Overweight pooled prevalence in children from urban setting**


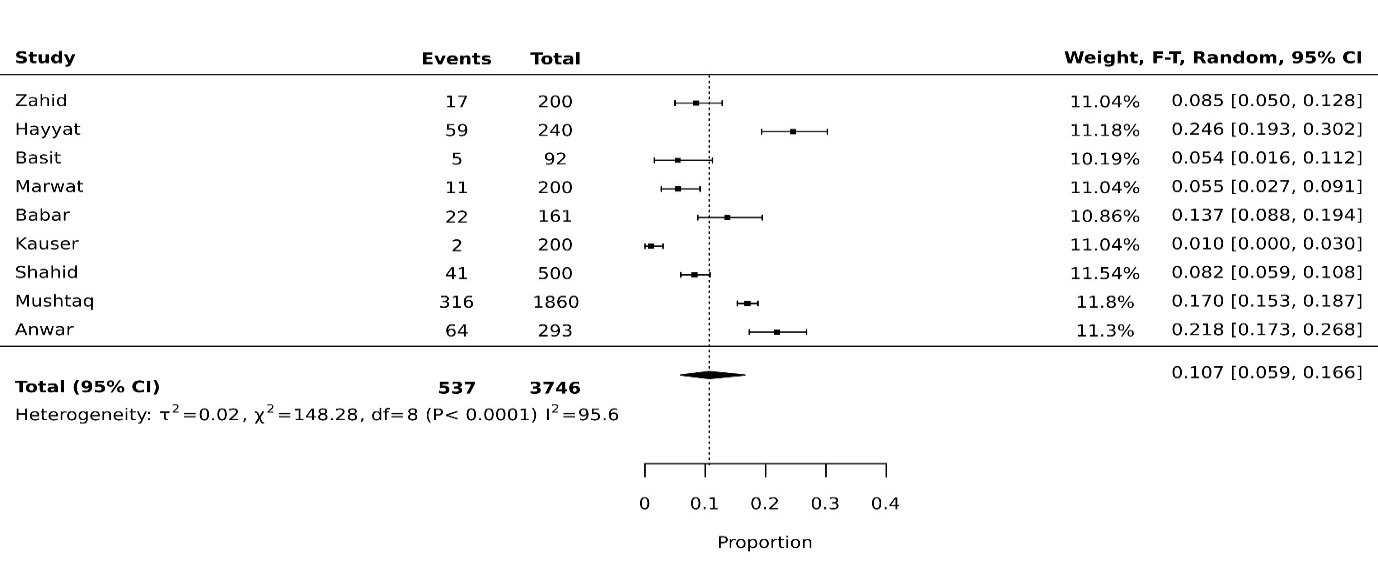


**Fig 8f: Overweight pooled prevalence in children from low socioeconomic setting**


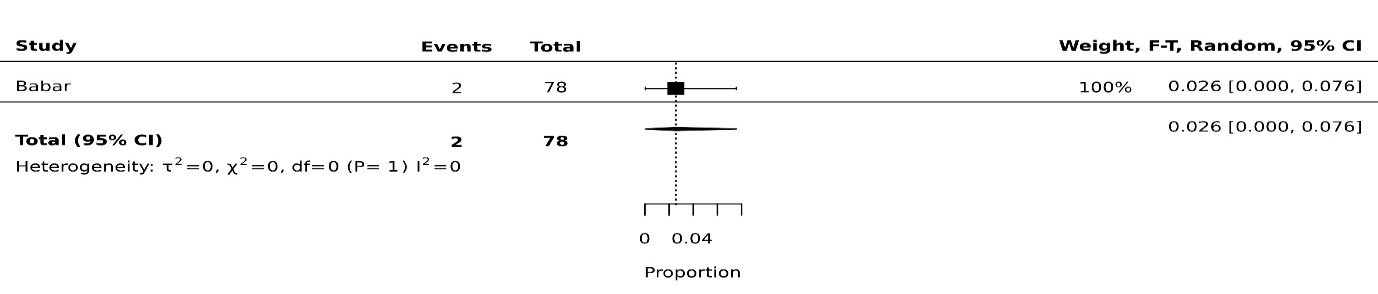


**Fig 8g: Overweight pooled prevalence in children from high socioeconomic setting**


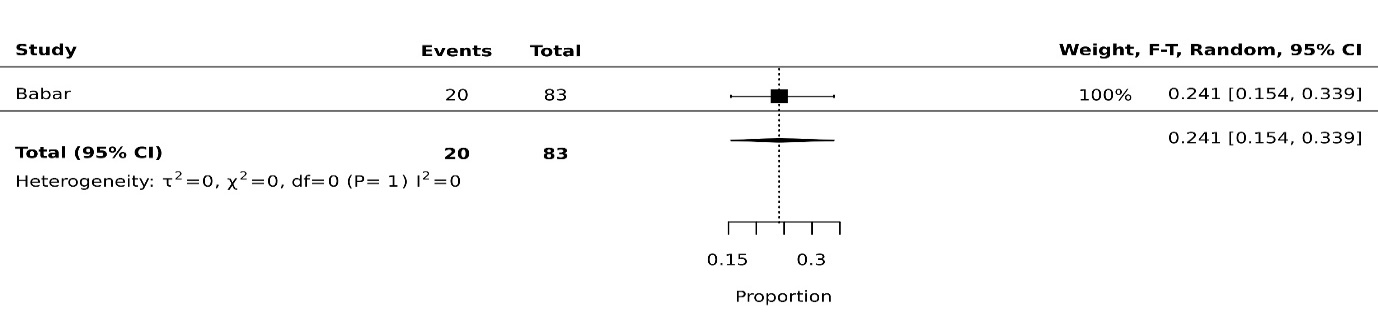


**Fig 8h: Overweight pooled prevalence in children employed as laborers**


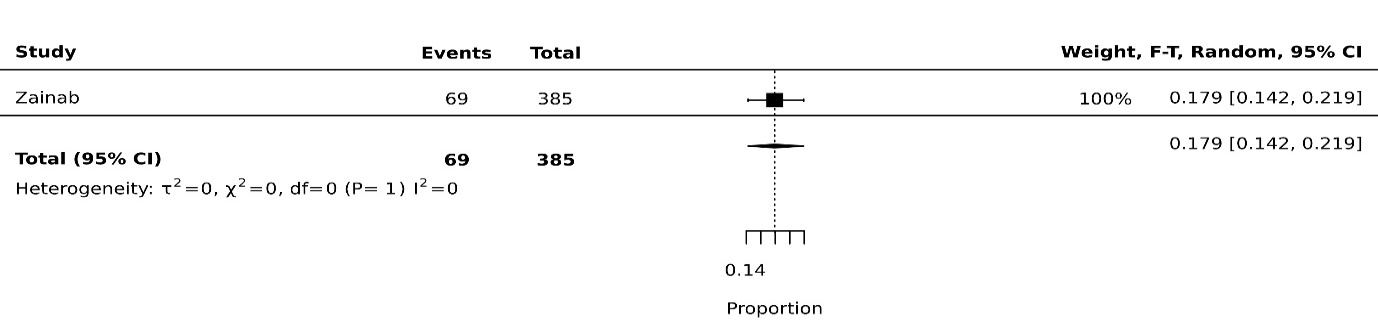


**Fig 8i: Overweight pooled prevalence in children from Sindh**


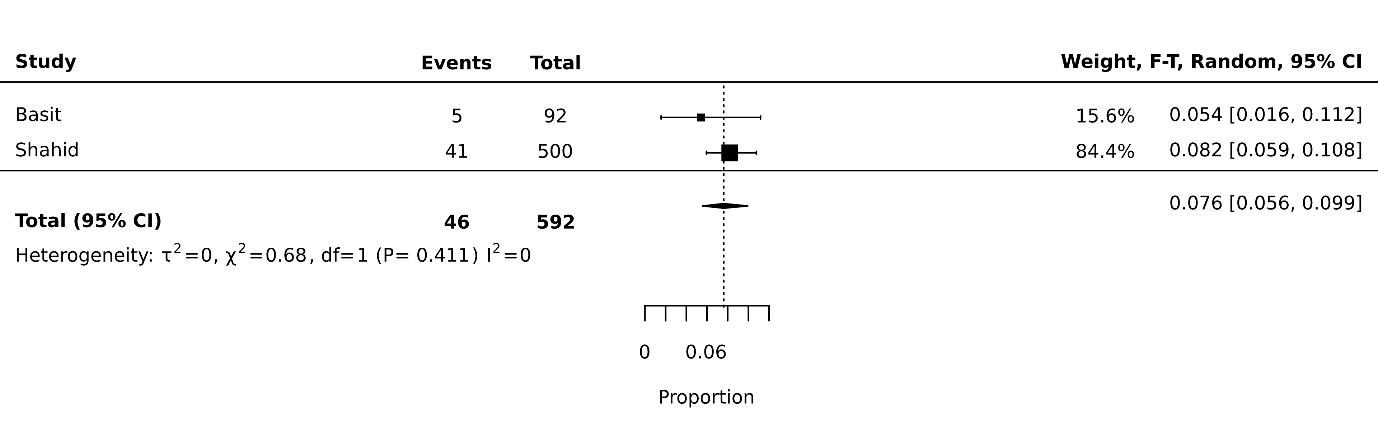


**Fig 8j: Overweight pooled prevalence in children from Punjab**


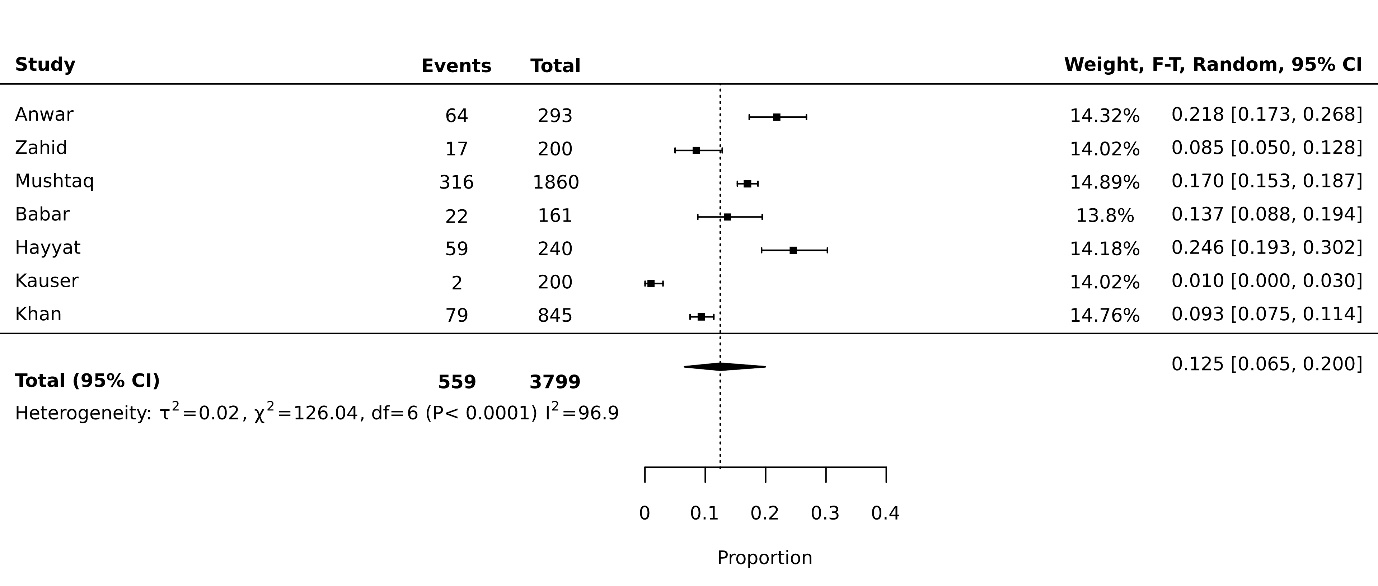
 **Fig 8f: Overweight pooled prevalence in children from KP**


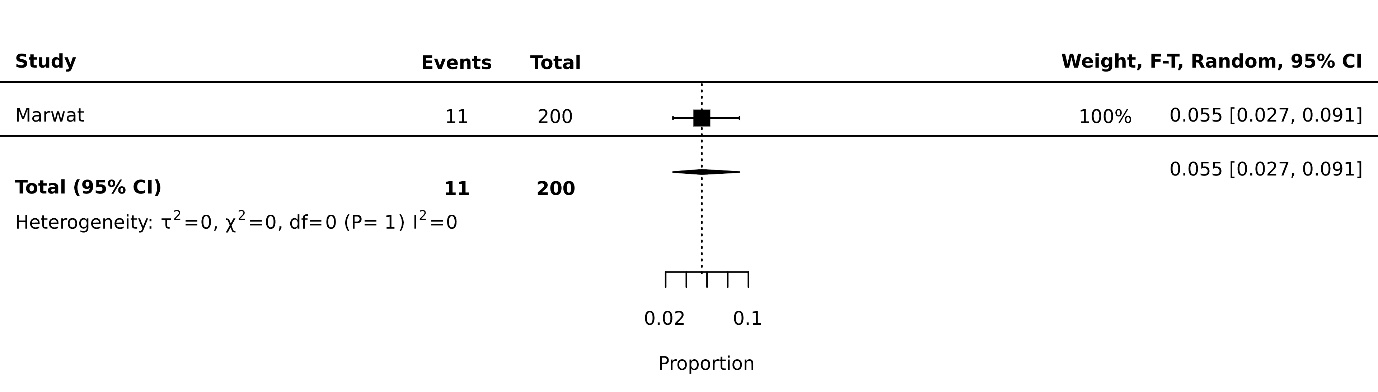


**Fig 9: Obese**

**Fig 9a: Obese pooled prevalence in female gender**


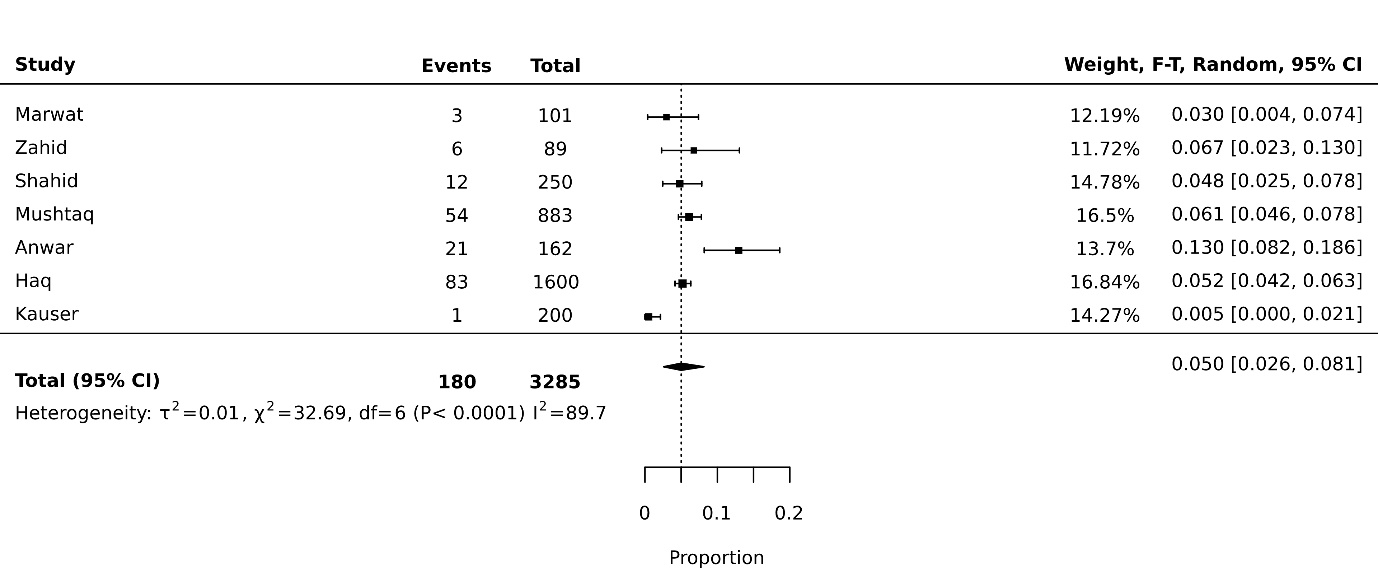


**Fig 9b: Obese pooled prevalence in male gender**


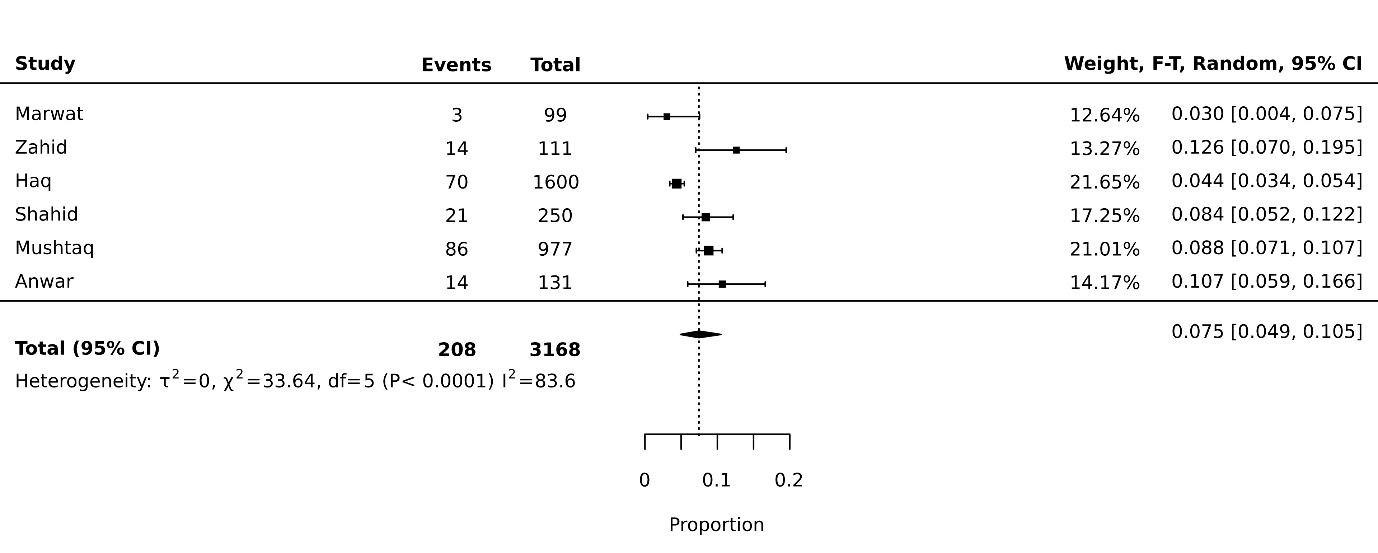


**Fig 9c: Obese pooled prevalence in children going to private schools**


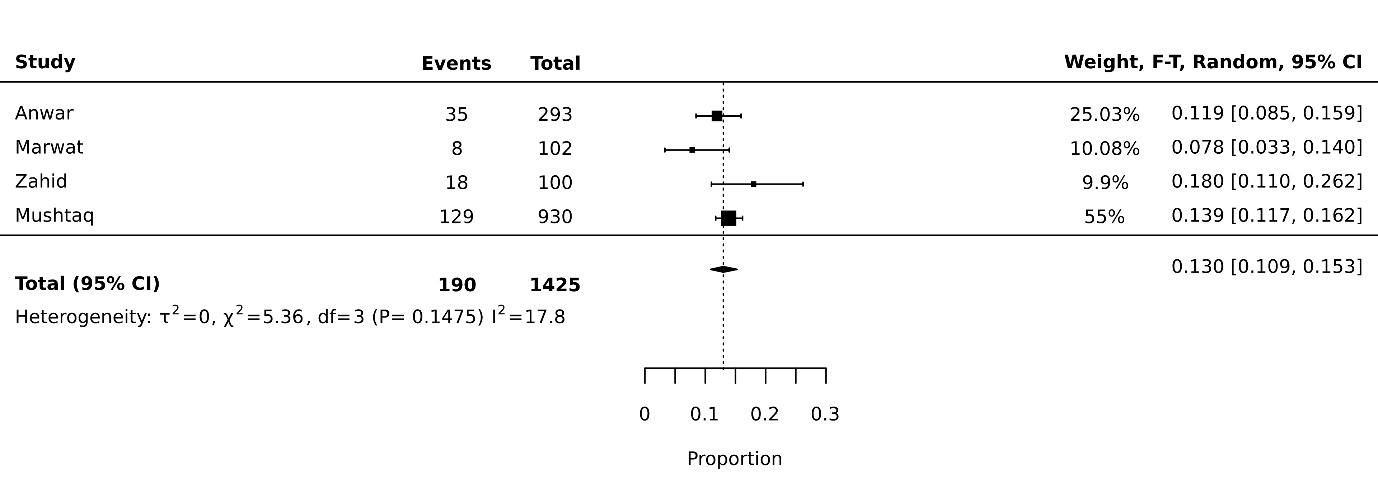


**Fig 9d: Obese pooled prevalence in children going to government schools**


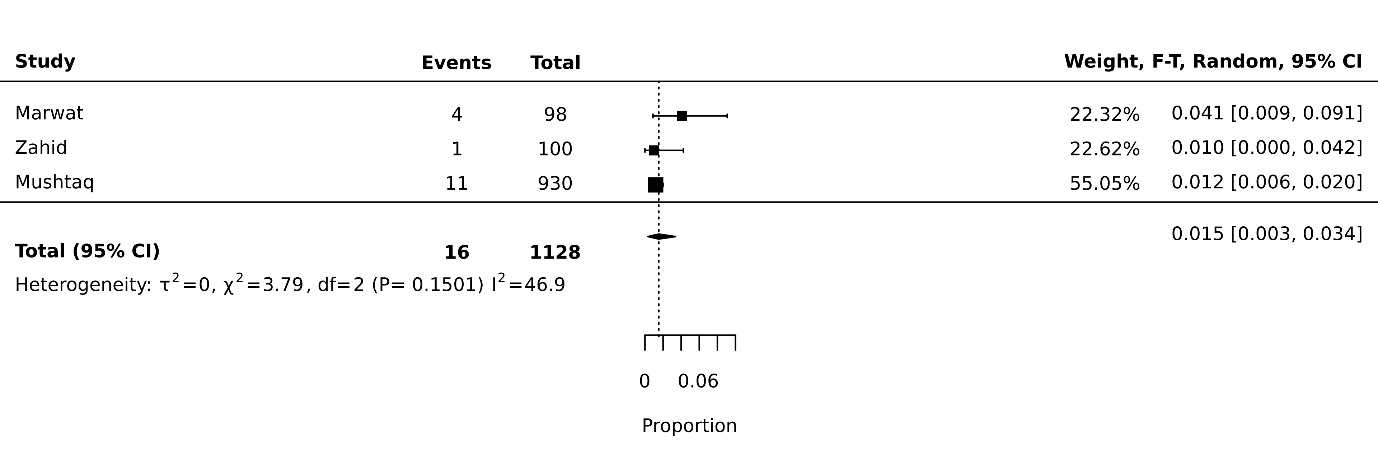


**Fig 9e: Obese pooled prevalence in children from rural setting**


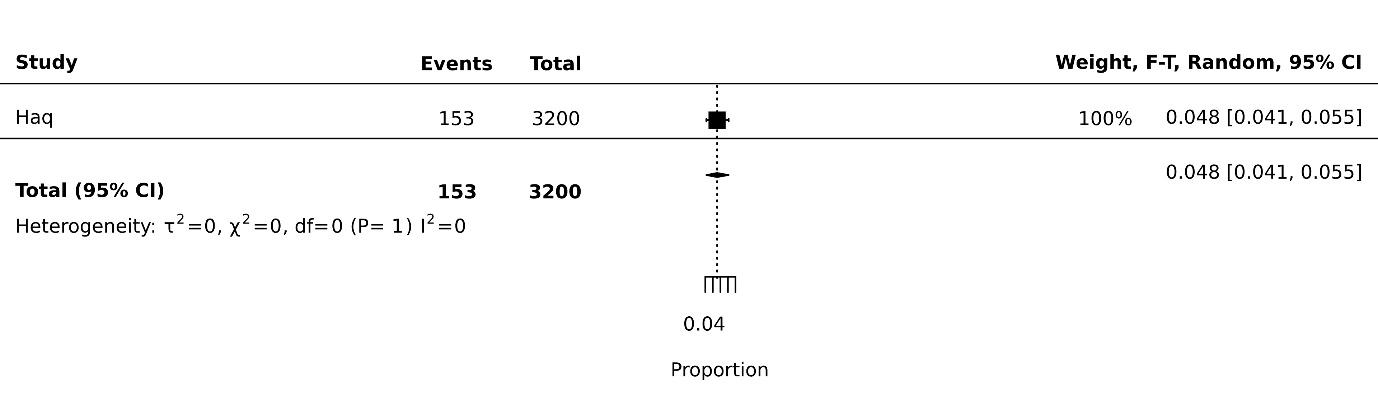


**Fig 9f: Obese pooled prevalence in children from urban setting**


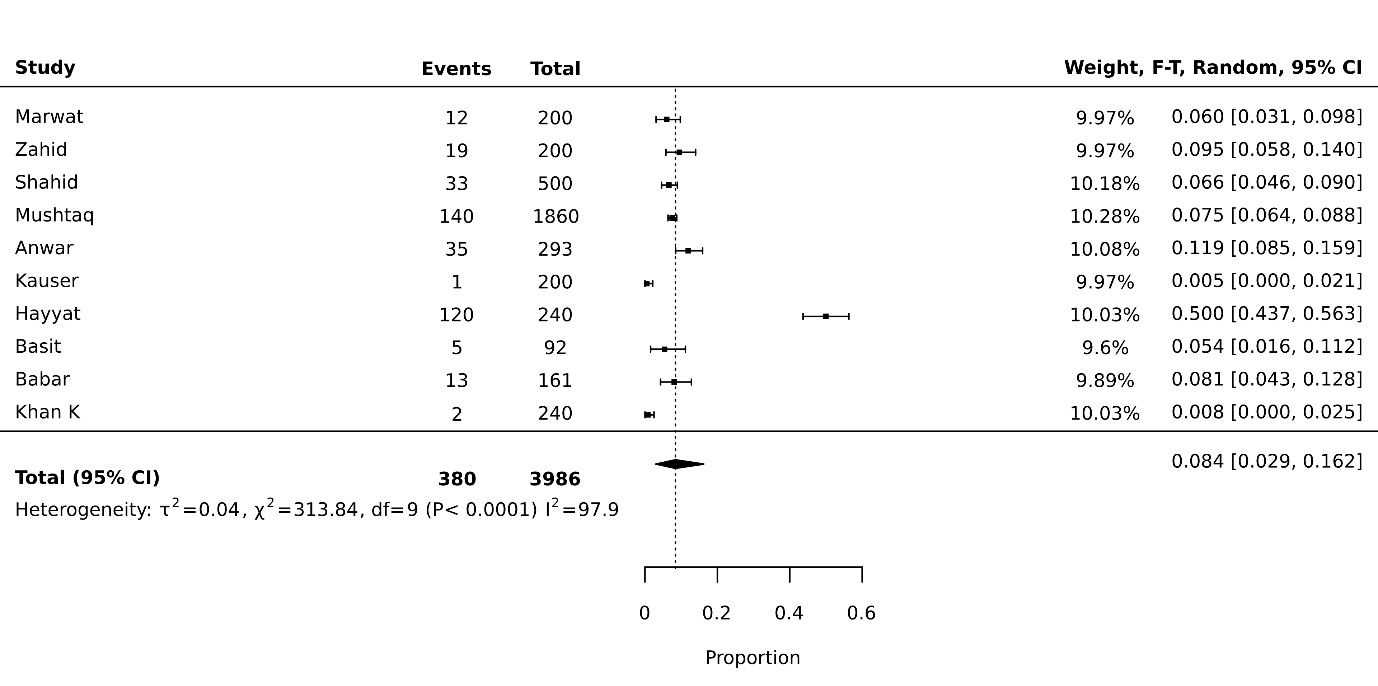


**Fig 9g: Obese pooled prevalence in children from low socioeconomic setting**


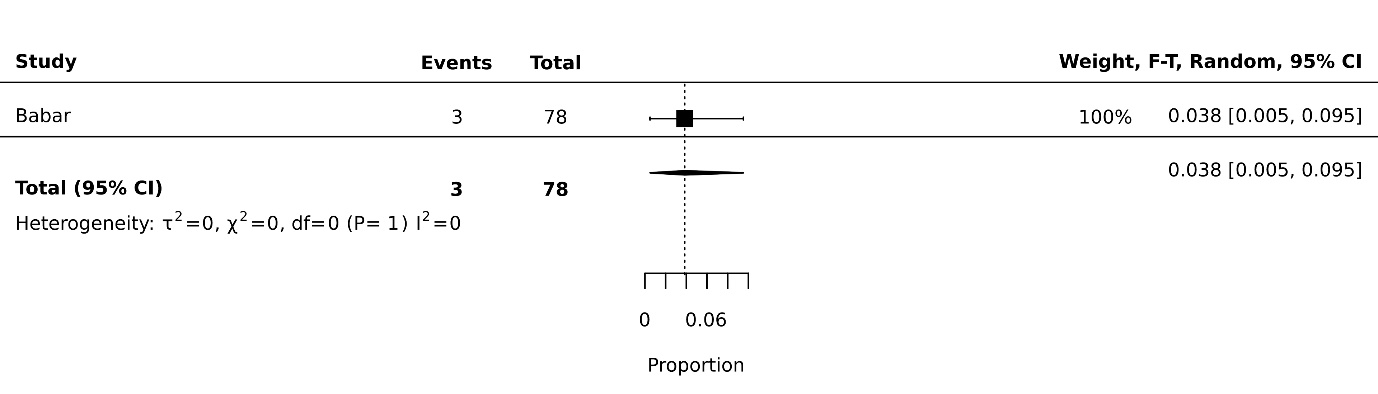


**Fig 9h: Obese pooled prevalence in children from high socioeconomic setting**


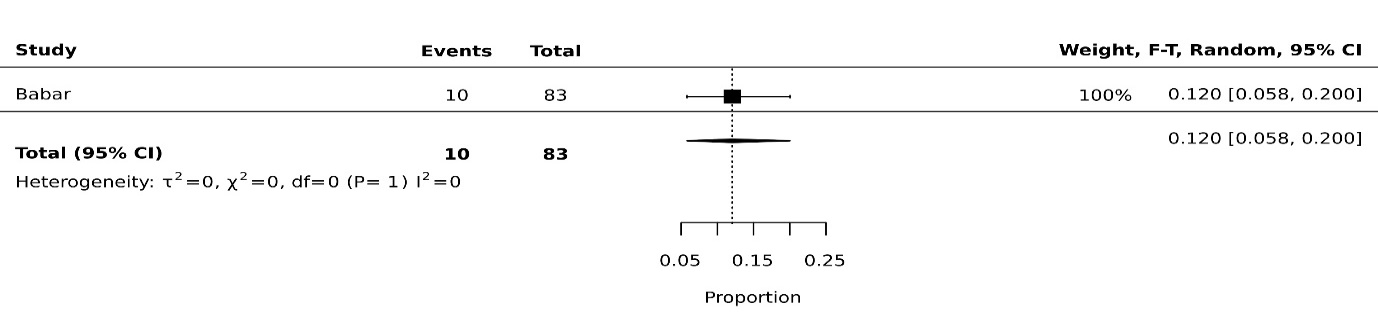


**Fig 9i: Obese pooled prevalence in children employed as laborer**


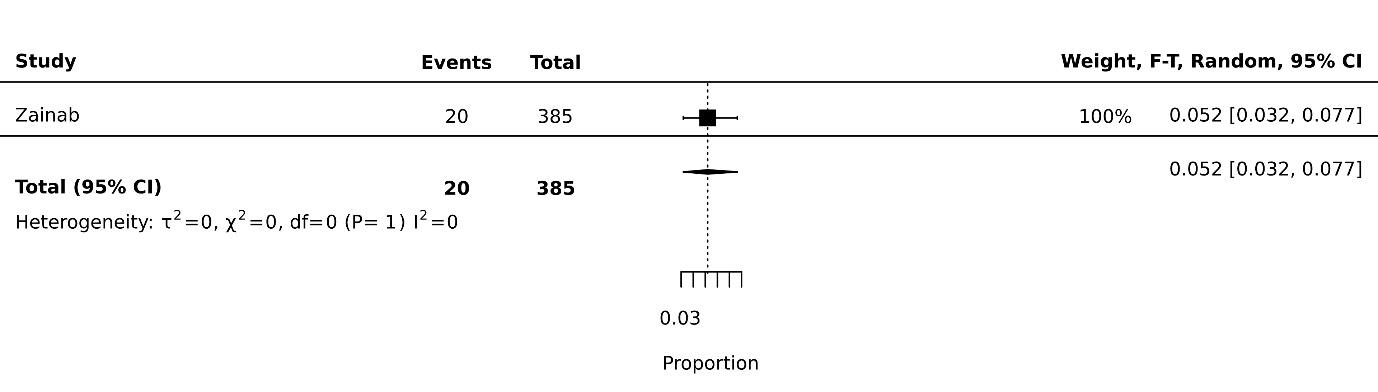


**Fig 9j: Obese pooled prevalence in children from Sindh**


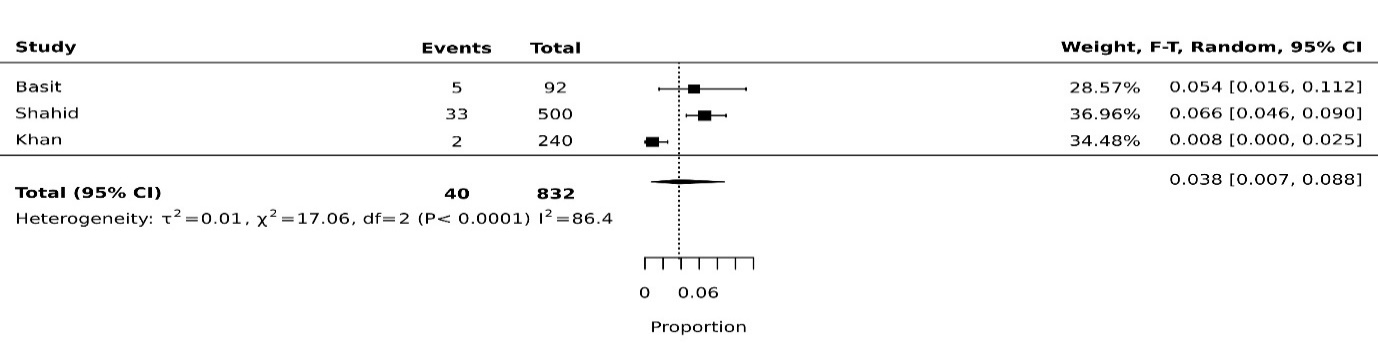


**Fig 9k: Obese pooled prevalence in children from Punjab**


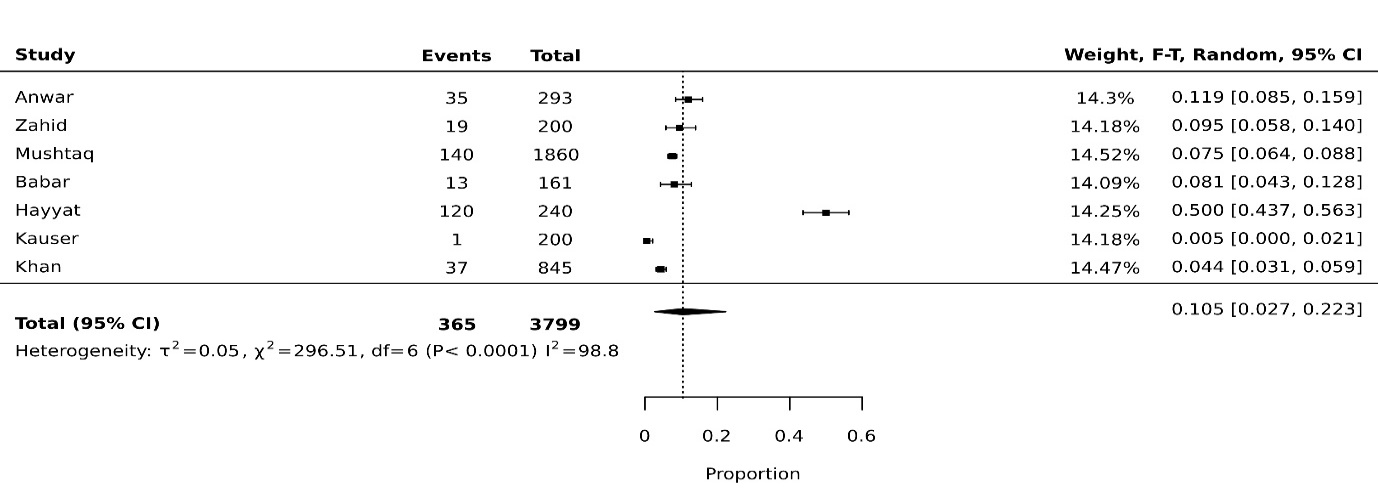


**Fig 9l: Obese pooled prevalence in children from KP**


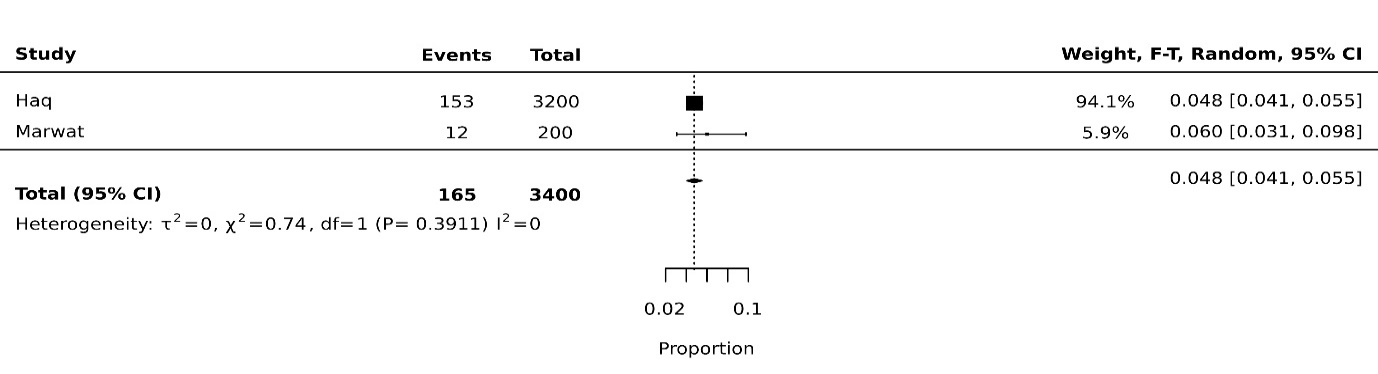


**Fig 10: Pooled prevalence analysis of anthropometric indices in children aged 5 to 10 years**

**Figure 10a: Underweight pooled prevalence in 5 to 10 years**


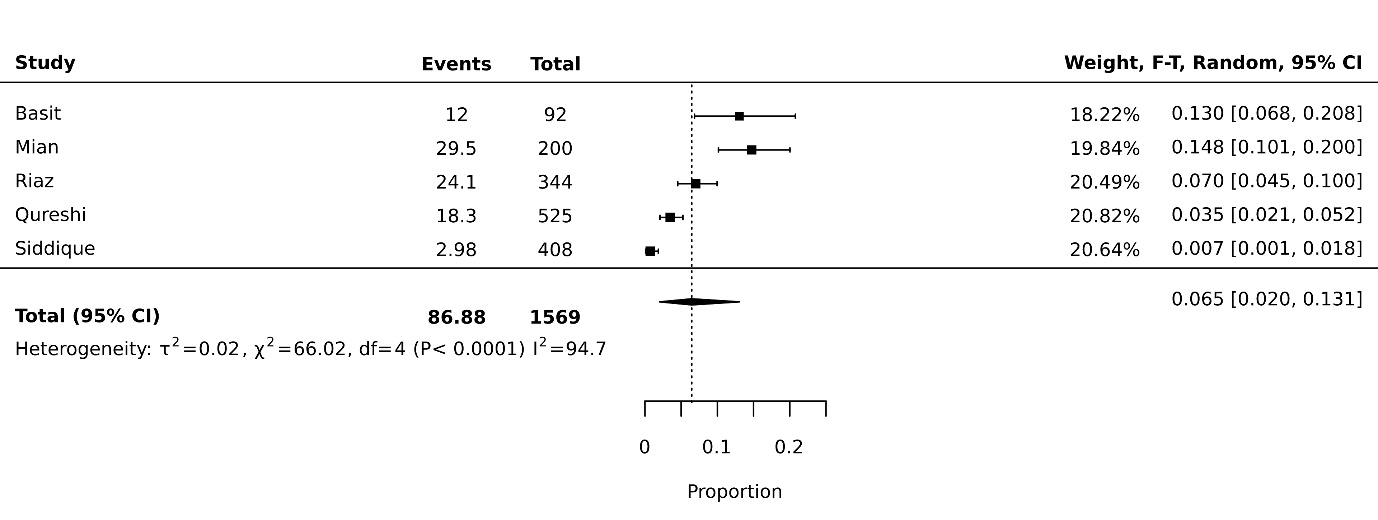


**Figure 10b: Stunting pooled prevalence in 5 to 10 years**
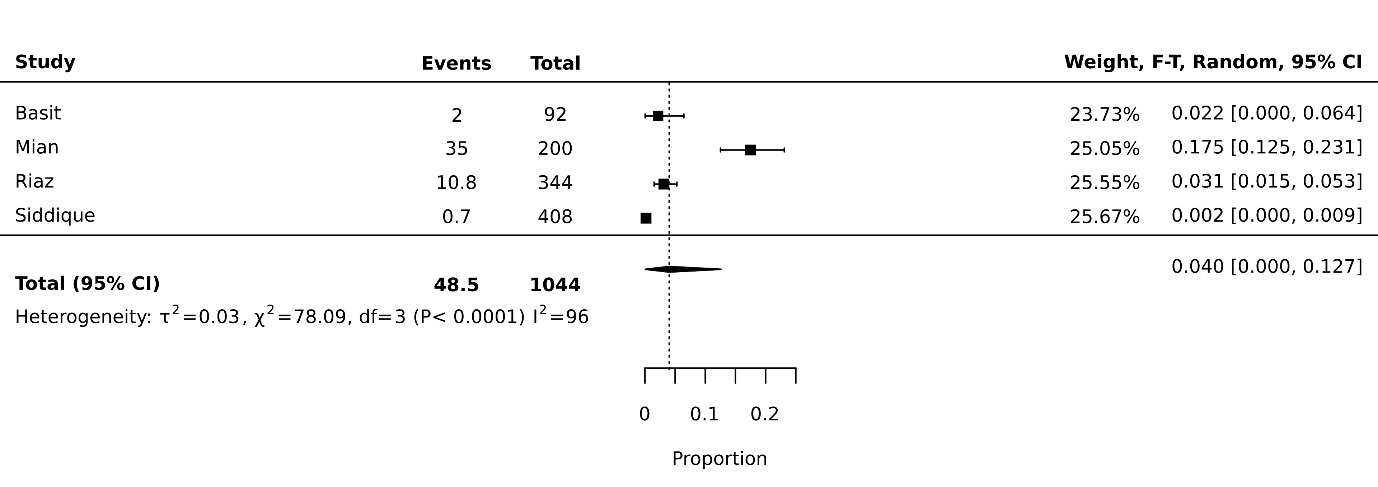


**Fig 11: Pooled prevalence analysis of anthropometric indices in children aged 10 to 15 years**

**Figure 11a: Overweight pooled prevalence in 10 to 15 years**


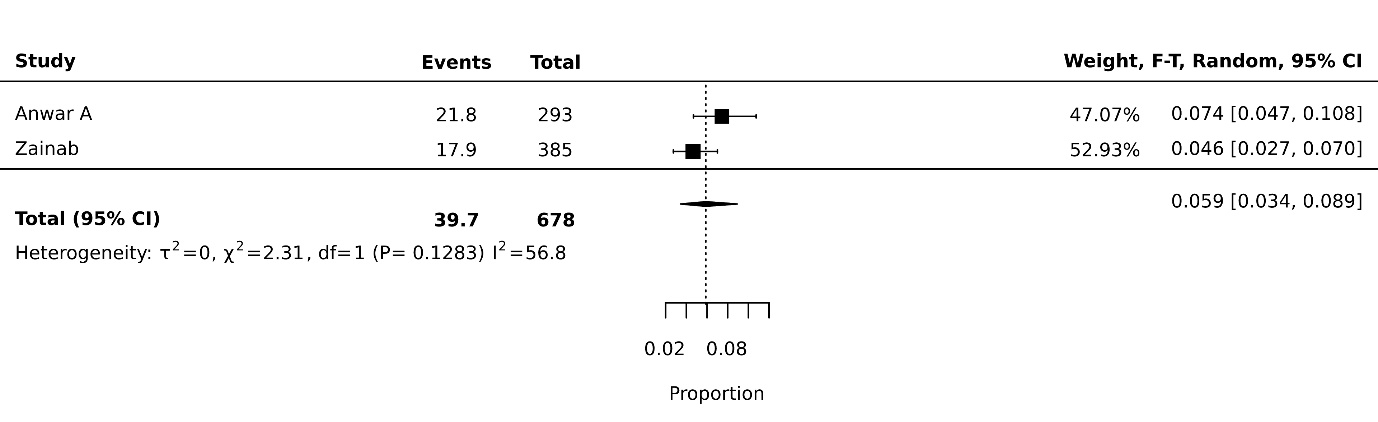


**Figure 11b: Obese pooled prevalence in 10 to 15 years**


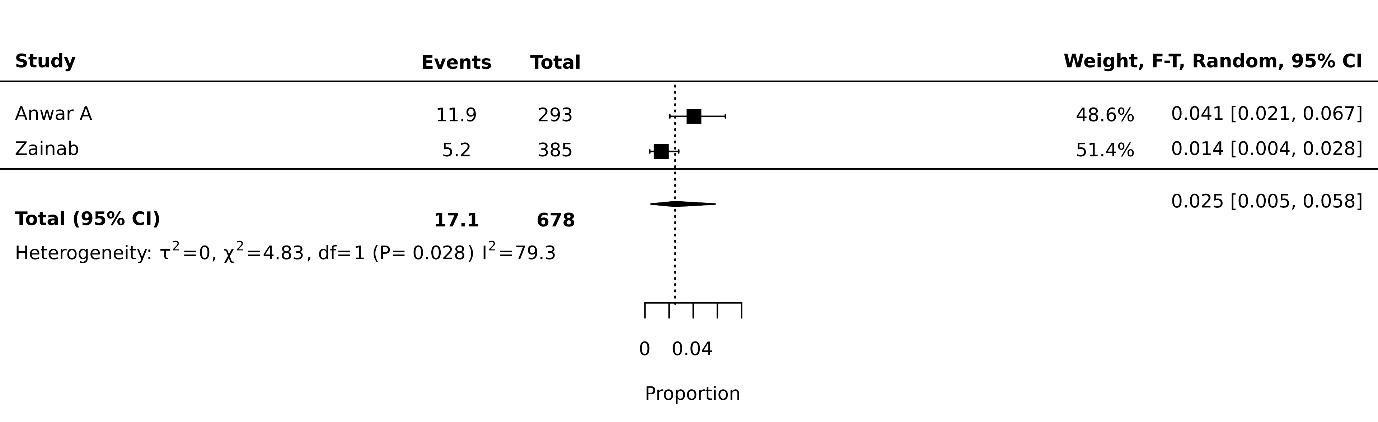

Supplement: Supplementary file 1 [file Data_Sheet_1.docx]
